# Supplementary material for: Potent DNA gyrase inhibitors bind asymmetrically to their target using symmetrical bifurcated halogen bonds
Source: Nat Commun. 2021 Jan 8;12:150. doi: 10.1038/s41467-020-20405-8 (PMC7794245; doi:10.1038/s41467-020-20405-8)
Supplement: Supplementary file 1 — Supplementary Information [file 41467_2020_20405_MOESM1_ESM.pdf]

Supplementary Information

Potent DNA gyrase inhibitors bind asymmetrically to their target using symmetrical bifurcated halogen bonds

Anja Kolarič, Thomas Germe, Martina Hrast, Clare E. M. Stevenson, David M. Lawson, Nicolas Burton, Judit Vörös, Anthony Maxwell, Nikola Minovski*, Marko Anderluh*

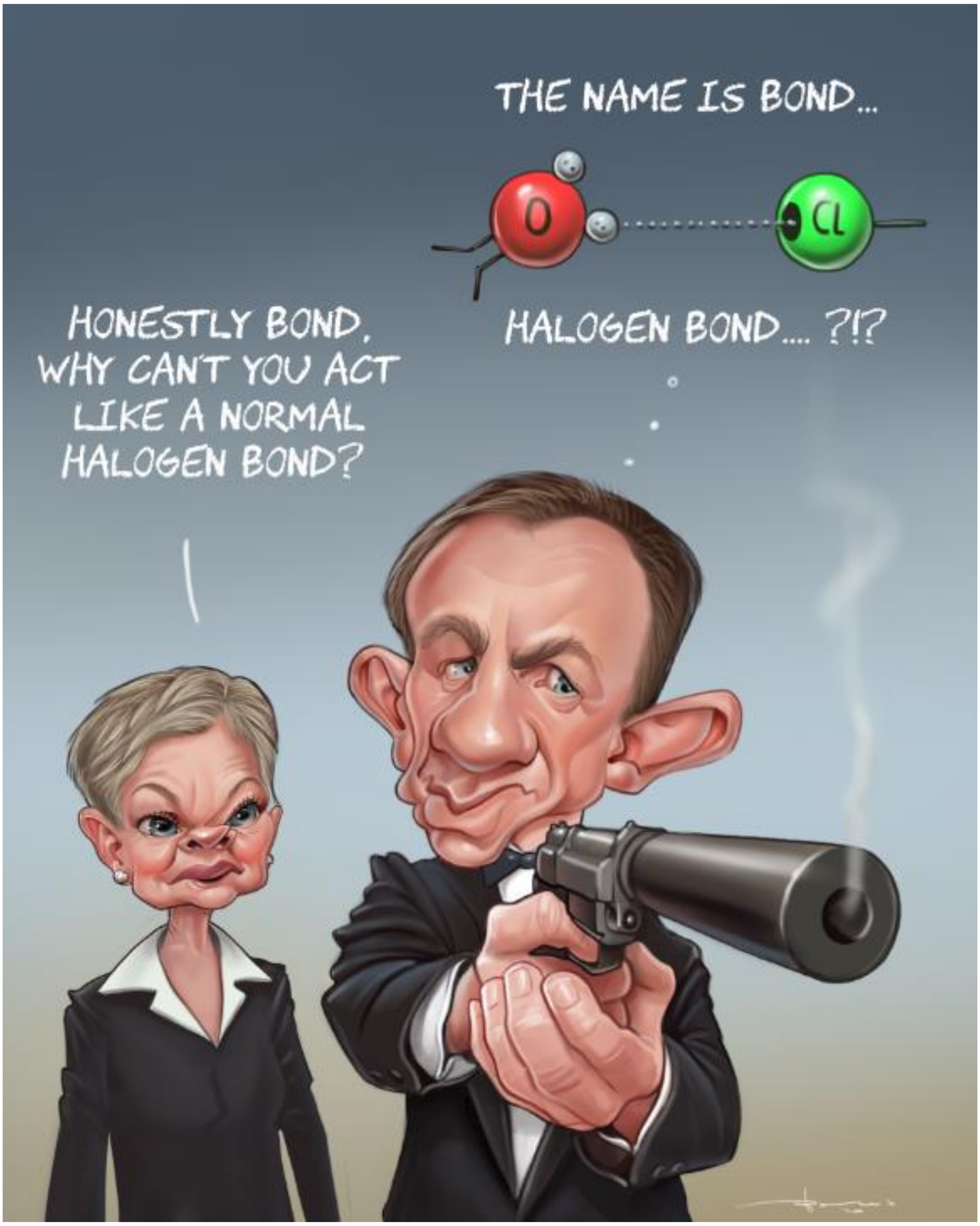

Supplementary Table S1. X-Ray Data Collection and Refinement Statistics.

Inhibitor	**4**
Data collection	
Beamline	I04 Diamond Light Source, UK
Wavelength (Å)	0.9795
Detector	Eiger2 X 16M
Resolution range (Å)	81.09 – 2.30 (2.34 – 2.30)
Space Group	$P6_1$
Cell parameters (Å)	$a = b = 92.6$, $c = 405.5$
Total no. of measured intensities	1751993 (70928)
Unique reflections	86845 (4546)
Multiplicity	20.2 (15.6)
Mean $I/\sigma(I)$	11.7 (1.4)
Completeness (%)	100.0 (99.5)
R_{merge}[a]	0.193 (2.017)
R_{meas}[b]	0.198 (2.085)
$CC_{½}$[c]	0.998 (0.557)
Wilson B value (Å^2)	41.2
Refinement	
Resolution range (Å)	80.32–2.30 (2.36-2.30)
Reflections: working/free[d]	82229/4484
R_{work}/ R_{free}[e]	0.179/0.217 (0.278/0.314)
Ramachandran plot: favoured/allowed/disallowed[f] (%)	97.1/2.8/0.1
R.m.s. bond distance deviation (Å)	0.010
R.m.s. bond angle deviation (°)	1.636
No. protein residues	B chain:672; D chain:670
No. DNA bases	E/F chains:8; G/H chains:12
No. of water/ligands/ions	343/6/5
Mean B factors: protein/DNA/water/ligands/ions/overall (Å^2)	49/52/44/75/62/49
RSCC score[g] for 4	0.90
Accession code	6Z1A

Values in parentheses are for the outer resolution shell.

[a] $Rmerge = \sum hkl \sum i \, |Ii(hkl) - \langle I(hkl)\rangle| / \sum hkl \sum iIi(hkl)$.

[b] $meas = \sum hkl \, [N/(N-1)]^{1/2} \times \sum i \, |Ii(hkl) - \langle I(hkl)\rangle| / \sum hkl \sum iIi(hkl)$, where $I_i(hkl)$ is the ith observation of reflection hkl, $\langle I(hkl)\rangle$ is the weighted average intensity for all observations i of reflection hkl and N is the number of observations of reflection hkl.

[c] $CC_{½}$ is the correlation coefficient between symmetry equivalent intensities from random halves of the dataset.

[d] The data set was split into "working" and "free" sets consisting of 95 and 5% of the data respectively. The free set was not used for refinement.

[e] The R-factors R_{work} and R_{free} are calculated as follows: $R = \sum(|Fobs - Fcalc|)/\sum|Fobs|$, where F_{obs} and F_{calc} are the observed and calculated structure factor amplitudes, respectively.

[f] From MolProbity.[27]

[g] From the PDB validation server.

Molecular Docking

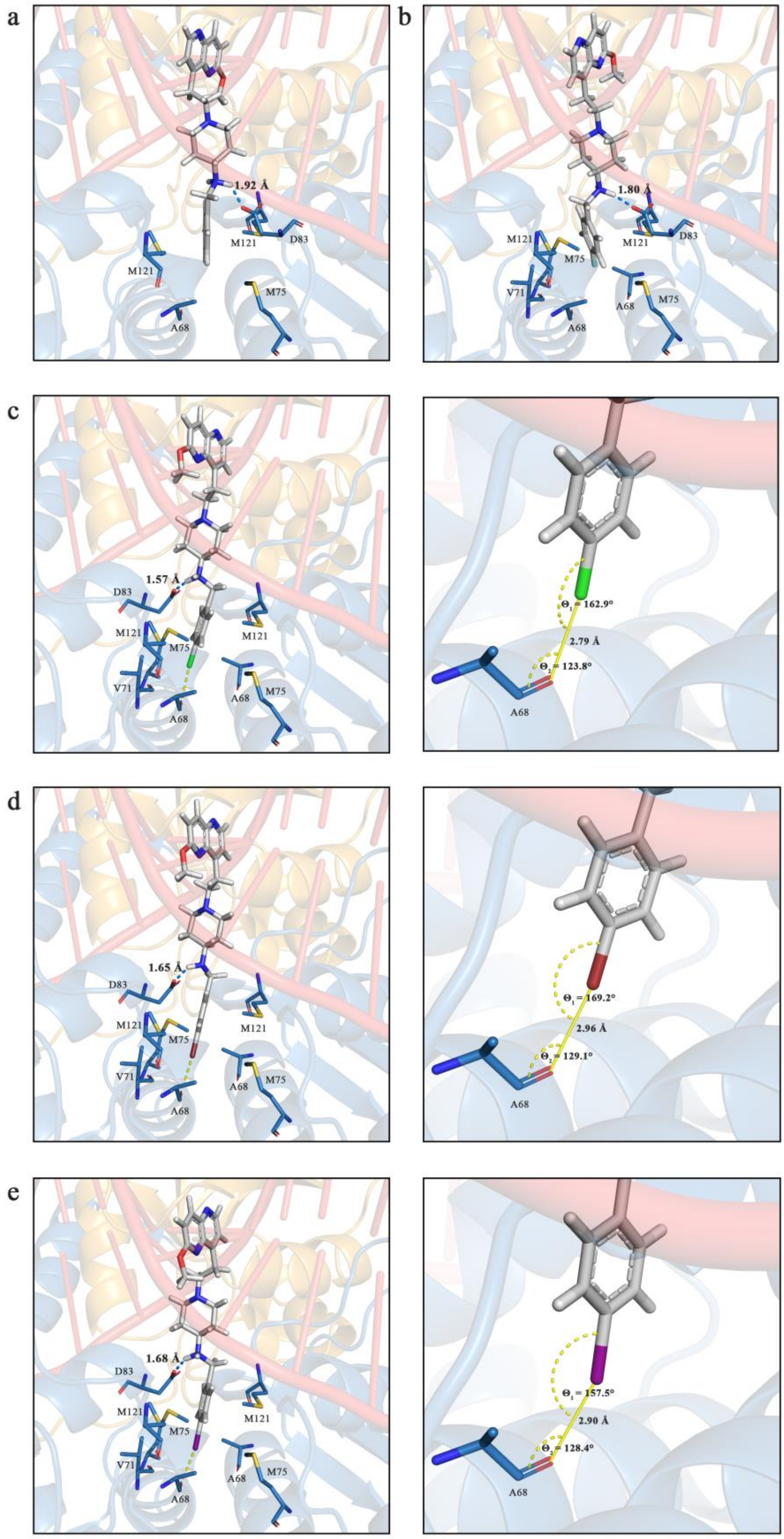

Supplementary Figure 1: Predicted binding modes of compounds 2-6. Molecular docking results representing compounds (sticks representation colour-coded by element) with differently substituted phenyl RHSs, within the *S. aureus* DNA gyrase NBTI-binding site (PDB ID: 2XCS)[1], revealing key intermolecular interactions. **a** compound **2** (unsubstituted phenyl); **b** compound **3** (*p*-fluoro phenyl, fluorine atom in cyan); **c** compound **4** (*p*-chloro phenyl, chlorine atom in green); **d** compound **5** (*p*-bromo phenyl, bromine atom in red); **e** compound **6** (*p*-iodo phenyl, iodine atom in violet). Hydrogen bonds are represented as blue dots, while predicted X···O halogen bonding interactions as yellow dots. GyrA, GyrB and DNA molecules are represented as cartoons in blue, yellow, and red, respectively. The amino acid residues are represented as sticks colour-coded by element.

Supplementary Table 2. Distance and angle parameters from predicted binding modes (Fig. S1), indicating the ability for the halogen bonds formation between *p*-halogenated inhibitors 4-6 (Cl, Br, and I) and Ala68 backbone carbonyl oxygen from one GyrA subunit.

**Compound**	**distance (Å)**<br>**X···O**	**Θ_1 angle (°)**<br>**C-X···O**	**Θ_2 angle (°)**<br>**X···O=C**
**4**	2.79	162.9	123.8
**5**	2.96	169.2	129.1
**6**	2.90	157.5	128.4

Molecular dynamics simulations

All-atom molecular dynamics simulations on apo (ligand-free) and ligated DNA gyrase complexes (PDB ID: 2XCS)[1] with the previously docked NBTI binding poses containing unsubstituted phenyl (**2**), i.e., *p*-halogenated phenyl RHSs (**3-6**) were conducted in duration of 500 ns. The stability, dynamics profile, and compactness of the simulated systems in first instance was assessed by inspecting the resulting MD trajectories in terms of analysing the plotted root-mean-square deviations (RMSD) and radius of gyration (*Rg*) for each system separately (Supplementary Fig. 2).

Taking into account the complexity of the studied systems as well as to obtain a clearer insight into their overall stability, RMSD plots over entire simulation time were calculated for each entity separately (e.g., backbone protein, DNA, and NBTI ligands; Supplementary Fig. 2). As depicted in Supplementary Fig. 2a, one can perceive well-equilibrated DNA gyrase systems (apo and ligated forms) as demonstrated by their calculated backbone protein RMSD values with no significant deviations (~2.01-2.78 Å). The apo form itself is typified by a good overall stability (~2.27-2.47 Å) that increases by complexation with our studied NBTI ligands, which is also evident by stabilization of the DNA (Supplementary Fig. 2b) in ligated NBTI complexes (~1.1-1.74 Å; intercalation of NBTIs

methoxy-naphthiridine LHS between central DNA base pairs) relative to the apo form (a significant fluctuation of ~2.00-3.24 Å). Among studied NBTIs (Supplementary Fig. 2c), *p*-bromophenyl derivative (**5**) can be assigned as the most stable one (~1.3-1.5 Å) followed by a similar stability pattern (~0.5-1.25 Å) for *p*-fluorophenyl (**3**) and *p*-chlorophenyl (**4**) derivatives (Supplementary Figs. 2a-b), while highest RMSD values (~0.36-1.50 Å) are noticeable for *p*-iodophenyl derivative (**6**), however with no dramatic overall fluctuations of around 1.0 Å on average.

These findings are congruent with the compactness assessments for apo and NBTIs ligated gyrase systems as depicted by the *Rg* plots (Supplementary Fig. 2d); although, one can observe similar *Rg* deviations of ~0.5-0.6 Å between all investigated systems, the system containing NBTI ligand with *p*-bromophenyl RHS (**5**) is the most compact one (~38.08-37.68 Å) followed by the complexes containing NBTIs with *p*-iodophenyl (**6**) and *p*-chlorophenyl (**4**) RHS (~38.23-37.73 Å and ~38.31-37.75 Å), while highest *Rg* fluctuations (~38.33-37.73 Å) could be attributed for the complex comprising an NBTI with *p*-fluorophenyl RHS (**3**) relative to the apo form (~38.1-37.56 Å). Such behaviour was expected to a certain extent, particularly in the case of (**3**), since despite the fluorine's high electronegativity as well as lack of polarizability, it is not involved in halogen bonding, and thus forms significantly weaker interactions compared to chloride, bromide, and iodine containing compounds, as previously hypothesized (Supplementary Fig. 1 and Supplementary Table 2)[2,3].

a

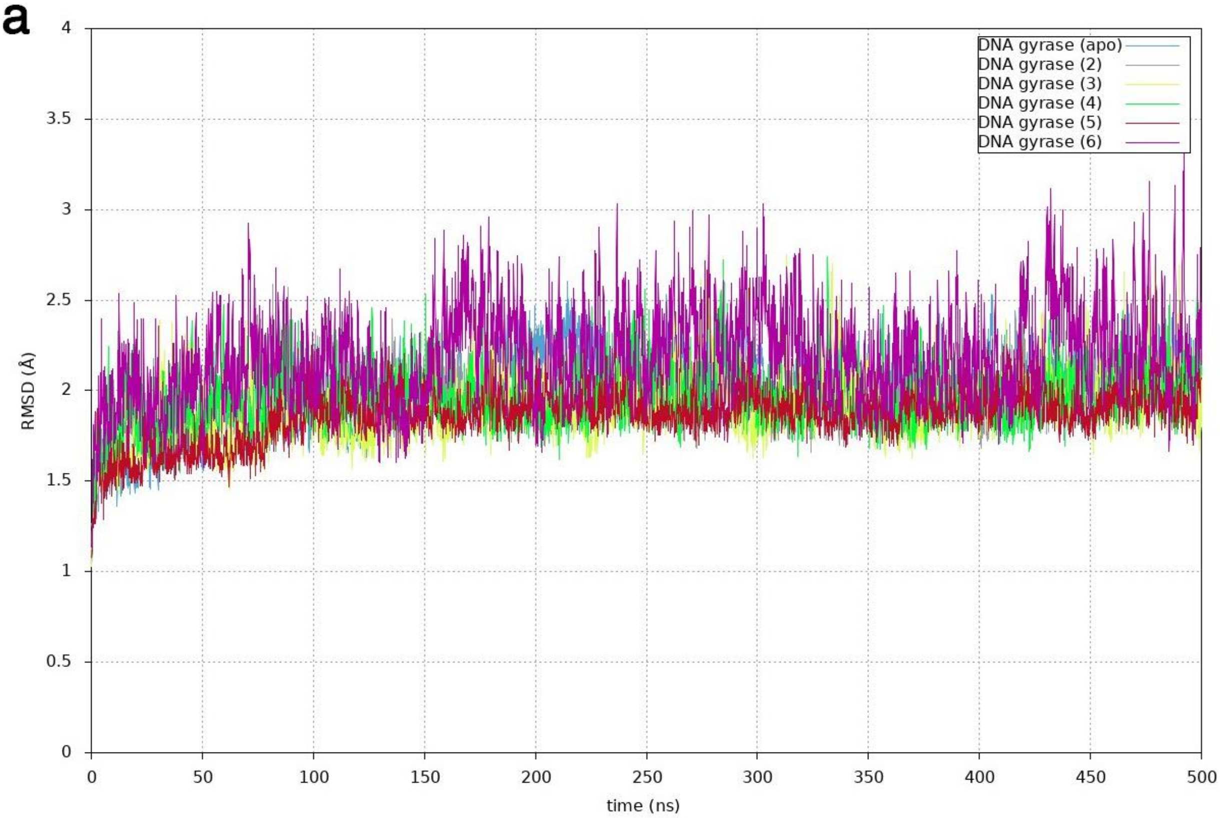

DNA gyrase (apo)
DNA gyrase (2)
DNA gyrase (3)
DNA gyrase (4)
DNA gyrase (5)
DNA gyrase (6)
RMSD (Å)
time (ns)
0
0.5
1
1.5
2
2.5
3
3.5
4
50
100
150
200
250
300
350
400
450
500

b

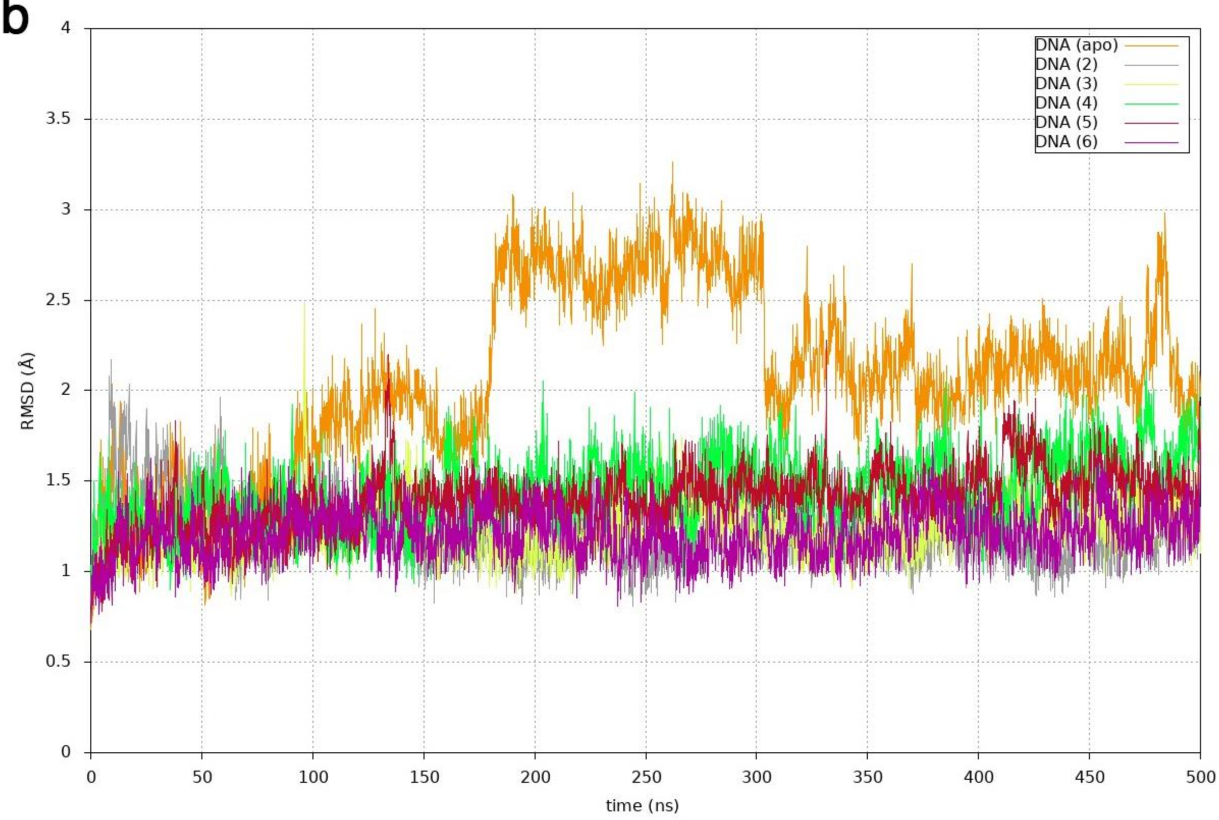

DNA (apo)
DNA (2)
DNA (3)
DNA (4)
DNA (5)
DNA (6)
RMSD (Å)
time (ns)
0
0.5
1
1.5
2
2.5
3
3.5
4
50
100
150
200
250
300
350
400
450
500

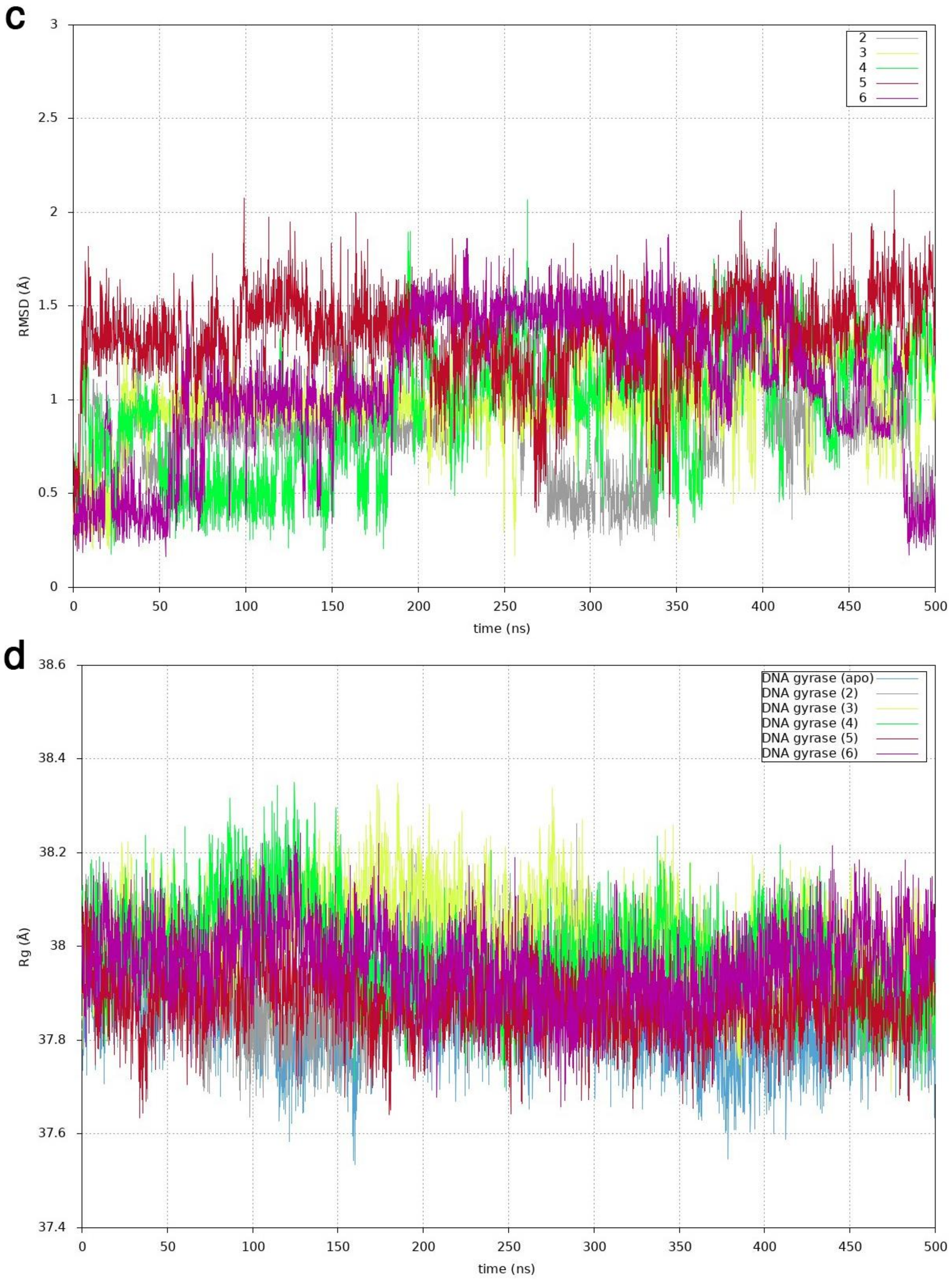

Supplementary Figure 2: Root-mean-square deviation (RMSD [Å]) and Radius of gyration (*Rg* [Å]) plots of 500 ns MD-simulated *S. aureus apo* (ligand-free) and DNA gyrase complexes (PDB ID: 2XCS)[1] with the compounds (2-6). a backbone DNA gyrase protein RMSD; **b** DNA RMSD; **c** compounds **2-6** RMSD; **d** *Rg* plots. RMSD and *Rg* curves are coloured as follows: apo form (inset: light-blue), (**2**)-DNA gyrase complex (inset: gray), (**3**)-DNA gyrase complex (inset: yellow), (**4**)-DNA

gyrase complex (inset: green), (**5**)-DNA gyrase complex (inset: dark-red), (**6**)-DNA gyrase complex (inset: magenta), bacterial DNA (inset: orange).

Supplementary Table 3. Average interatomic X···O distances [Å] and Θ_1 [C-X···O], i.e., Θ_2 [X···O=C] angles reflecting the halogen bonding propensities of compounds 3-6 to the backbone carbonyl oxygen of Ala68 from both GyrA subunits (PDB ID: 2XCS)[1] calculated from their resulting MD trajectories.

Comp.	d_1 [Å]’	d_2 [Å]’’	Θ_1[C-X···O]’	Θ_1[C-X···O]’’	Θ_2[X···O=C]’	Θ_2[X···O=C]’’
**3**	3.45	3.82	147.20°	127.39°	125.80°	110.98°
**4**	3.69	3.74	132.31°	150.24°	117.33°	118.25°
**5**	3.99	3.39	150.48°	129.81°	123.82°	118.27°
**6**	3.75	3.47	140.24°	131.09°	123.10°	121.09°

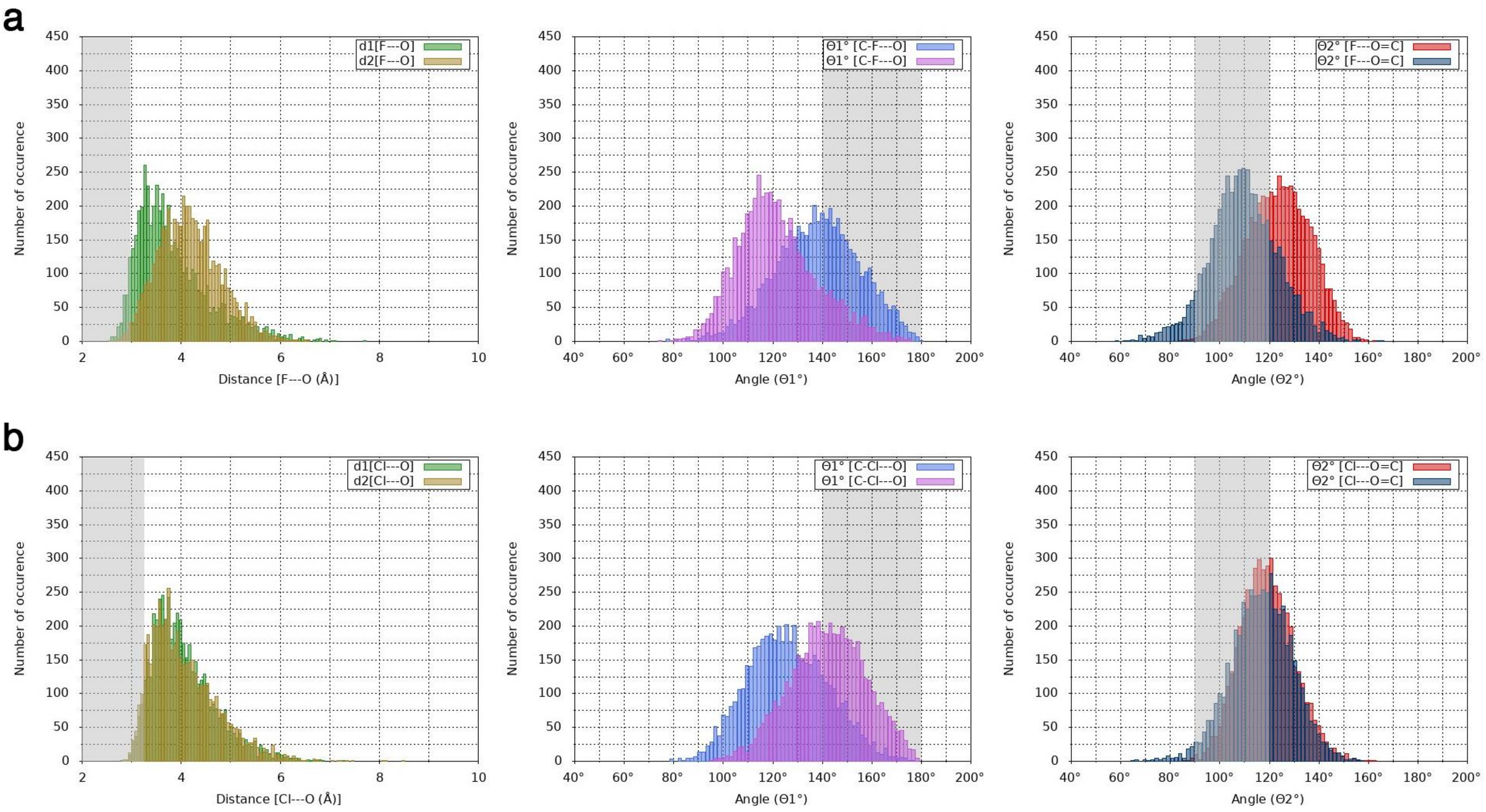
a
d1[F---O]
d2[F---O]
Number of occurence
Distance [F---O (Å)]
Θ1° [C-F---O]
Θ1° [C-F---O]
Angle (Θ1°)
Θ2° [F---O=C]
Θ2° [F---O=C]
Angle (Θ2°)
b
d1[Cl---O]
d2[Cl---O]
Distance [Cl---O (Å)]
Θ1° [C-Cl---O]
Θ1° [C-Cl---O]
Θ2° [Cl---O=C]
Θ2° [Cl---O=C]

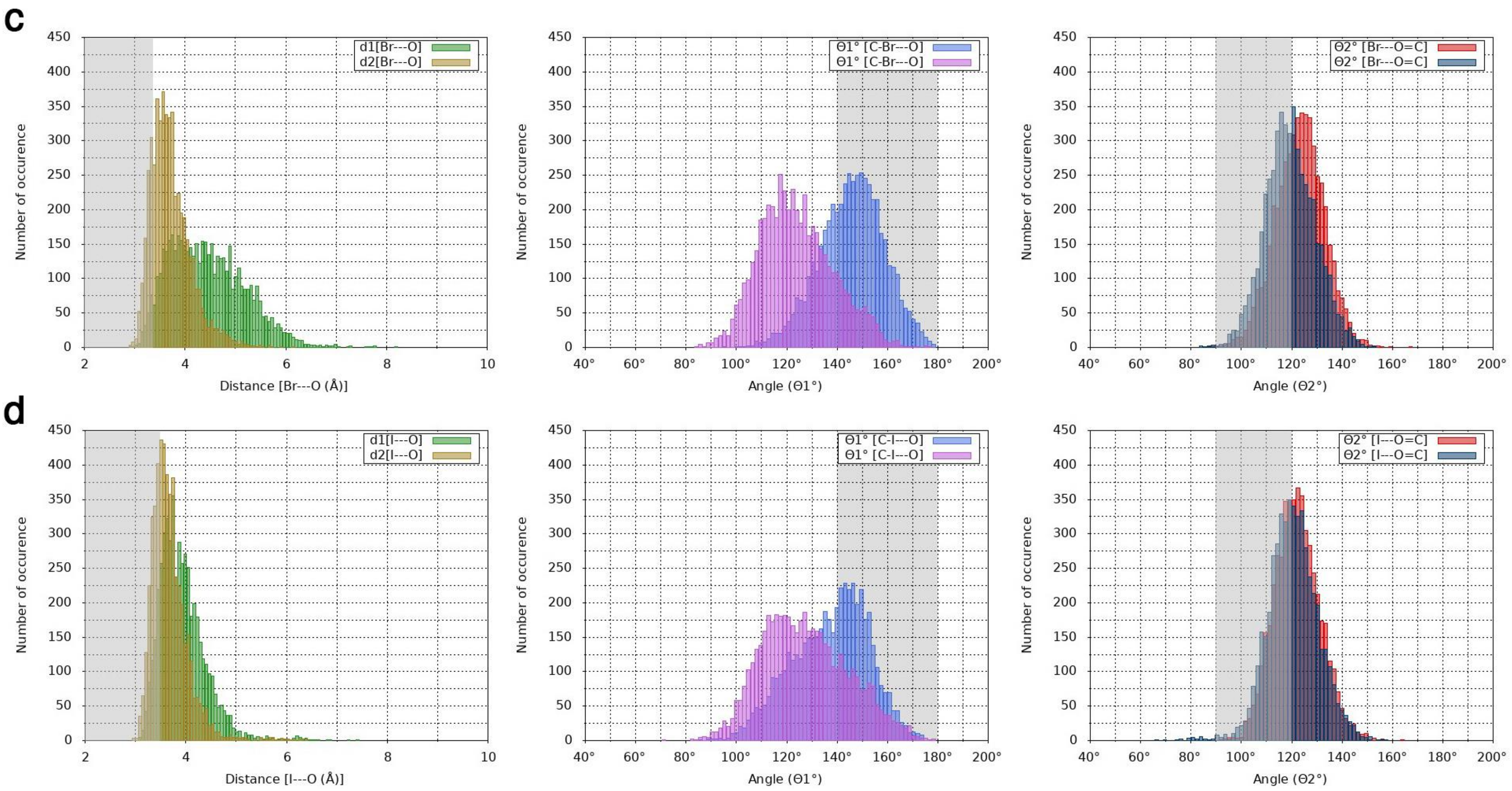

Supplementary Figure 3: X···O distances [Å] and Θ_1 [C-X···O], i.e., Θ_2 [X···O=C] angles distribution plots accounting for the halogen bonding propensity between *p*-substituted halogens of investigated NBTIs (3-6) and the backbone carbonyl oxygen of GyrA Ala68 residues (PDB ID: 2XCS)[1] calculated over the entire MD simulations time of 500 ns. a compound **3**; **b** compound **4**; **c** compound **5**; **d** compound **6**. Ala68' and Ala68'' X···O distances are represented as green and olive bars, Ala68' and Ala68'' Θ_1 [C-X···O] angles as light-blue and violet bars**,** while Ala68' and Ala68'' Θ_2 [X···O=C] angles as dark-blue and red bars, respectively. The gray shaded areas represent the allowed halogen bonding interatomic distances (F···O < 2.99 Å, Cl···O < 3.27 Å, Br···O < 3.37 Å, I···O < 3.50 Å) and angles (140° ≤ Θ_1 [C-X···O] ≤ 180° and 90° ≤ Θ_2 [X···O=C] ~ 120°)[4,5].

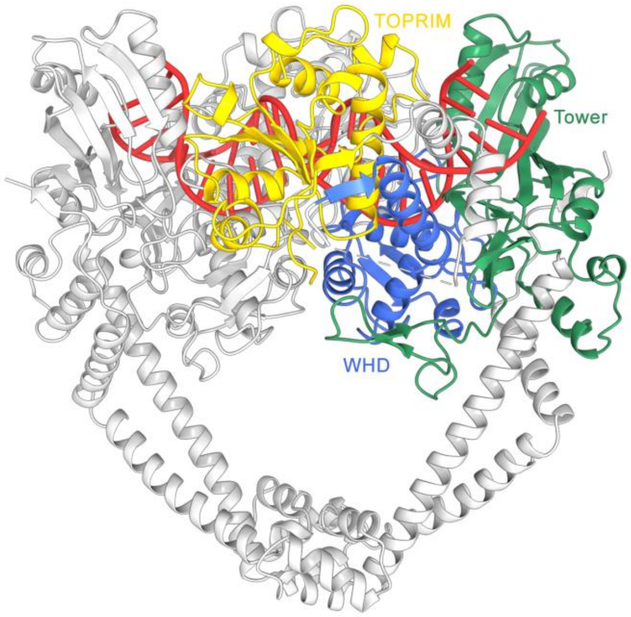

Supplementary Figure 4: Representation of TOPRIM, WHD and Tower domains. The TOPRIM domain of one GyrB subunit is depicted in yellow and the WHD and tower domains of one GyrA subunit are depicted in blue and green, respectively. Other subunits and second GyrA and GyrB subunits are depicted in grey.[1]

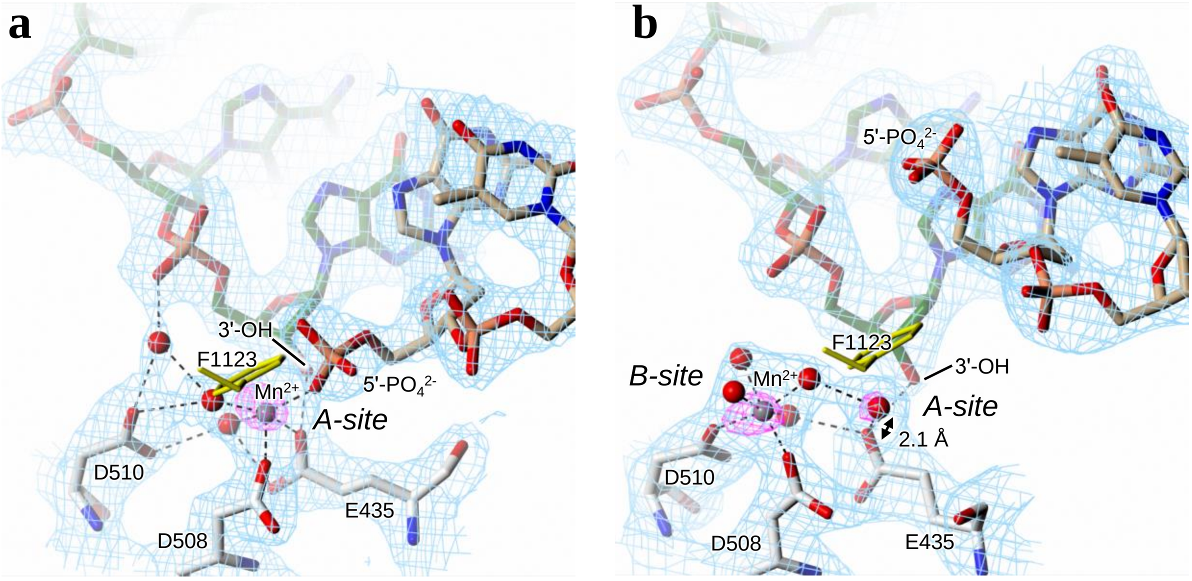

Supplementary Figure 5: Crystallographic evidence for the occupancies of the two metal sites. Detail of the catalytic sites in the A-configuration, **a** and B-configuration, **b (**PDB ID: 6Z1A**)**. Key residues in the vicinity of the active site are shown with white carbons, with the exception of the mutated catalytic residue, Phe1123, where the side-chain is shown with yellow carbons and thinner bonds (in the foreground relative to the rest of the figure). For the DNA fragment upstream of the break, the carbons are in green (in the background) and for the downstream fragment, the carbons are pale brown (in the foreground). Also shown in blue mesh is the final $2m\mathrm{F}_{obs}$-$D\mathrm{F}_{calc}$ electron density for the residues shown (with the exception of that for Phe1123; at 2.3 Å resolution and contoured at 1.1 σ), and the locations of the metal ions (grey spheres) are highlighted by peaks in an anomalous difference Fourier map shown in magenta mesh (contoured at 4.0 σ). The latter shows evidence for

partial occupancy of the A-site by metal in the B-configuration, although this was modelled as a fully occupied water molecule.

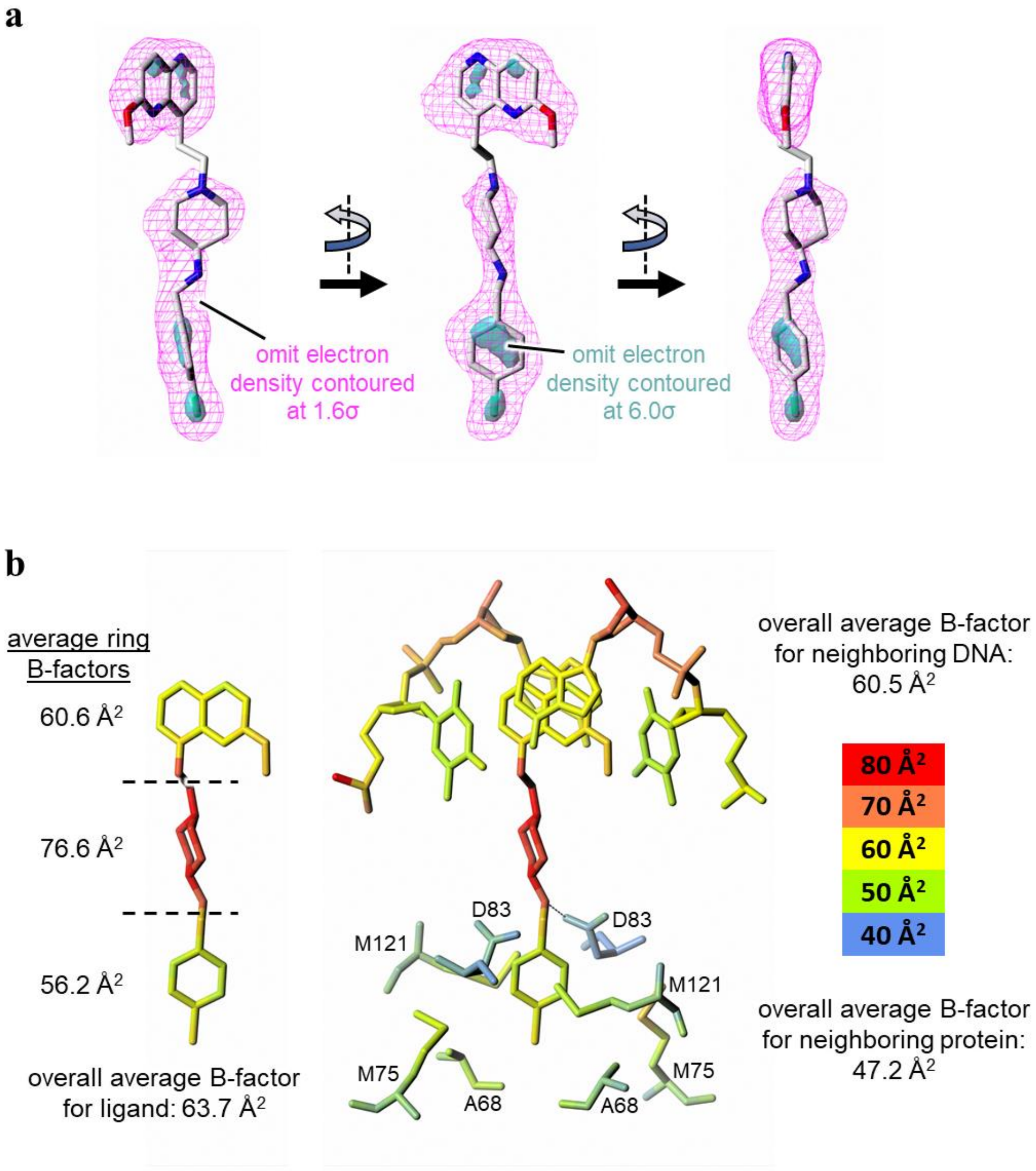

Supplementary Figure 6: Crystallographic evidence supporting the placement, orientation and conformation of compound 4 in the complex with DNA gyrase and DNA (PDB ID: 6Z1A). **a** Omit mFobs-DFcalc difference electron density for the bound ligand calculated at 2.3-Å resolution and contoured at 1.6 σ (magenta mesh) and at 6.0 σ (cyan semi-transparent surface). Note that the latter highlights the confidence in the placement of the chlorine atom. The three orientations are chosen to emphasize the how the density associated with each ring is flattened, which aids in the assignment of their orientations. **b** The refined ligand has a overall B-factor that is somewhat elevated relative to the surrounding protein, but is comparable to that of the DNA. A breakdown shows there is a noticeably higher value for the aminopiperidine linker, which is in line with the lack of interactions with protein or DNA for this region of the ligand. For clarity, the ligand is shown in isolation (left) and in the context of the complex (right). In both panels, atoms are coloured according to their B-factors by interpolation between the colours shown in the key. So, for example an atom with a B-factor of 55 $Å^2$ would have a colour intermediate between green and yellow.

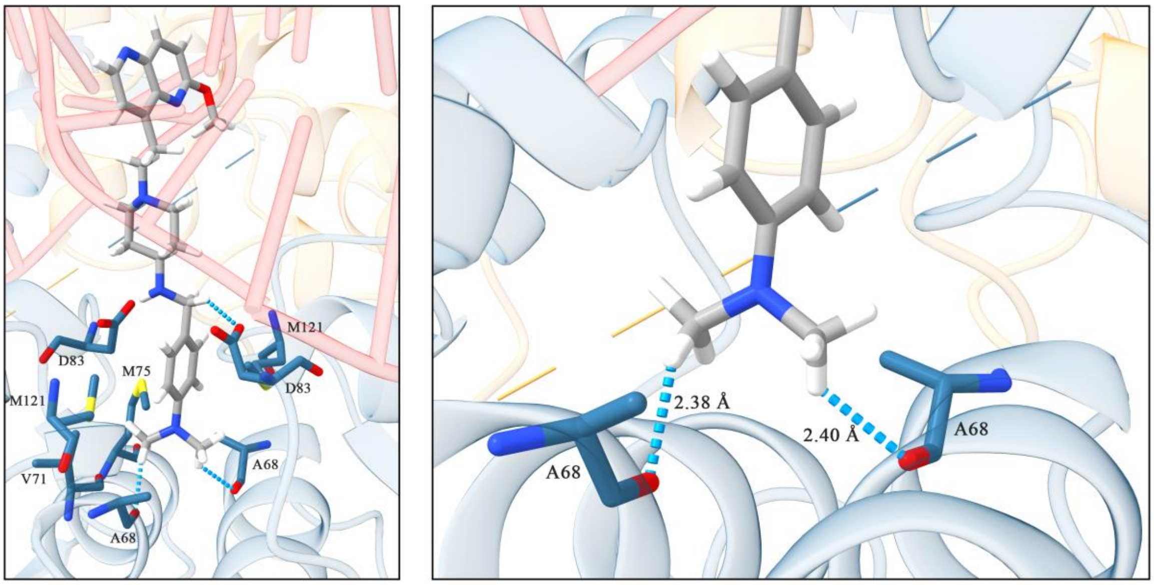

Supplementary Figure 7: Predicted binding modes of compound 9. Molecular docking results representing compound **9** (*p*-dimethylamino phenyl; sticks representation colour-coded by element) within the *S. aureus* DNA gyrase NBTI-binding site of our crystal structure (PDB ID: 6Z1A), revealing key intermolecular interactions. Hydrogen bonds are represented as blue dots, whereby, each methyl group forms pseudohydrogen bond with Ala68. GyrA, GyrB, and DNA are represented as cartoons in blue, yellow, and red, respectively. The amino acid residues are represented as sticks colour-coded by element.

Supplementary Table 4. logP and logD of compounds 2-9 calculated with MarvinSketch 20.17.[6]

Cmpd	logD	logP
**2**	1,17	3.33
**3**	1,47	3.48
**4**	1,89	3.94
**5**	2,02	4.10
**6**	2,18	4.26
**7**	0,44	2.57
**8**	0,19	2.18
**9**	0,18	3.44

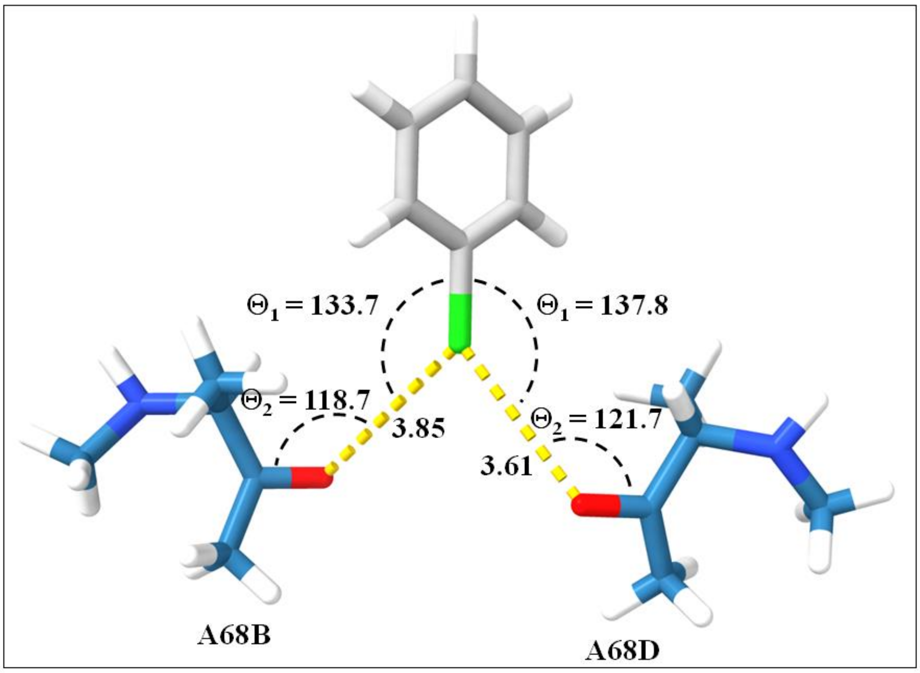

Supplementary Figure 8 A truncated system representing the complex between compound 4 and gyrase used for QM calculations of the interaction energies. Compound **4** (taken from PDB ID: 6Z1A) was truncated solely on the RHS part (chlorophenyl moiety; sticks representation colour-coded by element) that establishes direct, bifurcated halogen-bonding interactions (yellow dots) with the backbone carbonyl oxygens of Ala68 residues (sticks representation colour-coded by element) of each GyrA subunit (truncated and patched with methyl groups).

Supplementary Table 5. Interaction energies between the compound 4 (chlorophenyl RHS), 2 (unsubstituted phenyl RHS) and Ala68 residues obtained by QM calculations. The interaction energies, i.e., dispersion components on HF level were calculated by $\Delta E = E(\text{lig.}-\text{AlaBD complex}) - E(\text{lig.}) - E(\text{AlaBD})$.

Comp.	*Δ*Eint [Ala68B] (kcal mol^{-1})	*Δ*Eint [Ala68D] (kcal mol^{-1})	*Δ*Eint [Ala68BD] (kcal mol^{-1})	*Δ*Edisp_HF [Ala68BD] (kcal mol^{-1})
**4**	-2.00	-2.02	-4.23	1.38
**2**	-1.59	-1.60	-3.18	-0.58

Synthesis

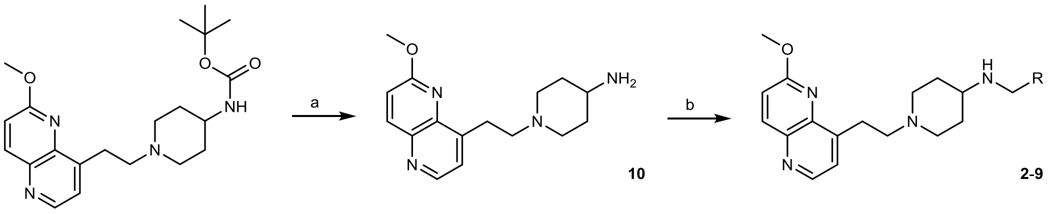

Supplementary Figure 9: Synthesis of final compounds 2-9. Reagents and conditions: a) 1M HCl in CH_3COOH, 30 min, RT; b) RCHO, MeOH, CH_3COOH, 2 h, then $NaCNBH_3$, 0 °C, 10 min, then RT.

1-(2-(6-methoxy-1,5-naphthyridin-4-yl)ethyl)piperidin-4-amine (10)

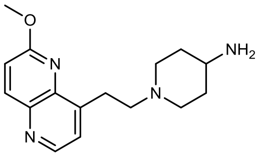

Tert-butyl 1-(2-(6-methoxy-1,5-naphthyridin-4-yl)ethyl)piperidin-4-ylcarbamate (0.63 g, 1.63 mmol, 1 equiv) was dissolved in 12.5 ml of 1 M HCl in acetic acid and stirred for 30 min at room temperature. Solvent was evaporated, the residue dissolved in 30 mL of saturated $NaHCO_{3(aq)}$ and pH adjusted to 12 with 1 M NaOH. Water layer was washed with DCM (3 × 30 mL), combined organic layers dried over Na_2SO_4 and concentrated in vacuum to afford compound **10** as dark-brown viscous liquid (0.33 g, 71%).
TLC (DCM:MeOH 4:1 + 1% Et_3N v/v): R_f = 0.10; ^{1}H NMR (400 MHz, 295 K, $CDCl_3$): δ 1.41–1.51 (m, 2H), 1.88 (d, J = 11.9 Hz, 2H), 2.20 (t, J = 11.0 Hz, 2H), 2.66–2.78 (m, 1H), 2.73–2.84 (m, 2H), 3.05 (d, J = 11.5 Hz, 2H), 3.30–3.46 (m, 2H), 4.08 (s, 3H), 7.11 (d, J = 9.0 Hz, 1H), 7.41 (d, J = 4.5 Hz, 1H), 8.19 (d, J = 9.0 Hz, 1H), 8.66 (d, J = 4.5 Hz, 1H) ppm; ^{13}C NMR (100 MHz, 295 K, $CDCl_3$): δ 28.46, 35.83, 52.37, 53.72, 58.37, 77.23, 116.31, 124.24, 140.33, 140.97, 141.48, 146.64, 147.70, 161.43 ppm; IR (ATR): 3271, 2941, 2811, 1614, 1590, 1503, 1489, 1438, 1398, 1334, 1125, 1079, 1018, 932, 853, 807, 751, 713, 615, 584, 536 cm^{-1}; HRMS (*m/z*): [M + H]$^+$ calcd. for $C_{16}H_{22}N_4O_{14}$ 287.1872; found 287.1875. *signal is overlapping with residual solvent peak.

General procedure for reductive amination: To a solution of amine **10** (1 equiv) in dry methanol the appropriate aldehyde (1.05-1.3 equiv) was added, together with catalytic amount of acetic acid. The reaction mixture was stirred for 2 h at room temperature under inert atmosphere. Next, the reaction mixture was cooled to 0 °C and $NaCNBH_3$ (3-5 equiv) dissolved in small amount of dry methanol was added drop-wise. After stirring overnight at room temperature under inert atmosphere, the solvent was evaporated in vacuum and the residue diluted in ethyl acetate. Organic phase was washed with saturated Na_2CO_3 three times, dried over Na_2SO_4, filtered and the solvent evaporated under reduced pressure. The residue was purified by flash column chromatography to afford appropriate substituted product.

***N*-benzyl-1-(2-(6-methoxy-1,5-naphthyridin-4-yl)ethyl)piperidin-4-amine (2)**

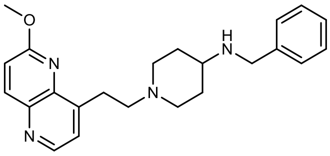

According to the general procedure for reductive amination, compound **10** (0.2 g, 0.698 mmol, 1 equiv) was used together with benzaldehyde (0.092 mL, 0.908 mmol, 1.3 equiv). The residue was

purified by flash column chromatography using $CHCl_3$/MeOH (9:1) to afford compound **2** (0.064 g, 24%) as yellow solid.
mp: 47.7–50.1 °C; TLC (DCM:MeOH 4:1 v/v and $CHCl_3$:MeOH 9:1 v/v): R_f = 0.28 and 0.14; ^{1}H NMR (400 MHz, 295 K, $CDCl_3$): δ 1.48–1.58 (m, 2H), 1.98–2.01 (m, 2H), 2.19–2.25 (m, 2H), 2.57–2.62 (m, 1H), 2.80–2.84 (m, 2H), 3.07–3.10 (m, 2H), 3.39–3.43 (m, 2H), 3.86 (s, 2H), 4.10 (s, 3H), 7.13 (d, *J* = 8.8 Hz, 1H), 7.28–7.30 (m, 1H)*, 7.36 (d, *J* = 4.4 Hz, 4H), 7.43 (d, *J* = 4.4 Hz, 1H), 8.21 (d, *J* = 9.2 Hz, 1H), 8.68 (d, *J* = 4.4 Hz, 1H) ppm; ^{13}C (100 MHz, 295 K, $CDCl_3$): δ 28.47, 32.80, 50.82, 52.35, 53.72, 54.09, 58.46, 77.24*, 116.27, 124.24, 126.90, 128.06, 128.44, 140.34, 140.75, 140.99, 141.49, 146.75, 147.71, 161.40 ppm; IR (ATR): 2942, 2817, 1611, 1590, 1489, 1452, 1397, 1361, 1334, 1301, 1256, 1183, 1107, 1075, 1018, 987, 849, 808, 747, 697, 649, 614, 537 cm^{-1}; HRMS (*m/z*) $[M + H]^+$ calcd. for $C_{23}H_{29}ON_4$, 377.2336; found 377.2334; HPLC purity: 91.9%.
*signal is overlapping with residual solvent peak.

***N*-(4-fluorobenzyl)-1-(2-(6-methoxy-1,5-naphthyridin-4-yl)ethyl)piperidin-4-amine (3)**

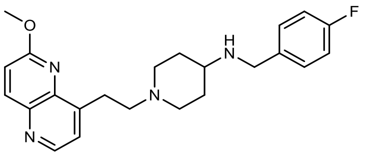

According to the general procedure for reductive amination, compound **10** (0.233 g, 0.814 mmol, 1 equiv) was used together with 4-fluorobenzaldehyde (0.09 mL, 0.853 mmol, 1.05 equiv). The residue was purified by flash column chromatography using $CHCl_3$/MeOH (9:1 + 1% Et_3N) to afford compound **3** (0.233 g, 69%) as light brown solid.
mp: 55–60 °C; TLC (DCM:MeOH 4:1 + 1 % Et_3N v/v): R_f = 0.2; ^{1}H NMR (400 MHz, 295 K, $CDCl_3$): δ 1.46–1.55 (m, 2H)*, 1.97 (d, *J* = 11.6 Hz, 2H), 2.20 (t, *J* = 10.9 Hz, 2H), 2.52–2.61 (m, 1H), 2.77–2.85 (m, 2H), 3.07 (d, *J* = 11.8 Hz, 2H), 3.36–3.44 (m, 2H), 3.82 (s, 2H), 4.10 (s, 3H), 7.01–7.06 (m, 2H), 7.13 (d, *J* = 9.0 Hz, 1H), 7.30–7.35 (m, 2H), 7.43 (d, *J* = 4.5 Hz, 1H), 8.21 (d, *J* = 9.0 Hz, 1H), 8.68 (d, *J* = 4.5 Hz, 1H) ppm; ^{13}C NMR (100 MHz, 295 K, $CDCl_3$): δ 28.43, 32.71, 50.07, 52.32, 53.71, 58.41, 77.24*, 115.20 (d, *J* = 21.2 Hz), 116.29, 124.25, 129.54 (d, *J* = 8.0 Hz), 136.41, 140.35, 140.97, 141.50, 146.67, 147.71, 161.42, 161.88 (d, *J* = 244.6 Hz) ppm; IR (ATR): 3035, 2935, 2811, 1611, 1592, 1505, 1489, 1468, 1441, 1398, 1369, 1334, 1299, 1263, 1215, 1106, 1077, 1017, 932, 850, 812, 790, 751, 712, 696, 647, 607, 584, 564 cm^{-1}; HRMS (*m/z*) $[M + H]^+$ calcd. for $C_{23}H_{27}FN_4O$, 395.2242; found 395.2238; HPLC purity: 94.9%.
*signal is overlaping with residual solvent peak.

***N*-(4-chlorobenzyl)-1-(2-(6-methoxy-1,5-naphthyridin-4-yl)ethyl)piperidin-4-amine (4)**

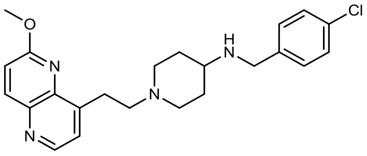

According to the general procedure for reductive amination, compound **10** (0.2 g, 0.698 mmol, 1 equiv) was used together with 4-chlorobenzaldehyde (0.128 g, 0.908 mmol, 1.3 equiv). The residue was purified by flash column chromatography using $CHCl_3$/MeOH (9:1) to afford compound **4** (0.220 g, 77%) as orange-brown solid.
mp: 67.6–68.4 °C; TLC (DCM:MeOH 4:1 and $CHCl_3$:MeOH 4:1) R_f = 0.44 and 0.32; ^{1}H NMR (400 MHz, 295 K, $CDCl_3$): δ 1.43–1.53 (m, 2H), 1.93–1.96 (m, 2H), 2.15–2.20 (m, 2H), 2.50–2.55 (m, 1H), 2.77–2.81 (m, 2H), 3.03–3.06 (m, 2H), 3.36–3.40 (m, 2H), 3.80 (s, 2H), 4.07 (s, 3H), 7.11 (d, *J* = 8.8 Hz, 1H), 7.26–7.31 (m, 4H)*, 7.41 (d, *J* = 4.4 Hz, 1H), 8.18 (d, *J* = 8.8 Hz, 1H), 8.65 (d, *J* = 4.4 Hz, 1H) ppm; ^{13}C (100 MHz, 295 K, $CDCl_3$): δ 28.44, 32.69, 50.06, 52.29, 53.71, 54.04, 58.40, 77.25*, 116.30, 124.74, 128.53, 129. 39, 132.56, 139.19, 140.32, 140.96, 141.47, 146.65, 147.69, 161.42 ppm;

IR (ATR): 3223, 2945, 2827, 1490, 1262, 1092, 857, 810, 757, 641, 603 cm^{-1}; HRMS (*m/z*) $[M + H]^+$ calcd. for $C_{23}H_{28}ON_4Cl$, 411.1946; found 411.1930; HPLC purity: 96.69%.
*signal is overlapping with residual solvent peak.

_N_-(4-bromobenzyl)-1-(2-(6-methoxy-1,5-naphthyridin-4-yl)ethyl)piperidin-4-amine (5)

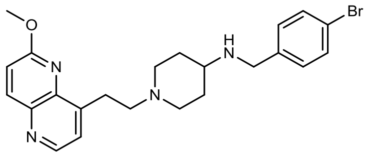

According to the general procedure for reductive amination, compound **10** (0.2 g, 0.698 mmol, 1 equiv) was used together with 4-bromobenzaldehyde (0.168 g, 0.908 mmol, 1.3 equiv). The residue was purified by flash column chromatography using $CHCl_3$/MeOH (9:1) to afford compound **5** (0.293 g, 92%) as yellow-orange solid.
mp = 74.7–75.8 °C; TLC (DCM:MeOH 4:1 and $CHCl_3$:MeOH 9:1) R_f = 0.55 and 0.29; ^{1}H NMR (400 MHz, 295 K,$CDCl_3$): δ 1.43–1.52 (m, 2H), 1.93–1.96 (m, 2H), 2.15–2.20 (m, 2H), 2.50–2.55 (m, 1H), 2.77–2.81 (m, 2H), 3.03–3.06 (m, 2H), 3.36–3.40 (m, 2H), 3.79 (s, 2H), 4.07 (s, 3H), 7.11 (d, *J* = 8.8 Hz, 1H), 7.22 (d, *J* = 6.32, 2H), 7.40–7.45 (m, 3H), 8.18 (d, *J* = 8.8 Hz, 1H), 8.65 (d, *J* = 4.4 Hz, 1H) ppm; ^{13}C (100 MHz, 295 K, $CDCl_3$): δ 28.44, 32.69, 50.10, 52.29, 53.71, 54.03, 58.40, 77.25*, 116.30, 120.63, 124.74, 129.77, 131.48, 139.72, 140.32, 140.97, 141.46, 146.66, 147.68, 161.42 ppm; IR (ATR): 3224, 2942, 2826, 1589, 1489, 1402, 1335, 1263, 1092, 1006, 856, 810, 757, 656, 634, 598, 545, 521 cm^{-1}; HRMS (*m/z*) $[M + H]^+$ calcd. for $C_{23}H_{28}ON_4Br$, 455.1441; found 455.1424; HPLC purity: 97.53%.
*signal is overlapping with residual solvent peak.

_N_-(4-iodobenzyl)-1-(2-(6-methoxy-1,5-naphthyridin-4-yl)ethyl)piperidin-4-amine (6)

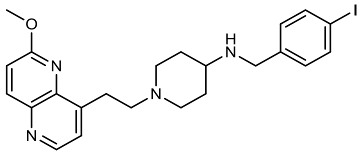

According to the general procedure for reductive amination, compound **10** (0.2 g, 0.698 mmol, 1 equiv) was used together with 4-iodobenzaldehyde (0.210 g, 0.908 mmol, 1.3 equiv). The residue was purified by flash column chromatography using $CHCl_3$/MeOH (9:1) to afford compound **6** (0.285 g, 81%) as brown-yellow solid.
mp = 80.8–82.7 °C; TLC (DCM:MeOH 4:1 and $CHCl_3$:MeOH 9:1) R_f = 0.4 and 0.17; ^{1}H NMR (400 MHz, 295 K, $CDCl_3$): δ 1.43–1.52 (m, 2H)*, 1.92–1.95 (m, 2H), 2.14–2.20 (m, 2H), 2.50–2.55 (m, 1H), 2.77–2.81 (m, 2H), 3.03–3.06 (m, 2H), 3.36–3.40 (m, 2H), 3.78 (s, 2H), 4.07 (s, 3H), 7.08–7.12 (m, 3H), 7.41 (d, *J* = 4.4 Hz, 1H), 7.41 (d, *J* = 4.4 Hz, 1H), 7.64 (d, *J* = 8.4 Hz, 2H), 8.18 (d, *J* = 8.8 Hz, 1H), 8.66 (d, *J* = 4.4 Hz, 1H) ppm; ^{13}C (100 MHz, 295 K, DMSO-d6): δ 28.42, 32.66, 50.17, 52.27, 53.72, 58.38, 77.24*, 92.10, 116.30, 124.26, 130.07, 137.46, 140.36, 140.44, 140.96, 141.50, 146.60, 147.72, 161.43 ppm; IR (ATR): 2921, 2791, 1612, 1586, 1502, 1487, 1398, 1333, 1260, 1142, 1120, 1095, 1076, 1007, 930, 886, 855, 800, 737, 706, 627, 583, 538 cm^{-1}; HRMS (*m/z*) $[M + H]^+$ calcd. for $C_{23}H_{28}ON_4$, 503.1302; found 503.1298; HPLC purity: 99.62%.
*signal is overlapping with residual solvent peak.

(4-((1-(2-(6-methoxy-1,5-naphthyridin-4-yl)ethyl)piperidin-4-ylamino)methyl)phenyl)methanol (7)

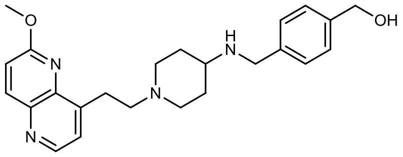

According to the general procedure for reductive amination, compound **10** (0.2 g, 0.698 mmol, 1 equiv) was used together with 4-(hydroxymethyl)benzaldehyde (0.123 g, 0.908 mmol, 1.3 equiv). The residue was purified by flash column chromatography using $CHCl_3$/MeOH (9:1) to afford compound **7** (0.122 g, 43%) as light brown solid.
mp = 73.4–76.7 °C; TLC (DCM:MeOH 4:1 and $CHCl_3$:MeOH 9:1) R_f = 0.07 and 0.03; ^{1}H NMR (400 MHz, 295 K, $CDCl_3$): δ 1.48–1.57 (m, 2H), 1.97–2.00 (m, 2H), 2.18–2.23 (m, 2H), 2.55–2.61 (m, 1H), 2.79–2.83 (m, 2H), 3.06–3.09 (m, 2H), 3.38–3.42 (m, 2H), 3.85 (s, 2H), 4.09 (s, 3H), 4.71 (s, 2H), 7.13 (d, *J* = 8.8 Hz, 1H), 7.35 (s, 4H), 7.43 (d, *J* = 4.8 Hz, 1H), 8.20 (d, *J* = 8.8 Hz, 1H), 8.67 (d, *J* = 4.4 Hz, 1H) ppm; ^{13}C (100 MHz, 295 K, $CDCl_3$): δ 28.39, 32.48, 50.39, 52.24, 53.67, 53.79, 58.35, 65.04,77.21*, 116.32, 124.25, 127.20, 128.33, 139.79, 140.32, 140.96, 141.46, 146.62, 147.66, 147.70, 161.43 ppm; IR (ATR): 2938, 2821, 1611, 15920, 1503, 1487, 1446, 1397, 1333, 1257, 1174, 1104, 1073, 1050, 1017, 990, 930, 849, 805, 753, 613, 585 cm^{-1}; HRMS (*m/z*) [M + H]$^+$ calcd. for $C_{24}H_{31}O_2N_4$, 407.2442; found 407.2440; HPLC purity: 98.50%.
*signal is overlapping with residual solvent peak.

4-((1-(2-(6-methoxy-1,5-naphthyridin-4-yl)ethyl)piperidin-4-ylamino)methyl)benzamide (8)

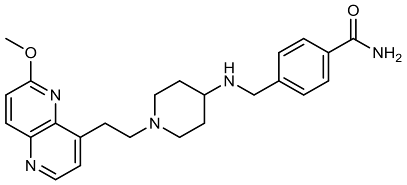

According to the general procedure for reductive amination, compound **10** (0.2 g, 0.698 mmol, 1 equiv) was used together with 4-formylbenzamide (0.135 g, 0.908 mmol, 1.3 equiv). The residue was purified by flash column chromatography using $CHCl_3$/MeOH (9:1) to afford compound **8** (0.033 g, 11%) as yellow solid.
mp = 116.1–122.1 °C; TLC (DCM:MeOH 4:1 and $CHCl_3$:MeOH 9:1) R_f = 0.06 and 0.02; ^{1}H NMR (400 MHz, 295 K, $CDCl_3$): δ 1.44–1.54 (m, 2H), 1.94–1.96 (m, 2H), 2.14–2.20 (m, 2H), 2.51–2.56 (m, 1H), 2.77–2.81 (m, 2H), 3.04–3.07 (m, 2H), 3.36–3.40 (m, 2H), 3.90 (s, 2H), 4.07 (s, 3H), 5.59–6.17 (m, 2H), 7.11 (d, *J* = 8.8 Hz, 1H), 7.41–7.44 (m, 3H), 7.78 (d, *J* = 8.4 Hz, 2H), 8.18 (d, *J* = 9.2 Hz, 1H), 8.66 (d, *J* = 4.4 Hz, 1H) ppm; ^{13}C (100 MHz, 295 K, DMSO): δ 27.49, 32.05, 49.33, 51.67, 53.27, 53.58, 57.85, 115.99, 124.46, 127.20, 127.41, 132.32, 140.24, 140.45, 140.81, 144.74, 146.15, 147.66, 160.80, 167.10 ppm; IR (ATR): 3288, 3148, 2941, 2838, 1681, 1614, 1591, 1567, 1495, 1450, 1398, 1336, 1289, 1264, 1134, 1105, 1083, 1017, 990, 869, 851, 807, 782, 757, 691, 657, 631, 602, 586, 547 cm^{-1}; HRMS (*m/z*) [M + H]$^+$ calcd. for $C_{24}H_{30}O_2N_5$, 420.2394; found 420.2391; HPLC purity: 99.25%.

***N*-(4-(dimethylamino)benzyl)-1-(2-(6-methoxy-1,5-naphthyridin-4-yl)ethyl)piperidin-4-amine (9)**

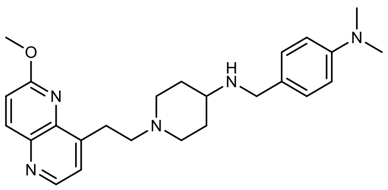

According to the general procedure for reductive amination, compound **10** (0.2 g, 0.698 mmol, 1 equiv) was used together with 4-(dimethylamino)benzaldehyde (0.135 g, 0.908 mmol, 1.3 equiv). The

residue was purified by flash column chromatography using $CHCl_3$/MeOH (9:1) to afford compound **9** (0.196 g, 67%) as yellow-orange solid.
mp = 89.4–91.6 °C; TLC (DCM:MeOH 4:1 and $CHCl_3$:MeOH 9:1) R_f = 0.07 and 0.05; ^{1}H NMR (400 MHz, 295 K, $CDCl_3$): δ 1.47–1.57 (m, 2H), 1.95–1.98 (m, 2H), 2.15–2.21 (m, 2H), 2.56–2.62 (m, 1H), 2.76–2.80 (m, 2H), 2.93 (s, 6H), 2.98–3.09 (m, 2H), 3.35–3.39 (m, 2H), 3.74 (s, 2H), 4.07 (s, 3H), 6.71 (d, *J* = 8.8 Hz, 2H), 7.11 (d, *J* = 8.8 Hz, 1H), 7.21 (d, *J* = 8.8 Hz, 2H), 7.41 (d, *J* = 4.4 Hz, 1H), 8.18 (d, *J* = 8.8 Hz, 1H), 8.66 (d, *J* = 4.4 Hz, 1H) ppm; ^{13}C (100 MHz, 295 K, $CDCl_3$): δ 28.43, 32.33, 40.74, 49.97, 52.26, 53.72, 58.39, 77.23*, 112.71, 116.26, 124.24, 129.19, 140.31, 140.96, 141.46, 146.71, 147.67, 149.87, 161.39 ppm; IR (ATR): 2920, 2802, 1611, 1593, 1523, 1488, 1397, 2335, 1262, 1217, 1191, 1166, 1146, 1104, 1063, 1018, 958, 934, 856, 837, 809, 755, 712, 696, 647, 586, 566 cm^{-1}; HRMS (*m/z*) $[M + H]^+$ calcd. for $C_{25}H_{34}ON_5$, 420.2758; found 420.2754; HPLC purity: 95.68%.
*signal is overlapping with residual solvent peak.

NMR spectra

N-benzyl-1-(2-(6-methoxy-1,5-naphthyridin-4-yl)ethyl)piperidin-4-amine (**2**)

^{1}H NMR (400 MHz, $CDCl_3$)

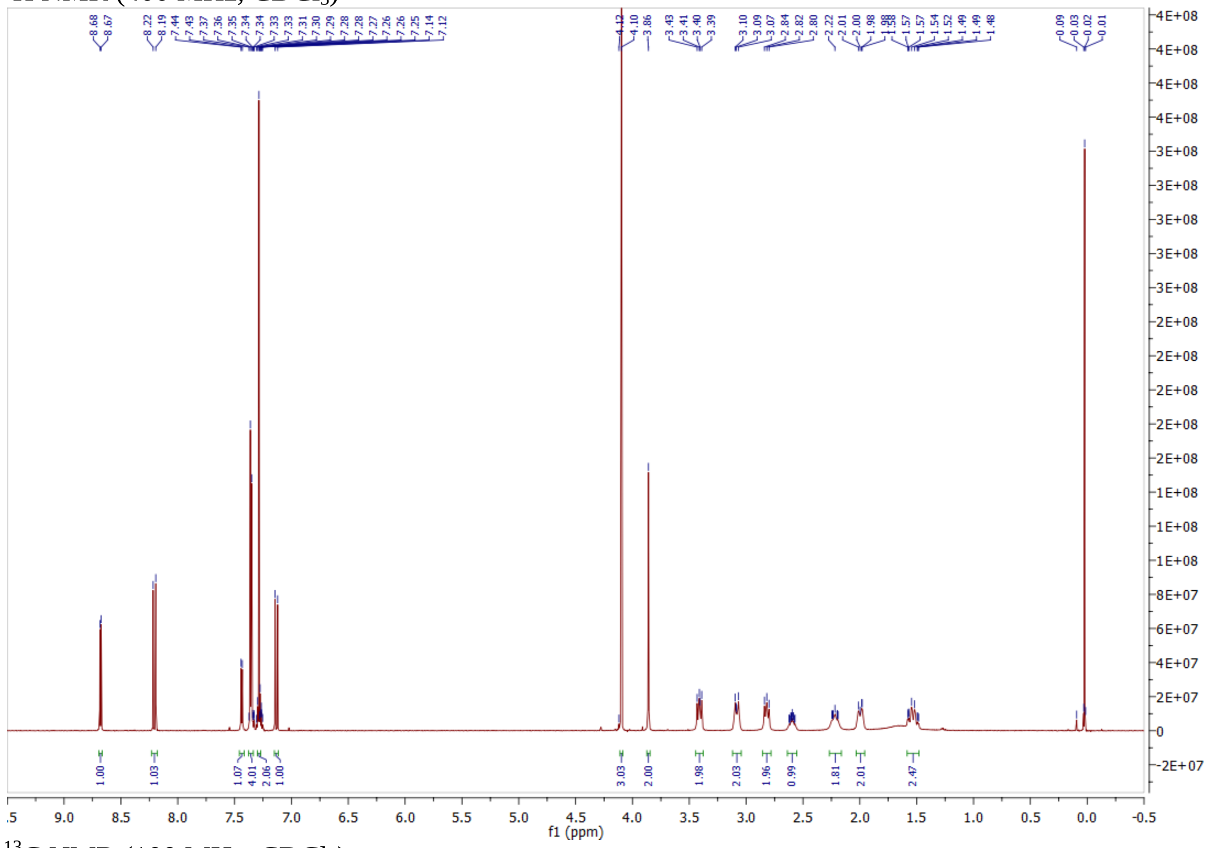

^{13}C NMR (100 MHz, $CDCl_3$)

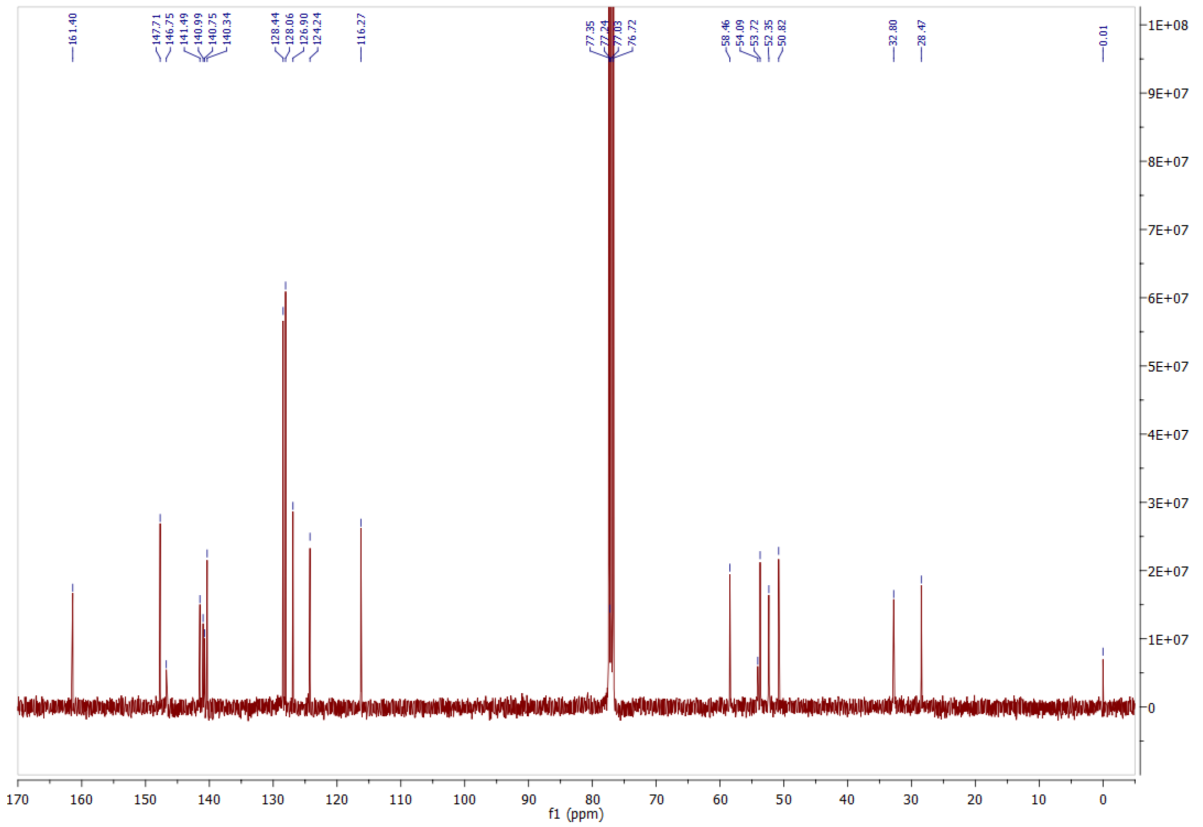

N-(4-fluorobenzyl)-1-(2-(6-methoxy-1,5-naphthyridin-4-yl)ethyl)piperidin-4-amine (**3**)

^{1}H NMR (400 MHz, $CDCl_3$)

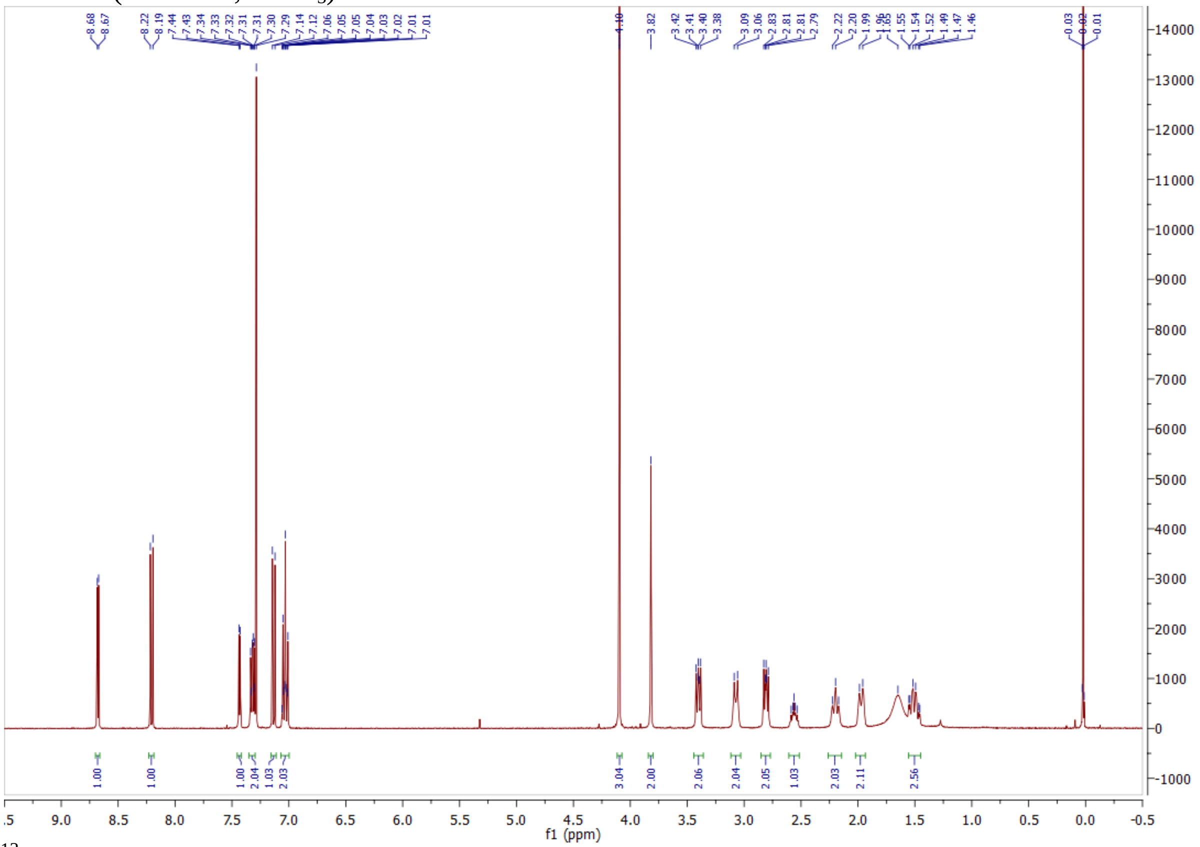

^{13}C NMR (100 MHz, $CDCl_3$)

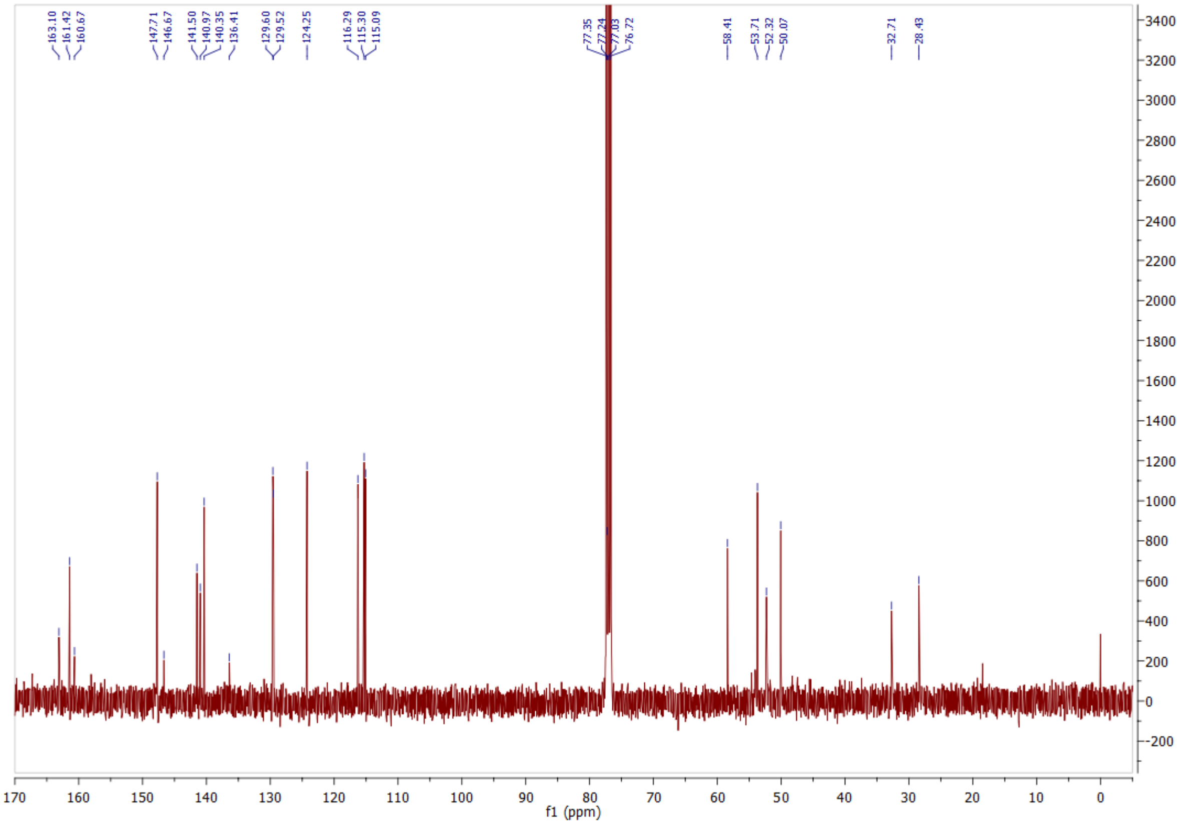

N-(4-chlorobenzyl)-1-(2-(6-methoxy-1,5-naphthyridin-4-yl)ethyl)piperidin-4-amine (**4**)

^{1}H NMR (400 MHz, $CDCl_3$)

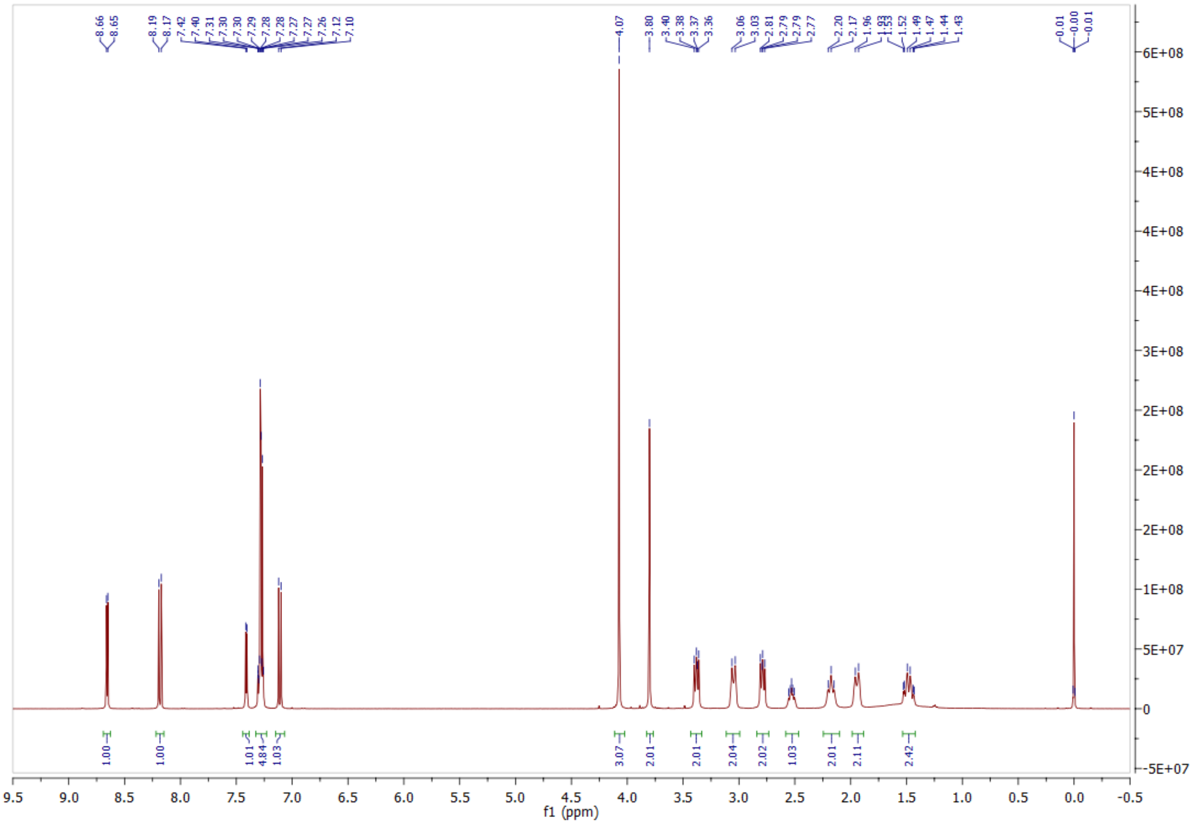

^{13}C NMR (100 MHz, $CDCl_3$)

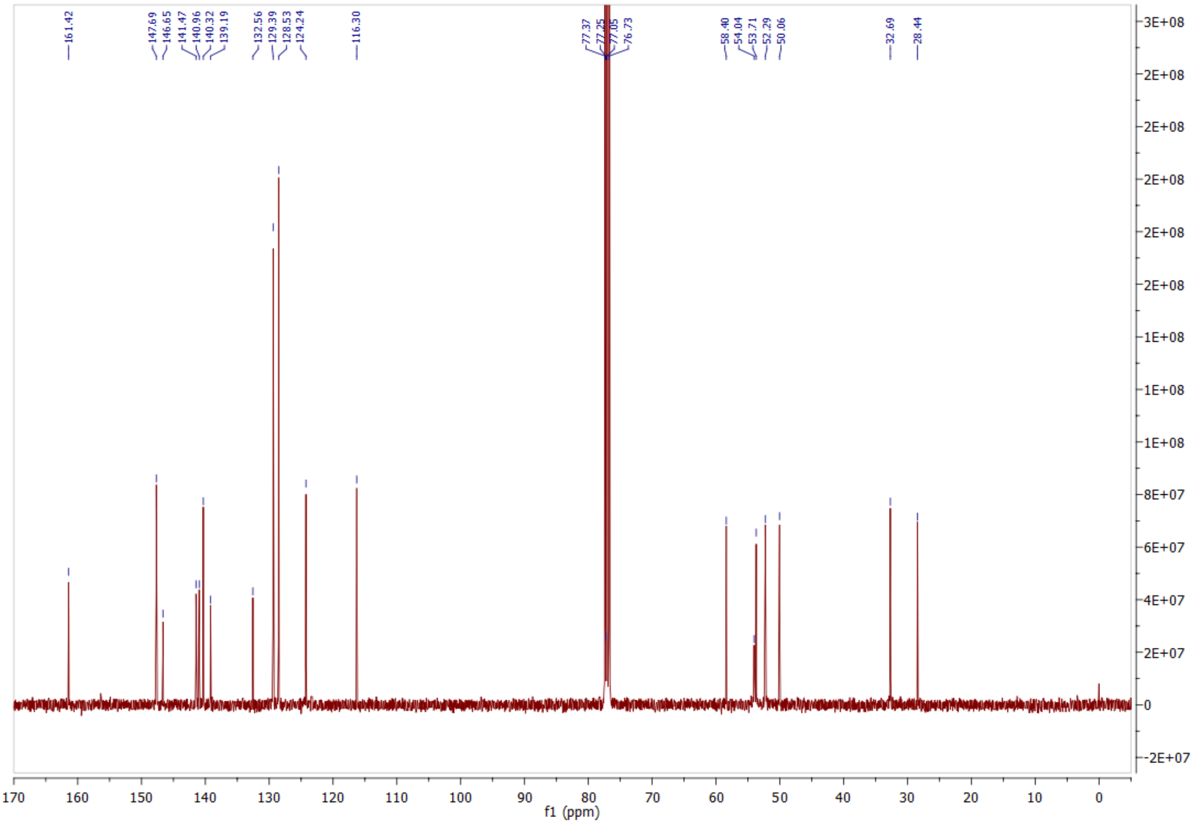

N-(4-bromobenzyl)-1-(2-(6-methoxy-1,5-naphthyridin-4-yl)ethyl)piperidin-4-amine (**5**)

^{1}H NMR (400 MHz, $CDCl_3$)

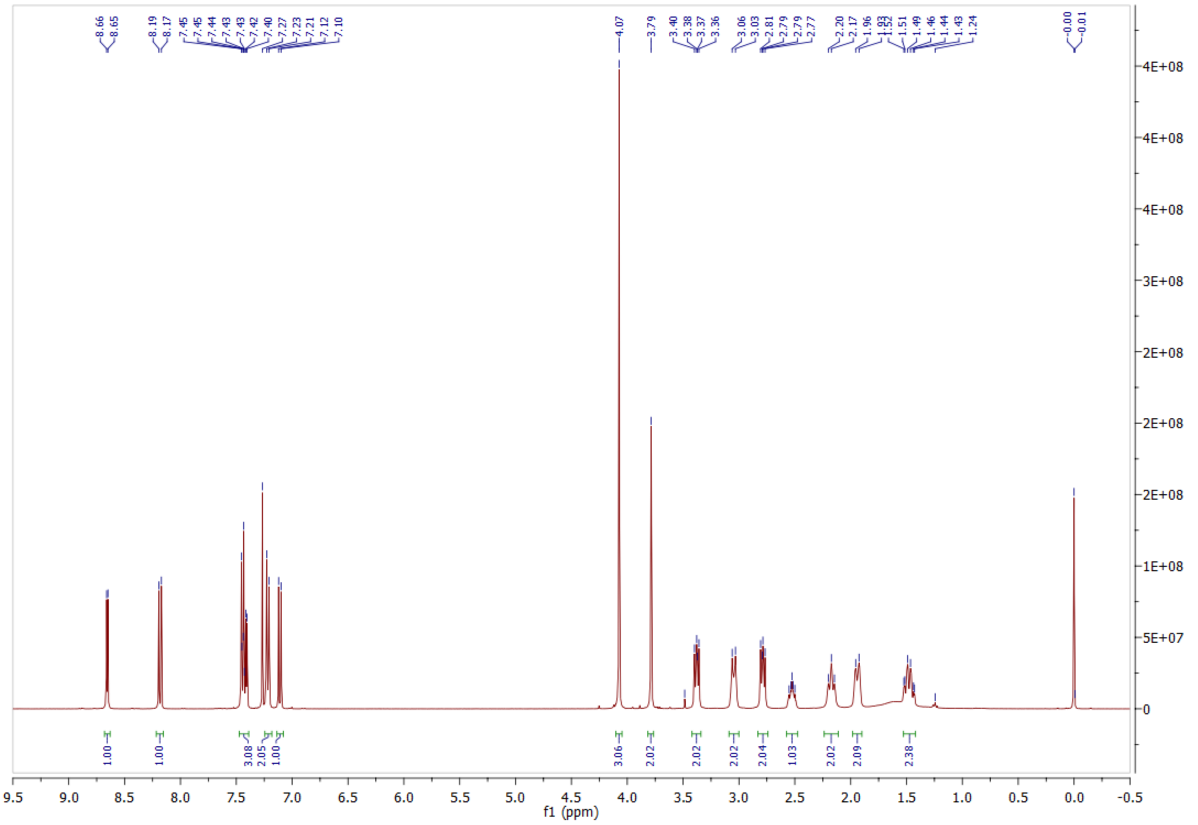

^{13}C NMR (100 MHz, $CDCl_3$)

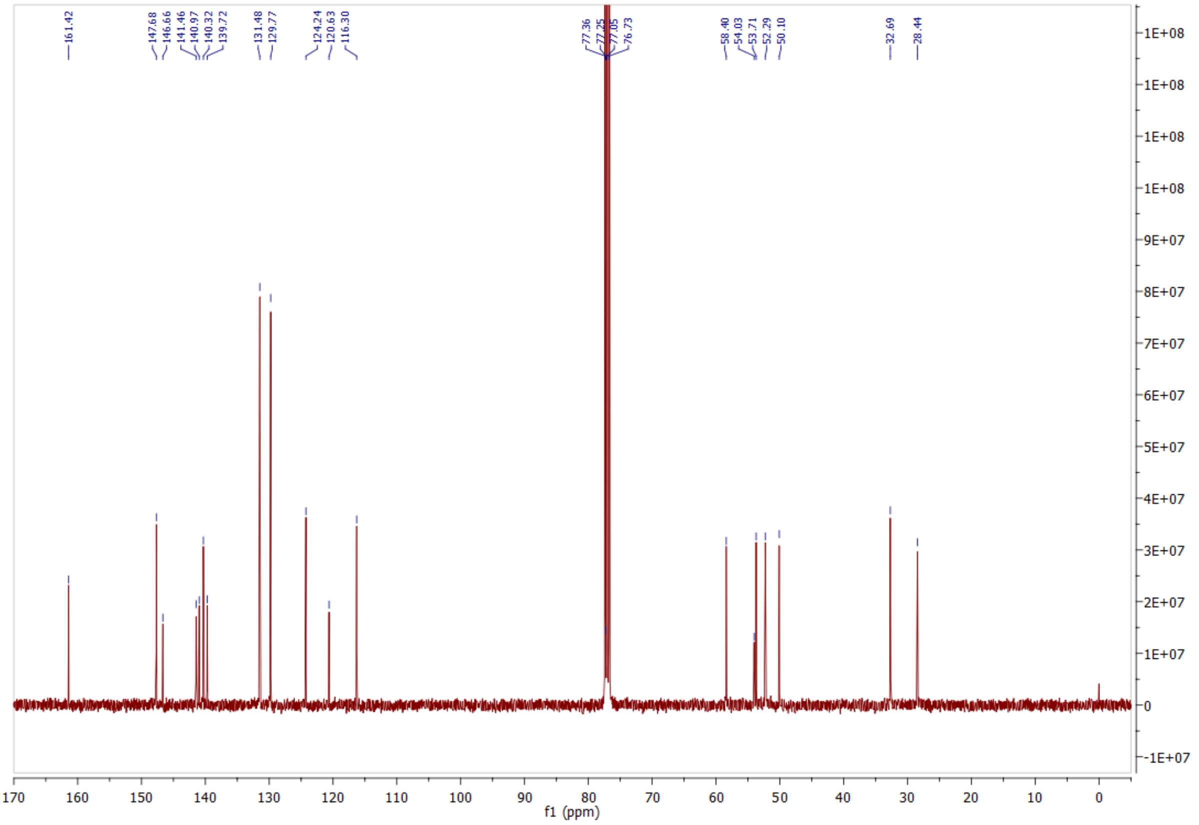

N-(4-iodobenzyl)-1-(2-(6-methoxy-1,5-naphthyridin-4-yl)ethyl)piperidin-4-amine (**6**)

^{1}H NMR (400 MHz, $CDCl_3$)

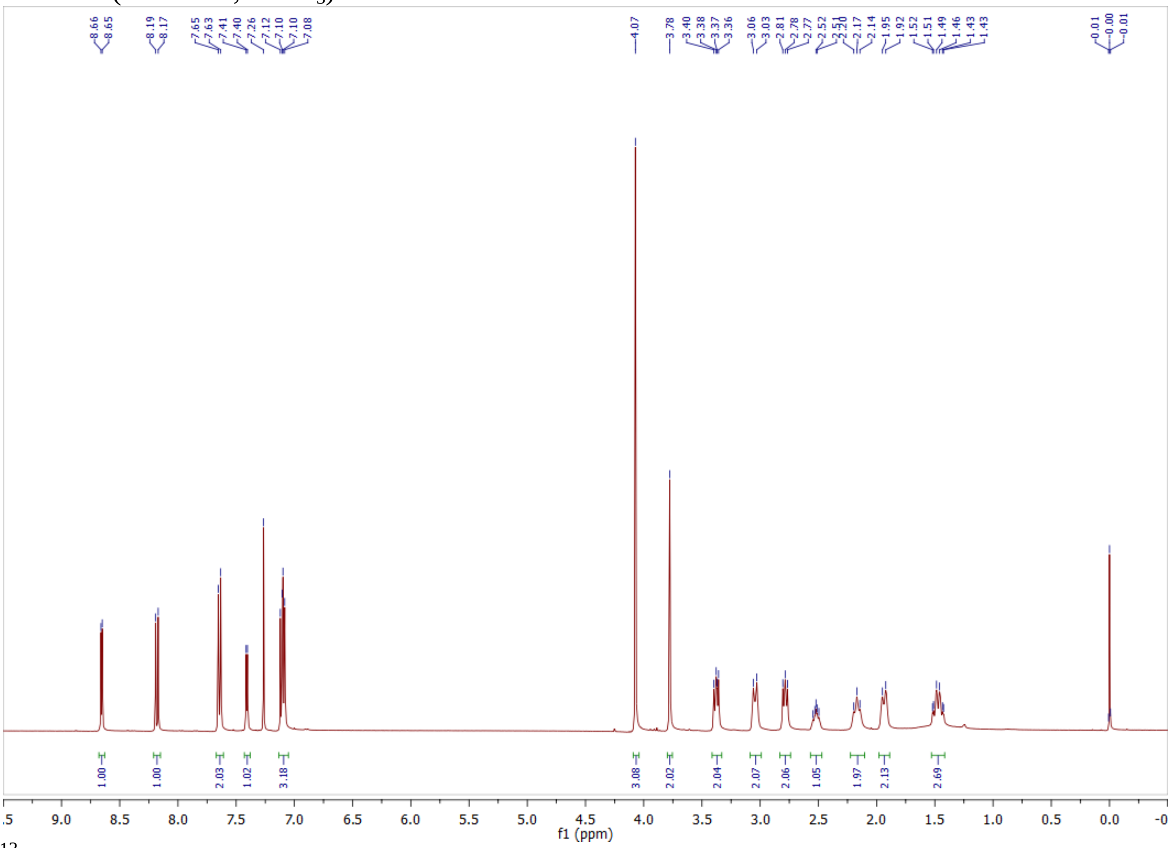

^{13}C NMR (100 MHz, $CDCl_3$)

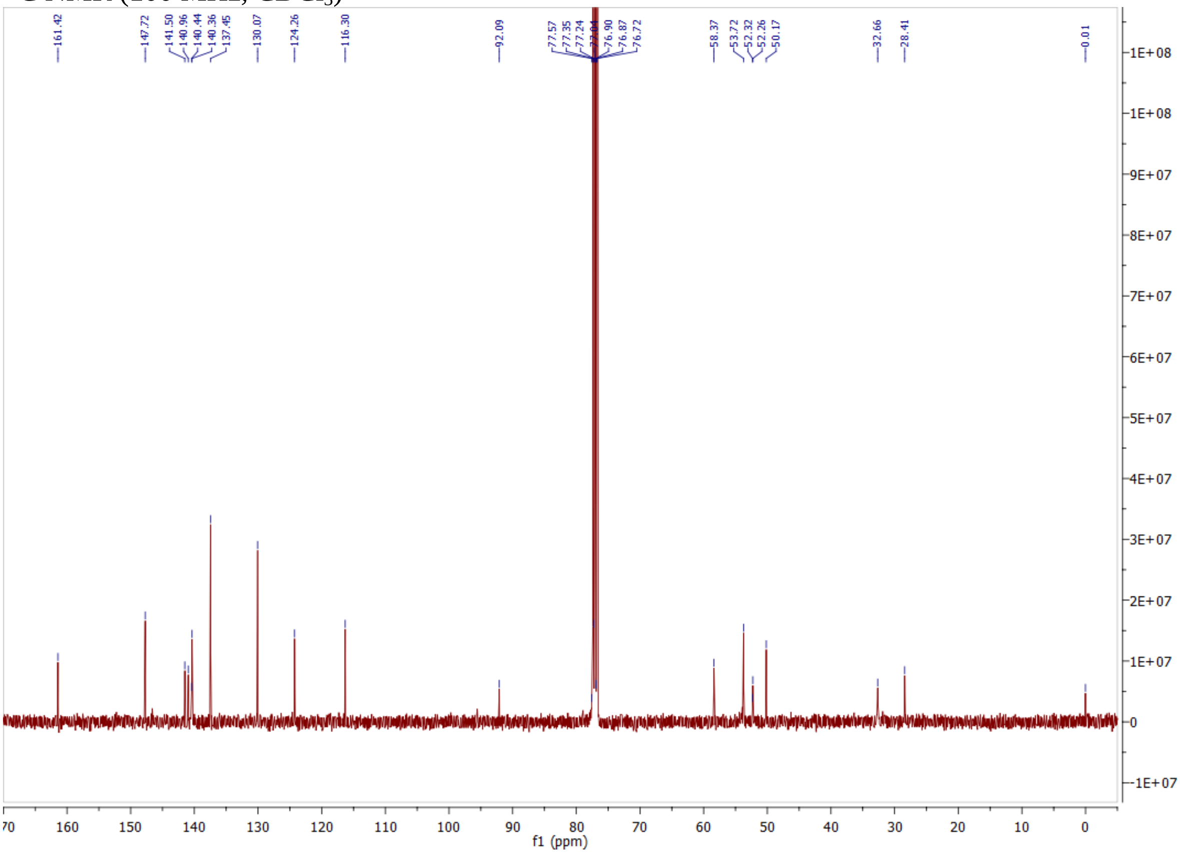

(4-((1-(2-(6-methoxy-1,5-naphthyridin-4-yl)ethyl)piperidin-4-ylamino)methyl)phenyl)methanol (**7**)

^{1}H NMR (400 MHz, $CDCl_3$)

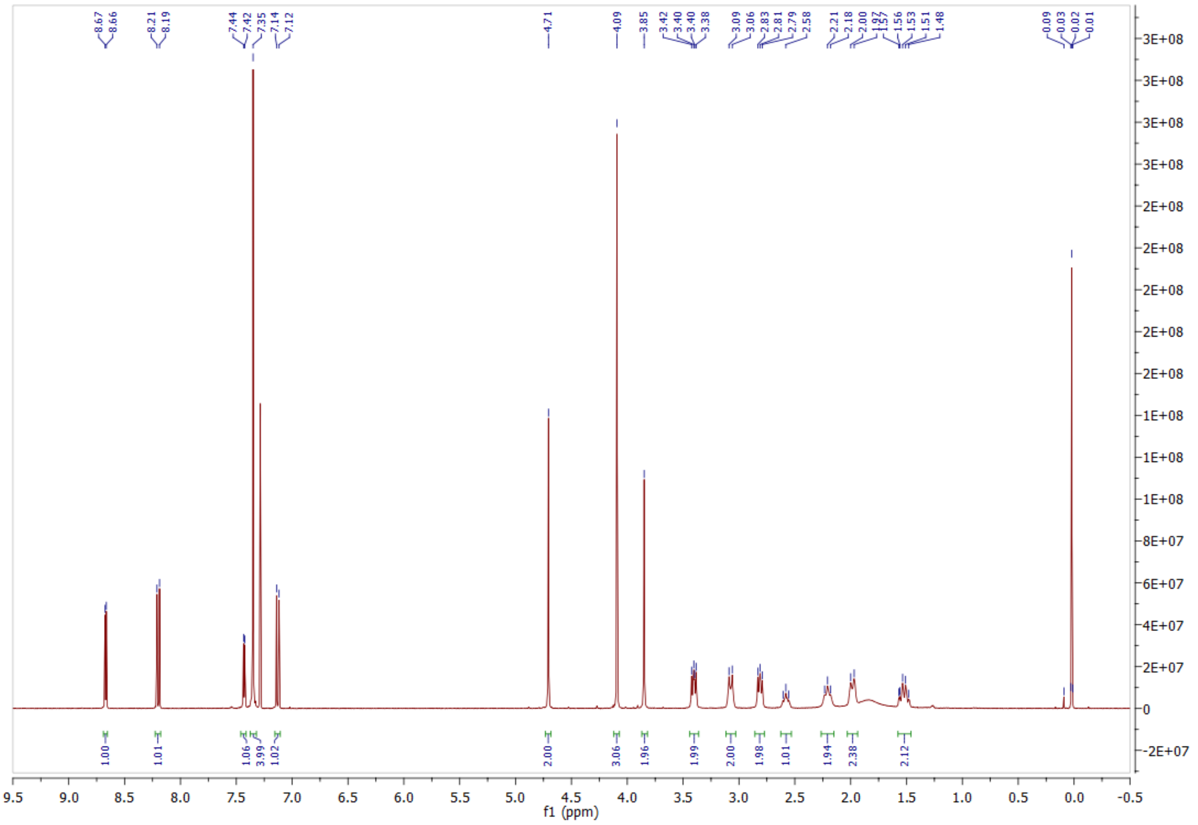

^{13}C NMR (100 MHz, $CDCl_3$)

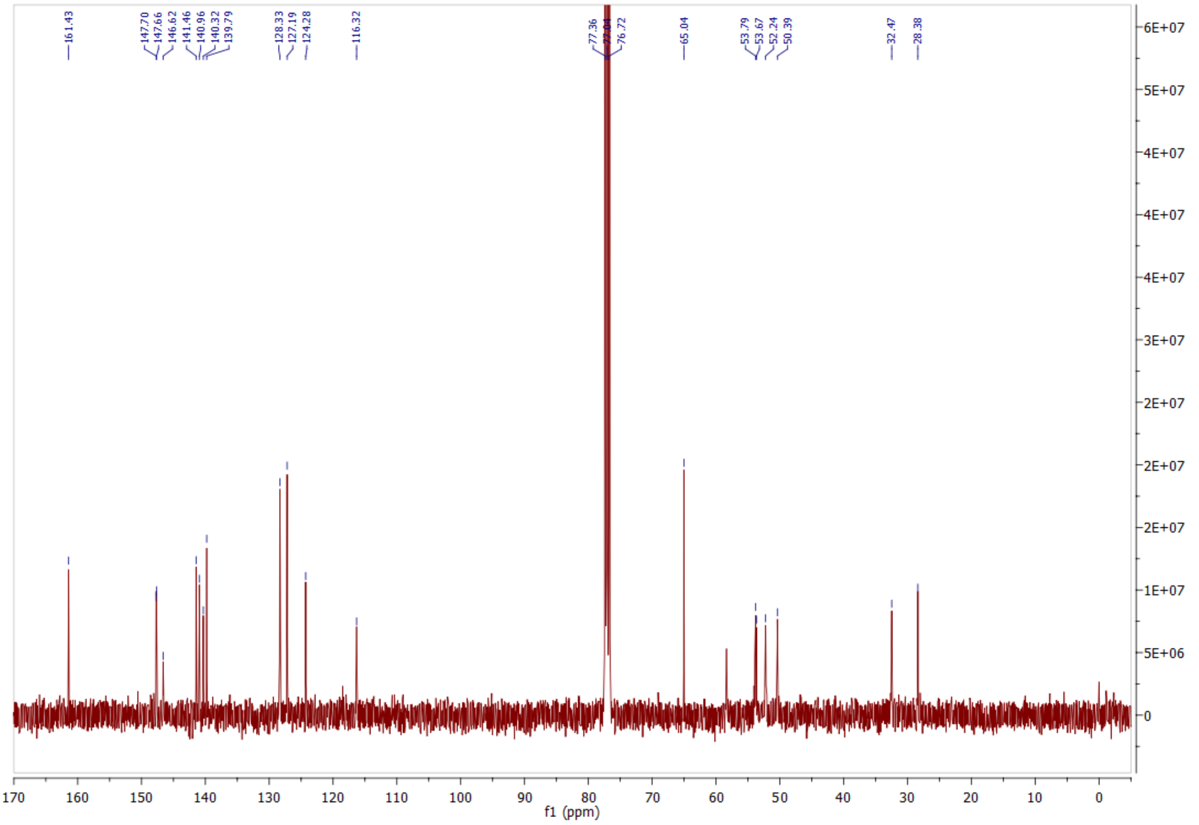

4-((1-(2-(6-methoxy-1,5-naphthyridin-4-yl)ethyl)piperidin-4-ylamino)methyl)benzamide (**8**)

^{1}H NMR (400 MHz, $CDCl_3$)

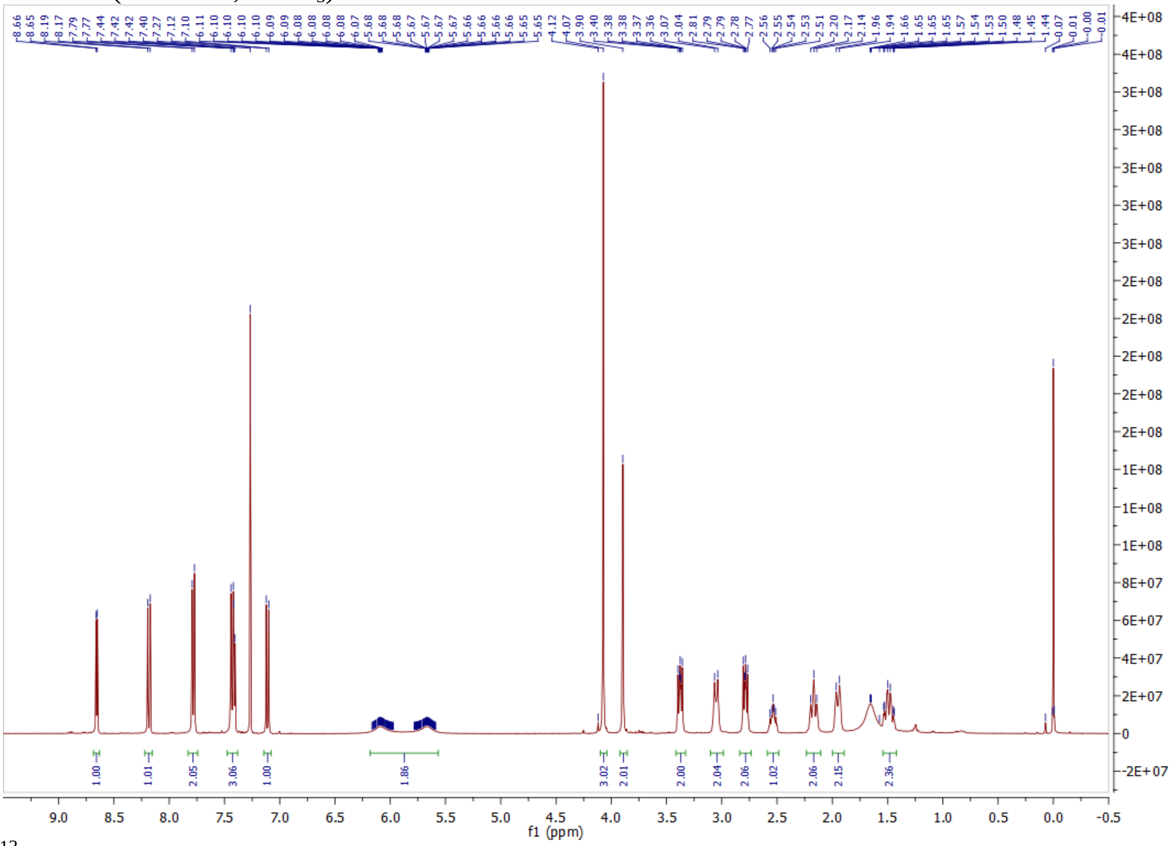

^{13}C NMR (100 MHz, $CDCl_3$)

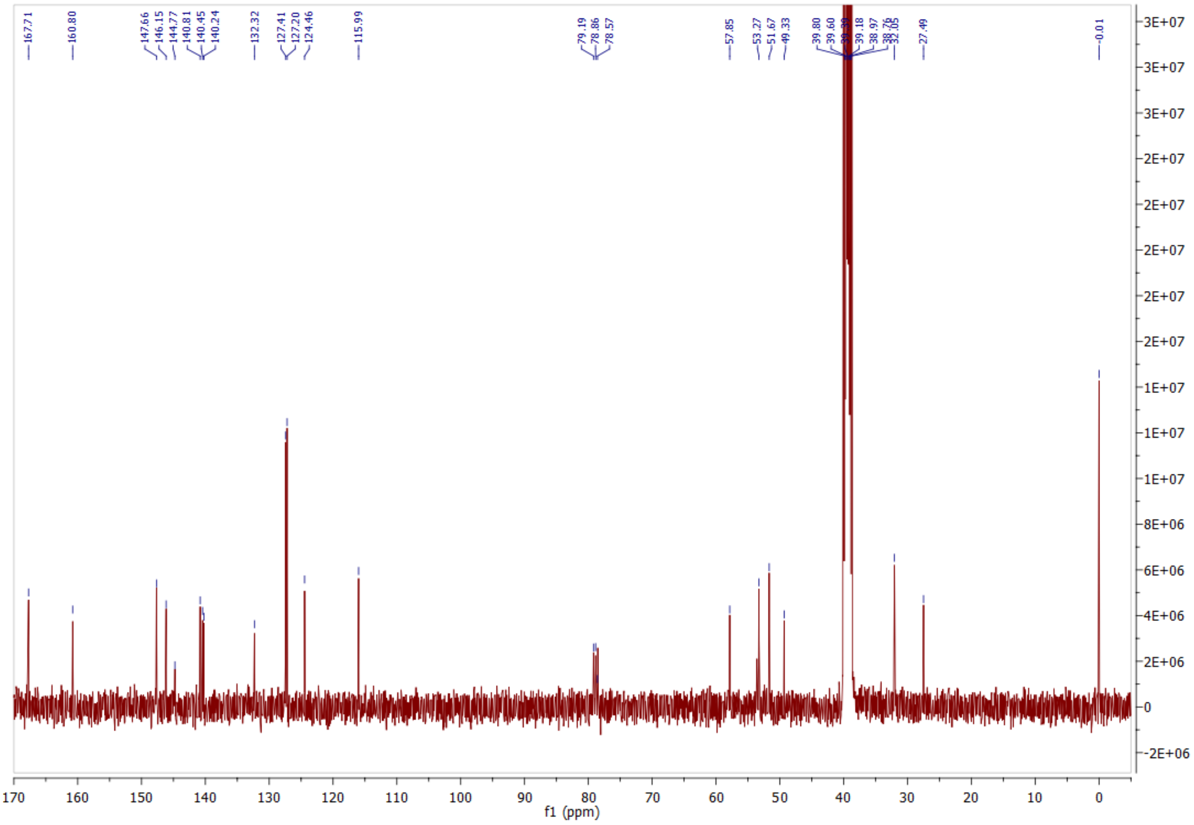

N-(4-(dimethylamino)benzyl)-1-(2-(6-methoxy-1,5-naphthyridin-4-yl)ethyl)piperidin-4-amine (**9**)

^{1}H NMR (400 MHz, $CDCl_3$)

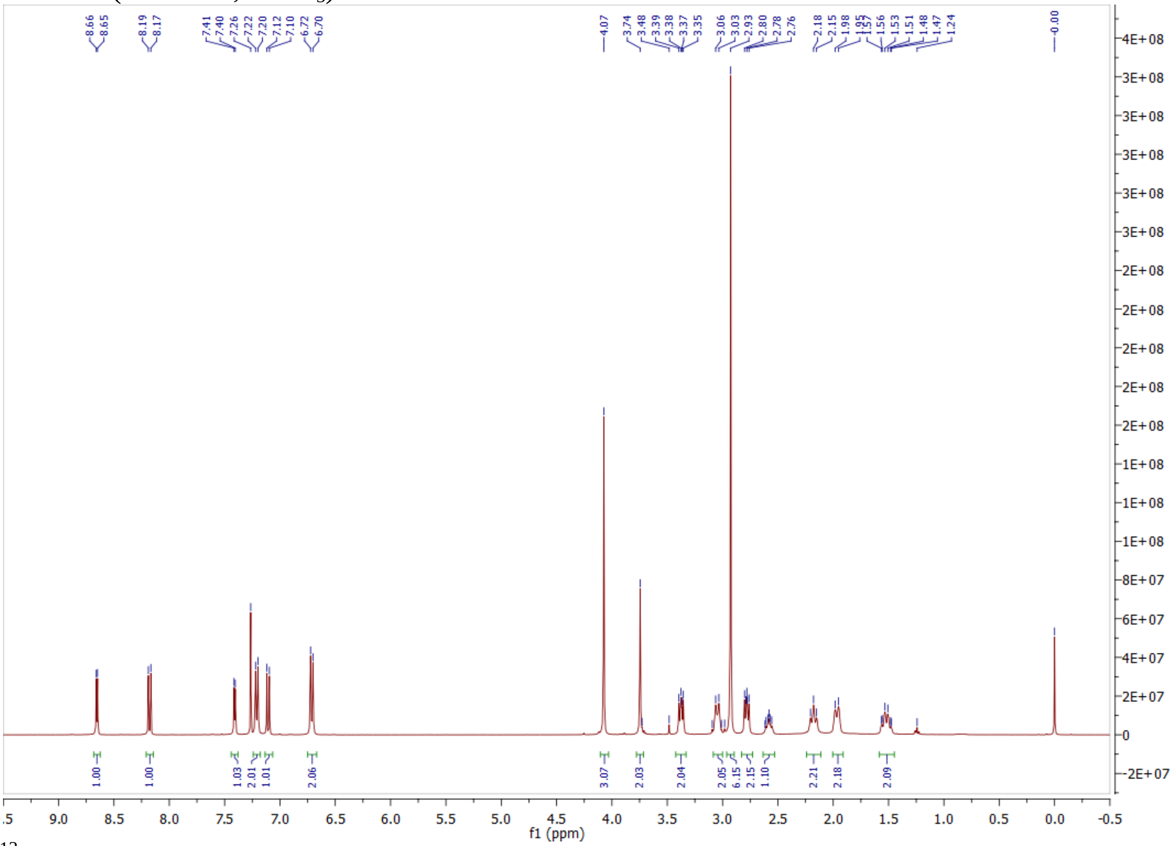

^{13}C NMR (100 MHz, $CDCl_3$)

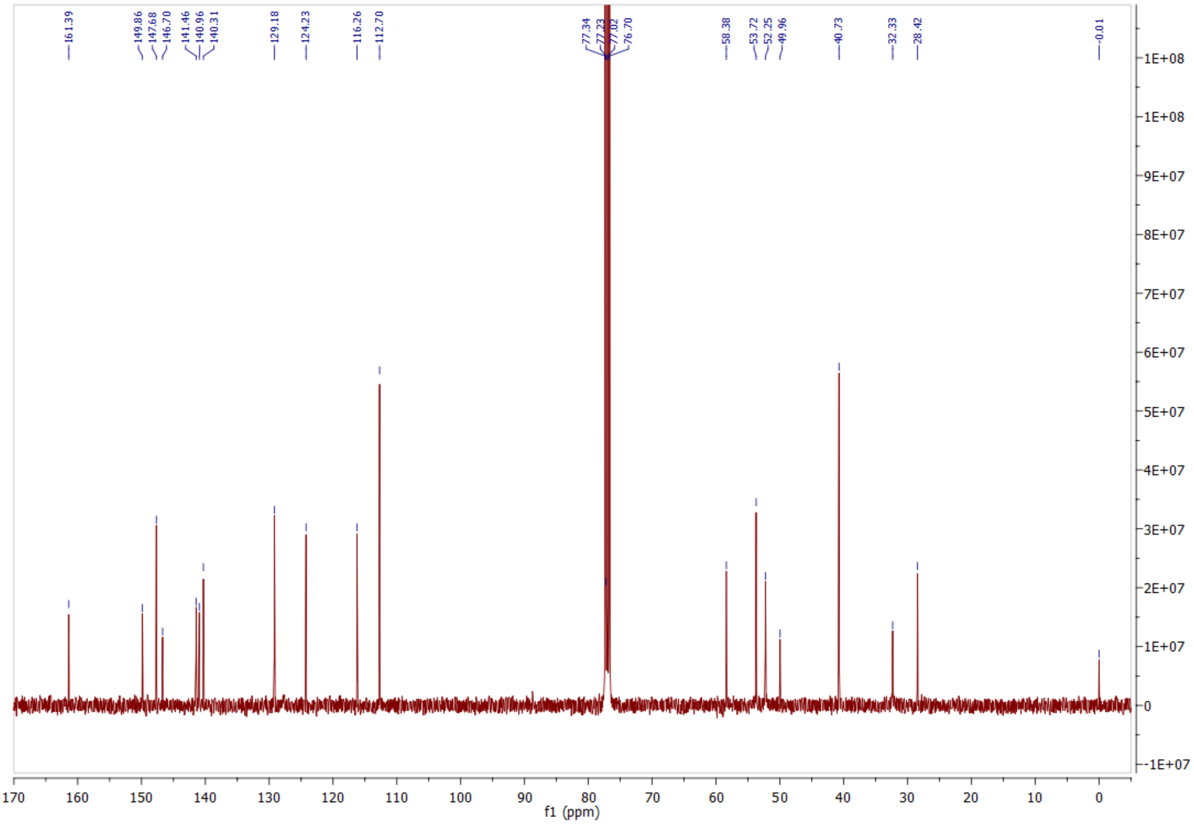

Determination of IC_{50} values of compounds 5 and 6 by using *S. aureus* gyrase supercoiling assays

Assay

In all experiments, the activity of the enzyme was determined prior to the testing of the compounds and 1 U defined as the amount of enzyme required to fully supercoil the substrate. This amount of enzyme was used in all subsequent assays. The final DMSO concentration in all the reactions was 1% (v/v). Compounds were serially diluted in DMSO and added to the reaction before the addition of the enzyme. Control compound for all assays was ciprofloxacin.

***Staphylococcus aureus* gyrase supercoiling assay**

1 U of DNA gyrase was incubated with 0.5 µg of relaxed pBR322 DNA in a 30 µl reaction at 37 °C for 30 min under the following conditions: 40 mM HEPES·KOH (pH 7.6), 10 mM magnesium acetate, 10 mM DTT, 2 mM ATP, 500 mM potassium glutamate and 0.05 mg/ml BSA. Each reaction was stopped by the addition of 30 µl chloroform/iso-amyl alcohol (24:1) and 20 µl Stop Dye (40% sucrose, 100 mM Tris.HCl (pH 7.5), 10 mM EDTA, 0.5 µg/ml bromophenol blue), before being loaded on a 1.0% TAE (Tris.acetate 0.04 mM, EDTA 0.002 mM). Gels run at 70V for 3 hours.

Data acquisition and analysis

Bands were visualised by ethidium staining for 10 min, destained for 10 min in water and bands were analysed by gel documentation equipment (Syngene, Cambridge, UK) and quantitated using Syngene Gene Tools software. Raw gel data (fluorescent band volumes) collected from Syngene, GeneTools gel analysis software were converted to a % of the 100% control (the fully supercoiled or relaxed DNA band) and plotted against the inhibitor concentration using SigmaPlot Version 13 (2015). The global curve fit non-linear regression tool was used to calculate IC_{50} data using the following equation: Equation: Exponential Decay, Single, 2 Parameter $f = ae^{-bx}$

Supplementary Table 6. IC_{50} results of *S. aureus* DNA gyrase supercoiling.

Compound	*S. aureus* gyrase supercoiling IC_{50} (µM)		
	Assay 1	Assay 2	**Average**
**Ciprofloxacin**	27.49	25.18	**26.34**
**5**	0.007	0.007	**0.007**
**6**	0.013	0.009	**0.011**

Assay 1

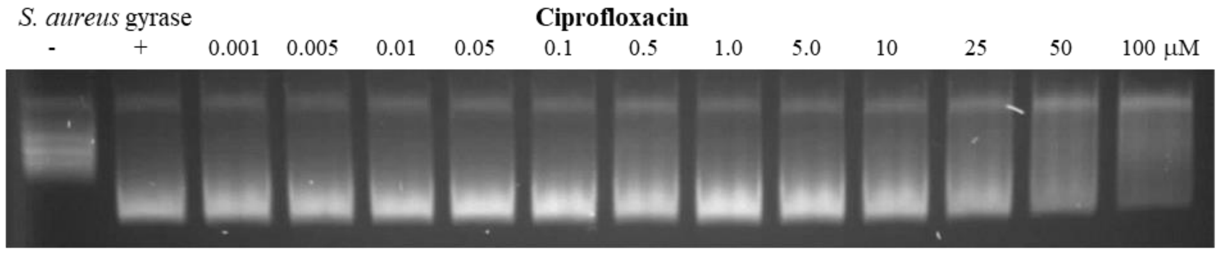

Assay 2

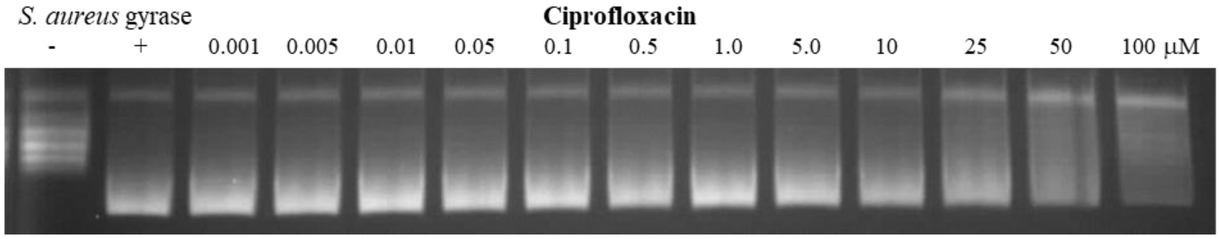

Supplementary Fig. 10. Gel Image. Inhibition of *S. aureus* DNA gyrase supercoiling by Ciprofloxacin.

Assay 1

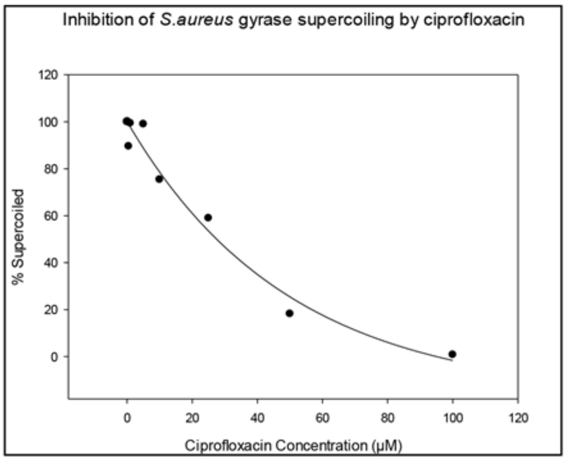

Assay 2

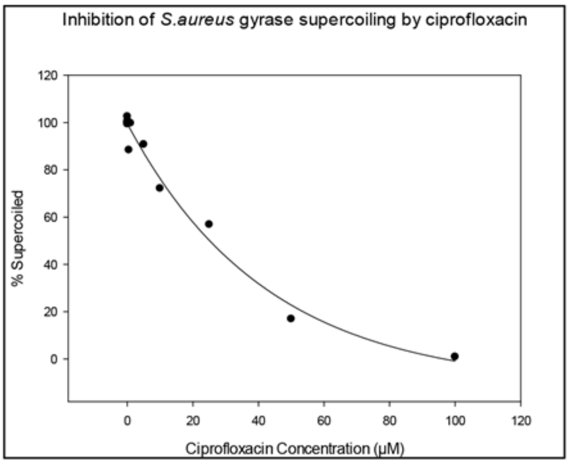

Supplementary Fig. 11. DNA gyrase supercoiling inhibition plot. The plot shows the results of the supercoiling inhibition with Ciprofloxacin. IC_{50} calculated from assay 1 is 27.49 μM and 25.18 μM from assay 2.

Assay 1

S. aureus gyrase **CFX** **5**

- + 25 50 0.001 0.005 0.01 0.05 0.1 0.5 1.0 5.0 10 25 50 100 μM

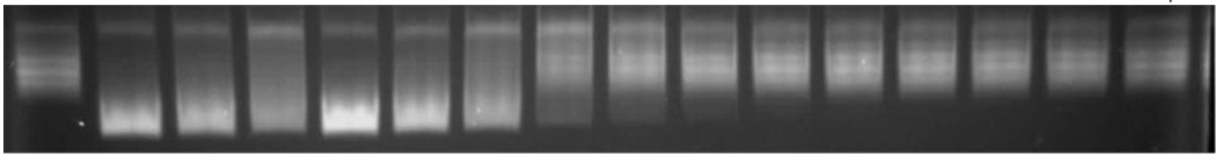

Assay 2

S. aureus gyrase **CFX** **5**

- + 25 50 0.001 0.005 0.01 0.05 0.1 0.5 1.0 5.0 10 25 50 100 μM

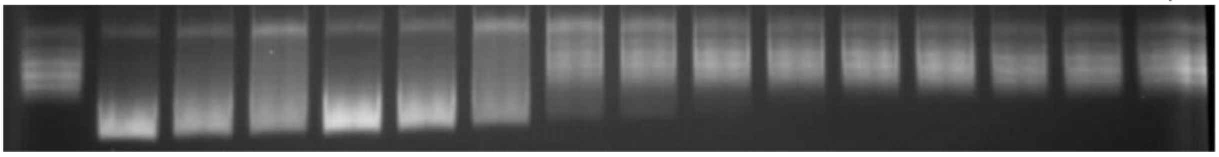

Supplementary Fig. 12. Gel Image. Inhibition of *S. aureus* DNA gyrase supercoiling by compound **5**. Ciprofloxacin (CFX) was used for comparison.

Assay 1

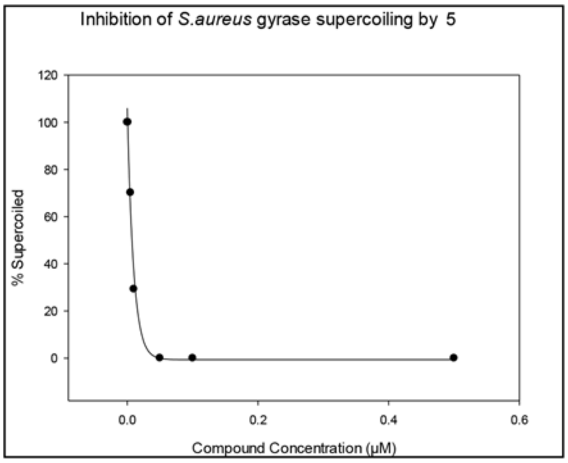

Assay 2

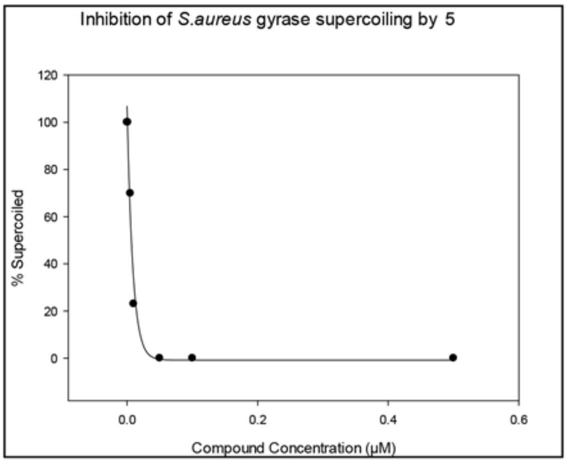

Supplementary Fig. 13. DNA gyrase supercoiling inhibition plot. The plot shows the results of the supercoiling inhibition with compound **5**. IC_{50} calculated from assay 1 is 0.007 μM and 0.007 μM from assay 2. Data were plotted up to 0.5 μM to improve the appearance of the plot.

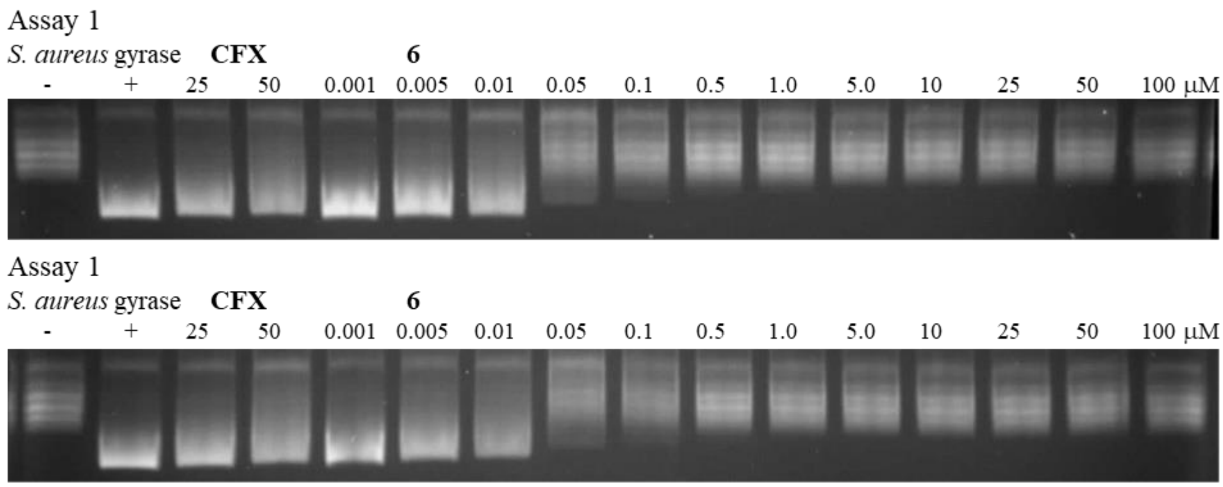

Supplementary Fig. 14. Gel Image. Inhibition of *S. aureus* DNA gyrase supercoiling by compound **6**. Ciprofloxacin (CFX) was used for comparison.

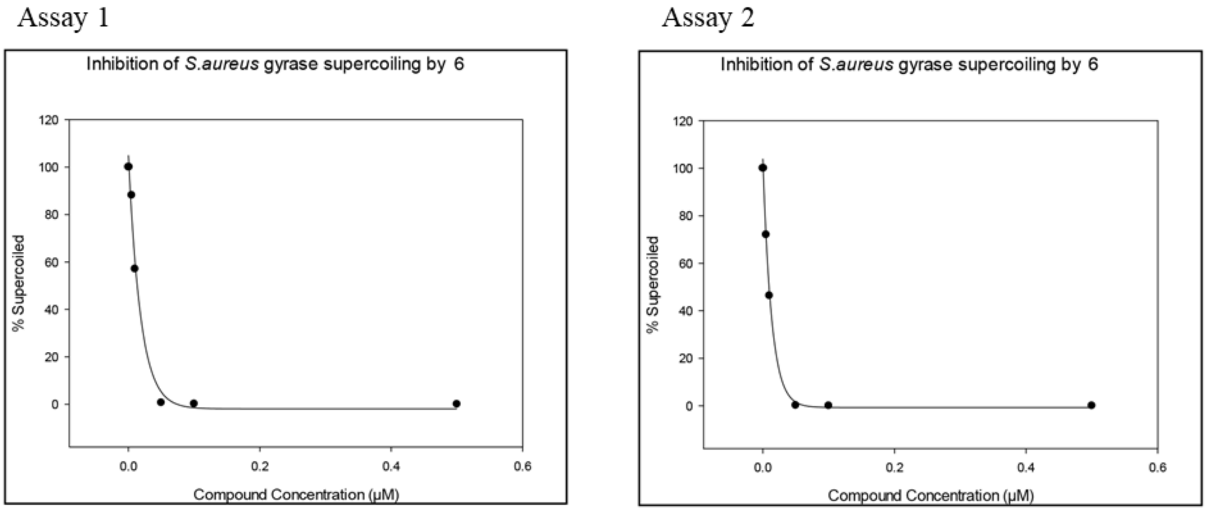

Supplementary Fig. 15. DNA gyrase supercoiling inhibition plot. The plot shows the results of the supercoiling inhibition with compound **6**. IC_{50} calculated from assay 1 is 0.013 μM and 0.009 μM from assay 2. Data were plotted up to 0.5 μM to improve the appearance of the plot.

Cleavage assay with *E. coli* DNA gyrase

The gels relate to the Fig. 3 in the main text.

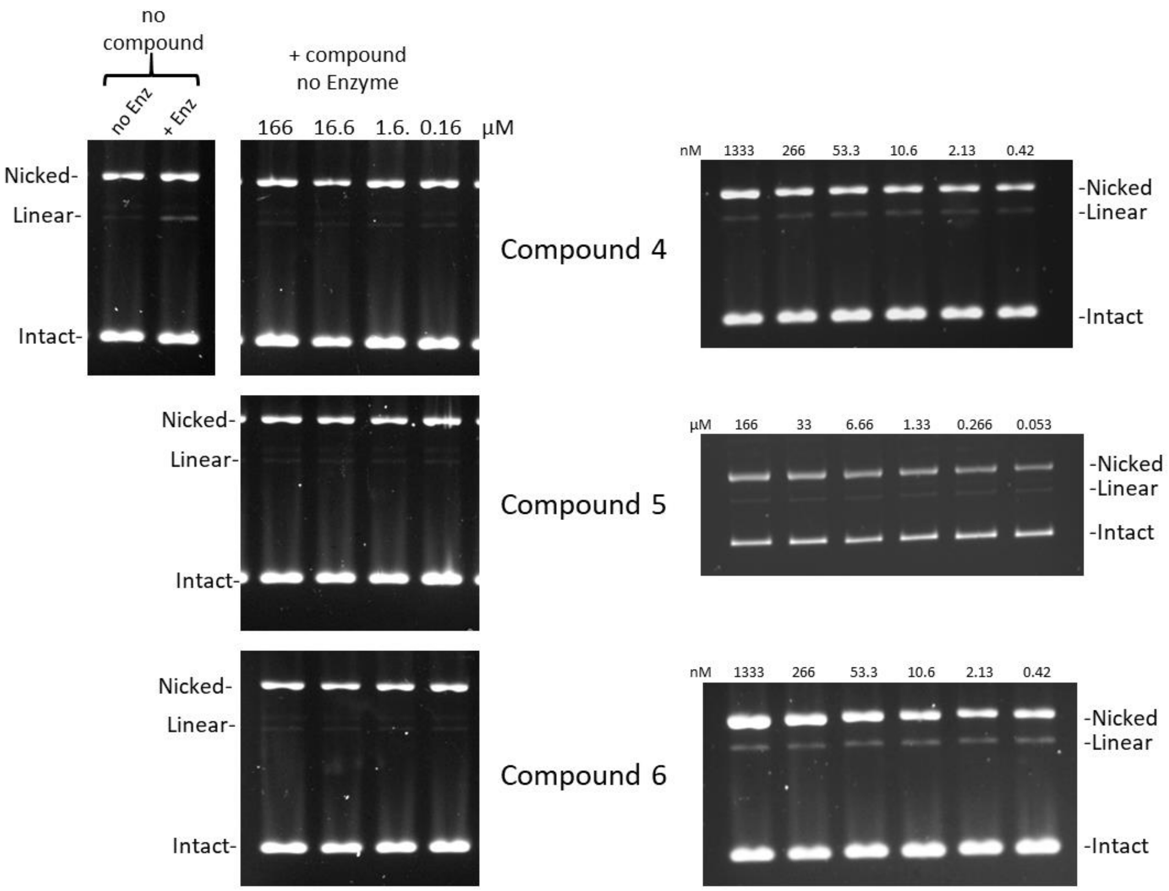

Supplementary Figure 16: Stabilisation of single-strand cleavage by the *E. coli* DNA gyrase with compounds 4, 5 and 6. Cleavage complexes formed by **4**, **5** and **6** were trapped with SDS (Materials and Methods) before treatment with Proteinase K. The plasmid substrate is mostly intact (top left) and thereby compacted by ethidium bromide, which is present in all the gels. Some nicking is observed. Addition of enzyme without any compound present induces a low amount of double-strand cleavage, which arise from the natural catalytic cycle of the enzyme. All three compound were tested without enzyme at a wide range of concentration (left column of gels) and no extra cleavage is observed (compared to the "no enzyme, no compound" lane). The right column of gels shows compounds tested with enzyme. The intensity of the nicked band increases with compound concentration, reflecting the stabilization of a singly cleaved cleavage complex by the compound. The level of cleavage remains relatively low when compared with the enzyme concentration (around 70 nM in all experiment). This is due to only a subset of enzyme-compound-DNA complexes being cleaved (see main text). The enzyme controls were reproduced twice with similar results and the cleavage assays (enzyme with compounds) were reproduced three times with similar results. Uncropped version of the gel image is available as Supplementary Figure 24.

Cytotoxicity studies.

Supplementary Table 7. Results of cytotoxicity studies.

Cmpd	HUVEC	HepG2	hERG
	IC_{50} [μM]	IC_{50} [μM]	IC_{50} [μM]
**2**	54.08 ± 3.45	17.67 ± 2.16	1.05
**3**	>50	12.67± 0.21	0.59
**4**	26.96 ± 0.38	10.16 ± 0.10	0.27
**5**	25.42 ± 0.01	9.37 ± 0.07	0.29
**6**	18.98 ± 0.85	8.47 ± 0.09	0.18
**7**	>50	nd	3.24
**8**	>50	nd	13.45
**9**	23.85 ± 0.09	10.03 ± 0.06	2.11

nd: not determined

Metabolic activity assessment

Cells of the HepG2 line (ATCC® HB-8065™) and HUVEC (ATCC® CRL-1730™) were cultured in Dulbecco's Modified Eagle Medium (DMEM; Sigma-Aldrich, St. Louis, MO, USA) supplemented with 10% heat-deactivated FBS (Gibco, Grand Island/NY, USA), 2 mM L-glutamine, 100 U/mL penicillin, 100 μg/mL streptomycin (all from Sigma-Aldrich) in a humidified chamber at 37 °C and 5% CO_2. The experiments were carried out on passages 7 - 9 for both cell lines. The tested compounds were dissolved in DMSO and further diluted in culture medium to a desired final concentration. Cells were seeded into 96-well plates at 8×10^4 cells/mL (100 μL/well) and incubated for 24 h to attach onto the wells. Cells were treated with 1 μM and 50 μM of each compound of interest or corresponding vehicle as control. For IC_{50} determination, cells were seeded into 96-well plates at 5×10^4 cells/mL (100 μL/well) and after attachment treated with seven different concentration of the selected compounds or corresponding vehicle as control. The metabolic activity was assessed after 72 h treatment using the CellTiter 96 Aqueous One Solution Cell Proliferation Assay (Promega, Madison/WI, USA), in accordance with the manufacturer's instructions.[7] The absorbance was measured at 492 nm on an automated microplate reader BioTek Synergy HT (BioTek Instruments, Inc.). Data are presented as IC_{50} value (mean ± SD) from two independent experiments, each conducted in duplicate or triplicate. IC_{50} values were calculated by a non-linear regression model using Graph Pad Prism 7 software.

Cardiovascular hERG inhibition

Cardiovascular safety profile was entirely outsourced. hERG screening was performed at TCG Lifesciences Pvt. Ltd. India with FP-based hERG binding assay (Invitrogen kit).

Results of pdb database search

We have searched the RCSB Protein Data Bank for biological complexes in which a ligand is able to form bifurcated halogen bond with the protein. Recently, Shinada et al. performed a detailed RCSB PDB search, identifying bifurcated halogen bonds in five protein-ligand complexes (1GJD, 1UHI, 4CMJ, 4JYI, and 5A86)[8]. Our search was performed with advanced search of the Structure title, Primary Citation title, and PubMed abstract fields, utilizing keywords "bifurcated halogen bonds", "symmetrical halogen bonds", and "two halogen bonds". Two complexes (5YC6 and 5YC7) arose from this search. Hits for all complexes are exemplified in **Tables S7** and **Figure S11,** along with information about X···O distances, Θ_1, and Θ_2 angles.

Supplementary Table 8. PDB database search results of ligands forming bifurcated halogen bond with protein.

PDB ID	Halogen involved	d$_1$ [Å]'	d$_1$ [Å]''	Θ_1 [°]'	Θ_1 [°]''	Θ_2 [°]'	Θ_2 [°]''
**1GJD**	I	3.4	3.7	163.9	143.3	156.3	133.6
**1UHI**	I	4.5	3.1	147.4	174.2	136.1	127.4
**4CMJ**	Br	4.4	3.8	143.8	142.4	78.0	142.2
**4JYI**	Cl	3.9	3.3	141.8	150.5	97.8	140.0
**5A86**	Cl	3.7	4.4	140.4	141.1	68.2	141.4

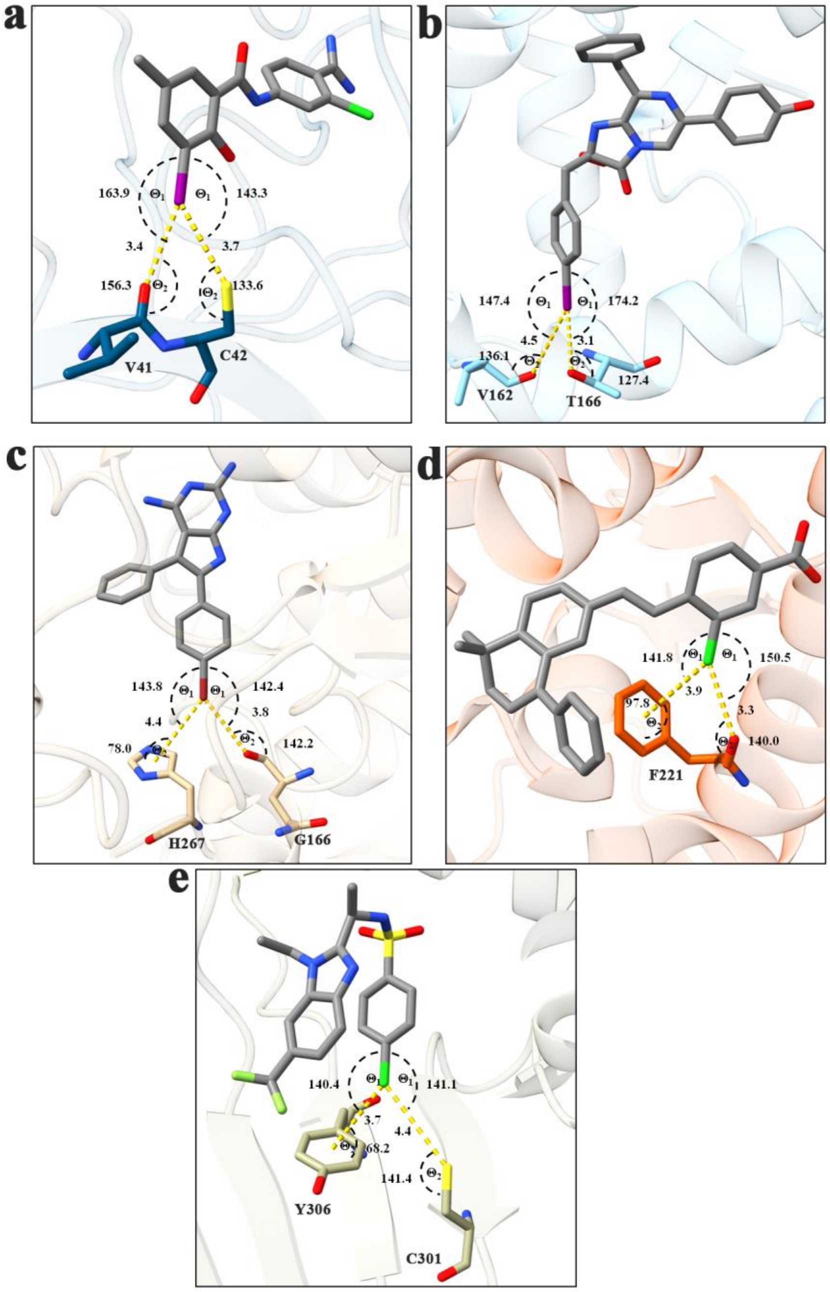

Supplementary Figure 17: Results of pdb databank search. **a** 1GJD (protein in cartoon representation in blue); **b** 1UHI (protein in cartoon representation in cyan); **c** 4CMJ (protein in cartoon representation in tan); **d** 4JYI (protein in cartoon representation in orange); **e** 5A86 (protein in cartoon representation in yellow). Amino acid residues are represented as sticks colour-coded by element. Halogen bonds are represented as yellow dots.

Results of small molecule database (Cambridge Structural Database (CSD)) search

The search of small molecules in Cambridge Structure Database[9] (CSD version 2020.1), able to form bifurcated halogen bonds was performed using ConQuest (version 2020.1.1) program with the following criteria: halogen atom (Cl, Br or I) is bound to phenyl and interacts with two carbonyl oxygen atoms. All the obtained structures were visually inspected using Mercury (version 2020.1). Those structures with the distances between the halogen atom and both oxygen atoms lower than the sum of van der Waals radii were selected as hits. As reported by Shinada et al. a majority of chlorine atoms forms halogen bonds at a distance 0.3 Å longer than the sum of van der Waals radii.[8] Therefore we applied this criteria for another selection of structures with a chloro atom. Although the search was based on halogen bonding with carbonyl oxygen atoms, structures in which halogen forms halogen bond with two non-carbonyl oxygens or one carbonyl and one non-carbonyl were also considered as hits. Hits for all halogen atoms are exemplified in **Tables S8-11** along with information about X···O distances, Θ_1, and Θ_2 angles. Statistics on their geometric parameters are shown on **Fig. S12-15**.

Supplementary Table 9. Summary of hits from CSD search, forming bifurcated Cl···O bonds at the distance lower than the sum of van der Waals radii.

No	Crystal structure identifier	d_1 [Å]’	d_2 [Å]’’	Θ_1 ° [C-X···O]’	Θ_1 ° [C-X···O]’’	Θ_2 ° [X···O-R]’	Θ_2 ° [X···O-R]’’
1	KOVJEN	2.951	3.229	166.01	149.41	74.3	131
2	POZHOE	2.917	2.623	150.1	114.11	101.54/135.64	124.82
3	IJOCIT	3.254	3.18	155.91	136.63	104.73/114.02	103.32/94.89
4	MAVZER	3.268	3.238	89.64	129.57	73.85	74.93/90.8
5	SAGRIF	3.124	3.259	160.82	96.31	125.07	154.29

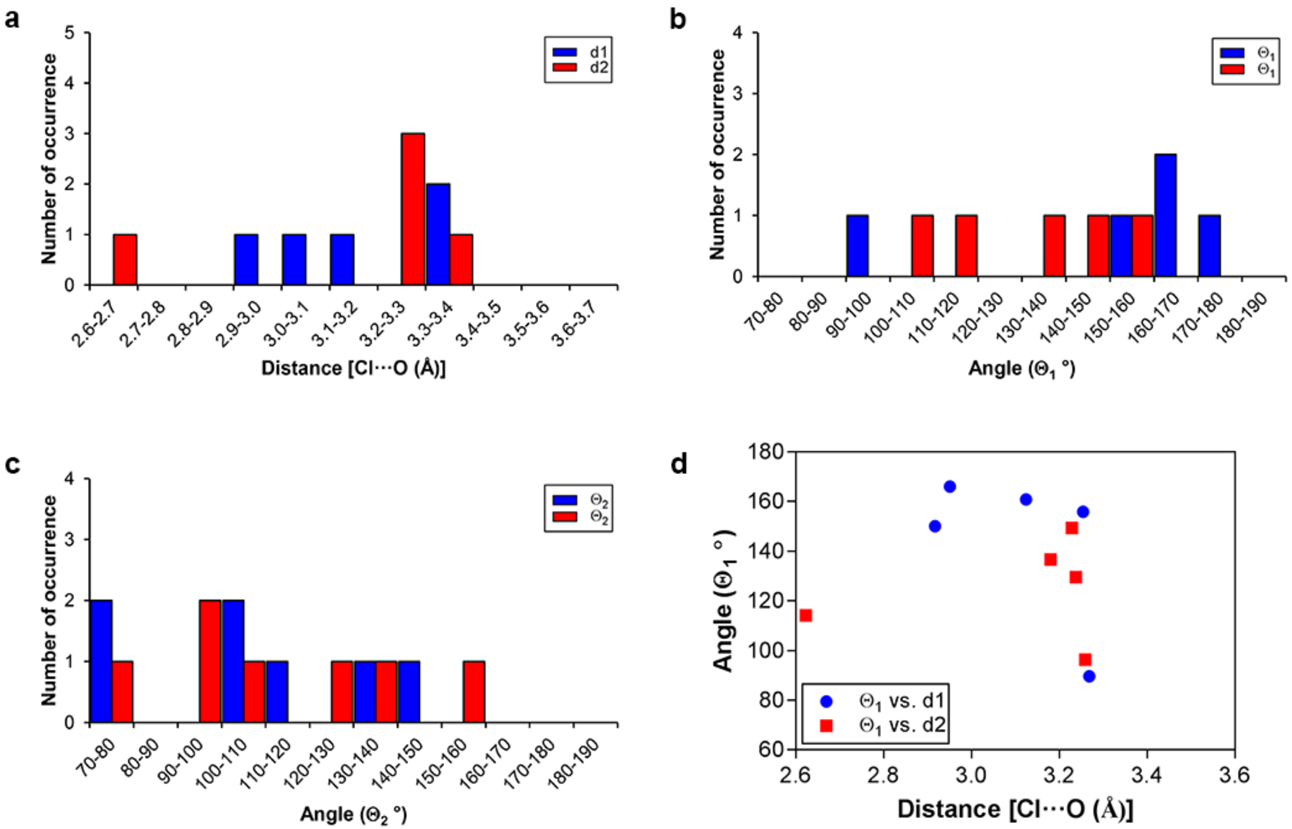

Supplementary Figure 18: Statistics on geometrical properties of bifurcated Cl···O halogen bond based on sum of van der Waals radii. a Distribution plot of bifurcated Cl···O halogen distances. **b** Distribution plot of Θ_1 [C-Cl···O] angles. **c** Distribution plot of Θ_2 [C···O-R] angles. **d** Scatter plots (angle (Θ_1) versus distance) obtained from the halogen bond CSD search. Distance between halogen atom and first oxygen, and associated Θ_1, Θ_2 angles are represented as blue bars, while distance between halogen atom and second oxygen, and associated Θ_1, Θ_2 angles are represented as red bars.

Supplementary Table 10. Summary of hits from CSD search, forming bifurcated Cl···O bonds at the distance lower than the sum of van der Waals radii + 0.3 Å.

No	Crystal structure identifier	d_1 [Å]’	d_2 [Å]’’	d_3 [Å]’’’	Θ_1 ° [C-X···O]’	Θ_1 ° [C-X···O]’’	Θ_1 ° [C-X···O]’’’	Θ_2 ° [X···O-R]’	Θ_2 ° [X···O-R]’’	Θ_2 ° [X···O-R]’’
1	**GUMNOU**	3.429	3.451	3.179	112	143.4	143.5	112.7	120.9	146.7
2	**JUDPEG**	3.095	3.454	/	154.52	92.04	/	116.7	166.5	/
3	**KOQCIF**	3.415	3.46	/	93.1	136.9	/	90.2	87.3	/
4	**KOVJEN**	3.493<br>2.951	3.279<br>3.229	/	109.73<br>166.01	175.14<br>149.41	/	103.24<br>74.3	145.67<br>131	/
5	**LOYHOZ**	3.548	3.48	/	100.03	120.11	/	111.33/75.62	132.43	/
6	**NUBDEW**	3.485	3.437	/	168.49	74.02	/	123.5	82.53	/
7	**POZHOE**	2.917	2.623	/	150.1	114.11	/	101.54/135.64	124.82	/
8	**PUGQOA**	3.549	3.276	/	92.12	174.64	/	91.36	101.74	/
9	**QOWWEH**	3.416	3.413	/	99.51	92.89	/	107.98/128.01	110.52	/
10	**ZONTOO**	3.46	3.289	/	64.03	132.62	/	87.27	90.93	/
11	**AJAKUR**	3.537	3.42	/	70.66	85.1	/	119.59	77.31	/
12	**AKOZEF**	3.489	3.149	/	75.02	160.52	/	111.47	131.3	/
13	**ANEQUH**	3.458	3.46	/	152.8	124.72	/	100.71/106.33	101.33	/
14	**ANUQIL**	3.429	3.547	/	150.08	113.98	/	115.79	105.51/135.83	/
15	**APUGEY**	3.492	3.37	/	152.31	123.77	/	105.56/125.51	141	/
16	**ASOPEE**	3.44	3.257	/	82.19	137.86	/	132	97.64	/
17	**ASUMUX**	3.247	3.332	/	124.94	148.19	/	102.95/86.04	128.82	/
18	**AWEJIX**	3.428	3.41	/	160.48	124.94	/	149.21	97.76	/
19	**AWITAB**	2.952	3.407	/	165.9	71.27	/	129.76	149.66	/
20	**BACBUC**	3.559	3.558	/	103.62	90.73	/	163.98	138.04	/
21	**BACDAK**	3.493	3.506	/	104.47	91.85	/	163.27	136.13	/
22	**BANQUD**	3.404	3.36	/	131.09	147.94	/	113.31	82.38/98.62	/
23	**BARTAP**	3.518	3.472	3.538	138.23	121.02	164.43	93.78	95.85	94.83/105.87
24	**BEBCAM**	3.445	3.461	/	151.48	139.41	/	80.14/129.52/81.88	83.11	/
25	**BETXOQ**	3.238	3.258	/	131.4	166.26	/	143.68	148.17	/
26	**BICWOC**	3.404	3.468	/	109.7	139.51	/	75.42	72.22/89.64	/
27	**BIHXIA**	3.207	3.365	/	154.93	143.18	/	143.05	144.49	/
28	**BIPFUE**	3.515	3.315	/	154.43	143.08	/	116.13/99.09	128.41/95.97	/
29	**BIPGAL**	3.305	3.494	/	143.5	155.4	/	128.5/94.5	98.5/116.2	/
30	**BIVNUP**	3.541	3.559	/	96.37	126	/	101.6/67.16	66.41	/

31	**BIZFUM**	3.397	3.097	/	78.16	174.31	/	101.28	136.96	/
32	**BONPOM**	3.291	3.461	/	138.26	83.52	/	138.92	102.85	/
33	**BUJLEZ**	3.568	3.462	3.532	92.11	134.37	147.05	154.47	156.59	130.63/76.39
34	**BUSQUD**	3.44	3.522	/	91.88	128.63	/	73.15	69.39/97.97	/
35	**CAJSAK**	3.481	3.135	/	75.3	150.71	/	100.55	98.69	/
36	**CEDWAL**	3.404	3.558	/	71.36	100.71	/	96.67	81.13	/
37	**CIGWUK**	3.531	3.039	/	102.12	175.2	/	116.88	133.21/109.73	/
38	**CIJFEI**	3.562	3.518	/	154.33	159.17	/	83.79	85.59	/
39	**CIJWAV**	3.308	3.526	/	157.65	116.8	/	118.04	153.99	/
40	**CIMHIP01**	3.142	3.543	/	150.82	133.18	/	147.08	106.18/115.6	/
41	**CIPZIL**	3.449	3.415	/	158.68	68.38	/	97.14	109.4	/
42	**CIVWAH**	3.526	3.143	/	89.84	168.1	/	115.5/90.23	92.1	/
43	**CNAPAN**	3.439	3.118	/	144.53	177.24	/	146.82/94.06	115.06	/
44	**COLNAT**	3.458	3.533	/	134.7	159.34	/	137.51	97.09	/
45	**COSWOY**	3.466	2.772	/	161.81	153.07	/	92.74/54.62	83.95	/
46	**CUQZAR**	3.294	2.855	3.387	107.8	170.95	138.9	64.59/91.98	108.05	84.59
47	**DAQZED**	3.516	3.515	/	116.87	142.73	/	81.71	82.11	/
48	**DEJKEL**	3.107	3.478	/	166.79	118.64	/	107.21/113.43	149.91/94.3	/
49	**DEJMAI**	3.435	3.44	/	125.92	125.6	/	104.5	137.34/89.56	/
50	**DICNOU**	3.072	3.356	/	138.08	94.44	/	153.73	150.87	/
51	**DIPHUH01**	3.459	3.429	/	78.41	81.35	/	92.69	134.25	/
52	**DISHOE**	3.344	3.514	/	120.28	104.84	/	84.86	77.31	/
53	**DIXFUO**	3.376	3.481	/	166.83	66.33	/	154.78	134.02	/
54	**DIYCAS**	3.463	3.384	/	148.8	146.85	/	104.68	121.1	/
55	**DOGBUW**	3.415	3.513	/	131.53	77.36	/	78.29	104.56	/
56	**DOKJUL**	3.191	3.108	/	141.36	142.07	/	110.01/112.79	117.38/123.24	/
57	**DOMDAL**	3.395	3.547	/	69.4	66.77	/	136.79/101.48	105.69/53.04	/
58	**DOXQIQ**	3.49	3.359	/	138.4	93.69	/	148.19	156.96	/
59	**DOZQOA**	3.536	3.293	/	101.52	164.99	/	70.54	86.52	/
60	**DUKDUI**	3.426	3.456	/	91.74	133.76	/	127.87	138.79	/
61	**DUKHAT**	2.976	3.439	/	166.43	102.04	/	119.27/115.02	119.27	/
		3.458	3.293		107.16	97.23		99.26/75.78	103.63	
62	**DUPVOB**	3.455	3.263	/	148.07	159.93	/	155.72	154.74	/
63	**EBITEQ**	3.266	3.397	/	144.09	168.41	/	90.44	87.45	/
64	**ECOLOY**	3.404	3.351	/	89.51	160.99	/	91.86	116.65/76.14	/
65	**EHUTIM**	3.276	3.072	/	110.83	147.86	/	159.85	111.83	/
66	**EJIZEE**	3.099	3.496	/	147.82	111.31	/	134.04	93.93	/

67	**ENOFAP**	3.297	3.411	/	150.14	88.11	/	101.17	114.98	/
68	**ERORAG**	3.405	3.558	/	98.72	84.56	/	149.04	109.71	/
69	**ESIJEX**	3.112	3.545	/	165.43	93.55	/	128.65	153.43	/
70	**ESUGIK**	3.372	3.139	/	140.25	151.16	/	76.14	125.3	/
71	**ETIJEW**	3.317	3.348	/	129.16	95.31	/	155.72	157.93	/
72	**ETOPAG**	3.299	3.537	3.447	92.97	94.81	115.59	124.03	62.04	69.59
73	**ETUBEC**	3.373	3.442	/	104.23	138.4	/	127.2	66.51	/
74	**FAHXIW**	3.03	3.559	/	171.05	82.28	/	119.62/112.11	138.61	/
75	**FAPHEJ**	3.496	3.376	/	140.13	154.02	/	132.85	131.32	/
76	**FAYZEM**	3.39	3.335	/	81.43	150.63	/	150.5	156.59	/
77	**FECDID**	3.517	3.517	/	156.28	156.28	/	111.37	111.37	/
78	**FERLIA**	3.565	3.209	/	101.86	146.7	/	168.88	116.7	/
79	**FIYFEZ**	3.487	3.334	/	125.52	155.99	/	123.19/106.77	110.72/103.69	/
80	**FOCDEH**	3.504	3.493	/	108.54	146.41	/	74.05	93.64	/
81	**FOLSEH**	3.406	3.48	/	168.41	124.03	/	160.09	142.49	/
82	**FUFNUR**	3.29	3.564	3.469	158.88	114.56	139.69	101	93.41	136.91
83	**FUFVEI**	3.276	3.455	/	166.41	132.04	/	86.63	124.97	/
84	**FUHVOU**	3.569	3.273	/	160.16	160.02	/	75.54	88.38	/
85	**FUJWAJ**	3.542	3.224	/	100.24	77.92	/	170.86	106.66	/
86	**GAHPAJ**	3.21	3.253	3.546	78.08	131.26	104.14	146.38	160.04	100.82
87	**GAQZEE**	3.358	3.391	/	159.77	150.68	/	152.2	104.13	/
88	**GEGCOL**	3.383	3.2	/	122.49	162.13	/	148.48	126.51	/
89	**GEJZEC**	3.534	3.463	/	84.06	140.07	/	103.26	88.14/93.99	/
90	**GENBIL**	3.161	3.446	/	169.72	136	/	126.29/114.4	73.66	/
91	**GEPJIW**	3.18	3.266	/	163.81	140.55	/	139.35	146.16	/
92	**GIDLUB**	3.556	3.277	/	78.02	145.77	/	124.96	143.96	/
93	**GILMAN**	3.398	3.55	/	93.84	73.77	/	98.71	75.93	/
94	**GIQGUL**	3.284	3.532	/	111.21	83.34	/	143.02/97.95	96.71	/
95	**GODJER**	3.4	3.36	/	132.29	109.54	/	102.39	165.72	/
96	**GRISFL**	3.403	3.473	/	146.74	127.66	/	150.58/82.49	111.09/74.12	/
97	**GUDJAS**	3.349	2.98	/	126.54	171.19	/	92.68	105.64	/
98	**GUGVEL**	3.529	3.512	/	83.51	140.24	/	155.67/86.62	93.04/144.35	/
99	**GUHXEM**	3.165	3.39	/	115.89	92.56	/	125.85	105.75	/
100	**GUHXOW**	3.558	3.438	/	127.9	108.33	/	100.85	132.84/108.67	/
101	**HEGGIL**	3.548	3.323	/	109.53	96.06	/	71.79/102.02	159.25	/
102	**HETWEJ**	3.368	3.158	/	105.5	150.74	/	110.04/132.43	107.8	/
103	**HIJZUV**	3.522	3.484	/	77.4	146.4	/	120.48	141.26	/

104	**HOFCAJ**	3.48	3.567	/	151.98	87.13	/	149.63	89.6	/
105	**HOSMEJ**	3.324	3.303	/	101.64	167.57	/	85.91	111.19	/
106	**HOWHOS**	3.499	3.548	/	87.3	80.28	/	80.21/109.74	108.13/93.72	/
107	**HUFDES**	3.08	3.37	/	133.53	90	/	173.07	166.97	/
108	**HUGDOD**	3.237	3.38	3.243	111.01	138.36	170.76	107.9	169.49	176
109	**HUGNAZ**	3.372	3.38	/	173.37	117.63	/	100.87	109.91	/
110	**HUJMAB**	3.449	2.922	/	122.83	155.53	/	135.71/92.44	102.4/103.07/104.16	/
111	**IGABEY**	3.236	3.542	/	113.05	153.76	/	128.39	95.41	/
112	**IJOCIT**	3.254	3.18	/	155.91	136.63	/	104.73/114.02	103.32/94.89	/
113	**IKEYED**	3.435	3.464	3.31	127.5	94.11	166.42	77.9	77.45	159.34
114	**IMAKEN**	3.434	3.44	/	144.33	106.62	/	72.54	71.94/100.79	/
115	**INAWEB**	3.441	3.563	/	103.43	77.99	/	111.1	90.92	/
116	**IROREM**	3.331	3.564	/	132.73	75.38	/	109.46	92.67	/
117	**JAWBIT**	3.334	3.324	/	127.2	162.7	/	94.71/124.16	98.11	/
118	**JAZBIY**	3.216	3.231	/	140	163.62	/	143.42/96.27	139.75	/
119	**JIPCAP**	3.554	3.04	/	132.81	163.69	/	100.93/72.24	115.73/96.36	/
120	**JIRDAS**	3.307	3.521	/	69.82	98.17	/	82.09	99.79	/
121	**JOXWEA**	3.548	3.548	/	155.29	87.91	/	94.39/73.05	94.25	/
122	**JUHHAV**	3.461	3.433	/	118.15	112.74	/	89.58	90.72	/
123	**KAXFOH**	3.476	3.339	/	113.72	97.31	/	73.49	165.35	/
124	**KEFTOF**	3.058	3.48	/	167.79	93.15	/	90.43	72.3	/
125	**KERWIQ**	3.048	3.551	/	73.14	159.79	/	136.03/88.04	144.57	/
126	**KILBEM**	3.526	3.519	/	163.26	102.22	/	90.16	140.31	/
127	**KIMSIK**	3.277<br>3.386	2.953<br>3.563	/	135.71<br>81.49	162.67<br>76.07	/	120.31/119.04<br>99.61	120.31<br>89.14	/
128	**KIPHEZ**	3.354	3.416	/	76.7	88.87	/	141.4	137.86	/
129	**KIRKON**	3.065	3.568	/	173.77	91.6	/	170.14	146.5	/
130	**LAHZIE**	2.993	3.511	/	175.62	110.92	/	97.79/75.73	78.76	/
131	**LATLID**	3.127	3.325	/	163.42	118.64	/	113.94	102.84	/
132	**LEJDOV**	3.419	3.439	/	84.74	118.31	/	82.84	94.8	/
133	**LEWKII**	3.454	2.978	/	117.87	141.98	/	167.75	141.14	/
134	**LEWKUU**	3.298<br>3.449	3.332<br>3.28	/	113.55<br>145.62	65.16<br>82.54	/	136.01<br>156.45	134.11<br>127.41	/
135	**LIPVAH**	3.426	3.327	/	85.98	161.24	/	102.3	102.5	/
136	**LONVUI**	3.293	3.374	/	130.95	97.45	/	84.63	125.47	/
137	**LORWIZ**	3.504	3.222	/	80.02	160.99	/	98.98	150.52	/
138	**LUDJAW**	3.102	3.57	/	154.76	132.84	/	132.02	92.03	/

139	**MAKMIX**	3.427	3.32	/	98.61	115.3	/	105.35	153.22	/
140	**MAVZER**	3.268	3.238	/	89.64	129.57	/	73.85	74.93/90.8	/
141	**MCHTEP03**	3.458	3.538	/	102.45	165.96	/	73.62/95.48	159.98/82.25	/
142	**MEBKEM**	3.276	3.435	/	106.14	144.8	/	123.32	145.14	/
143	**MIXHIL**	3.409	3.543	/	148.8	89.11	/	77.25/90.67	80.03	/
144	**MOLLIJ**	3.21	3.47	/	167.2	69.87	/	137.43	103.95	/
145	**MOYPUN**	3.468	3.499	/	75.14	116.93	/	166.49	93.83	/
146	**MUJHIK**	3.404	3.255	/	133.98	169.23	/	114.69	160.66	/
147	**MUPSUN**	3.569	3.337	/	119.13	168.27	/	100.47	123.9/81.84	/
148	**NAMRAX**	3.48	3.047	/	85.02	152.23	/	144.42	127.45	/
149	**NARJAU**	3.467	3.159	/	97.36	147.8	/	88.99	87.17	/
150	**NATCUH**	3.565	3.347	/	90.28	161.43	/	105.27	128.37	/
151	**NEYSAN**	3.333	3.389	/	69.15	73.85	/	64.09/93.55	89.65	/
152	**NIHGOB**	3.192	3.401	/	107.46	89.33	/	167.4	106.54	/
153	**NIKFIY**	3.553	3.489	/	139.57	74.32	/	124.07	108.5	/
154	**NIPQAG**	3.044	3.399	/	162.86	75.06	/	138.75	93.79	/
155	**NITMOS**	3.428	3.459	/	177.74	123.48	/	84.36	87.1	/
156	**NIZTAS**	3.286	3.53	/	144.55	118.68	/	94.4	93.92	/
157	**NUQHOX**	3.515	3.509	/	93.29	118.63	/	83.5	119.94	/
158	**OCEKOY**	3.137	3.526	/	152.59	129.96	/	121.21/119.58	134.5	/
159	**OGUKOS**	3.279	3.405	/	130.38	145.93	/	83.34/131.73	94.08	/
160	**OKUCAZ**	3.334	3.405	/	165.71	104.46	/	106.06	156.87/95.58	/
161	**OLECAL**	3.137	3.564	/	171.35	126.05	/	127.78	159.51	/
162	**OTIMAH**	3.315	3.288	/	84.8	161.72	/	97.19/97.24	100.8/114.81	/
163	**OTOWOK**	3.443	3.277	/	151.1	157.25	/	153.74	109.74	/
164	**OWETAN**	3.242	3.068	/	159.6	155.12	/	95.01	102.6	/
165	**OZOMUM**	3.454	3.344	/	132.07	167.64	/	136.51/101.53	97.81	/
166	**PABHEF**	3.254	3.463	/	173.65	140.3	/	102.58	95.06	/
167	**PCMALA01**	3.121	3.458	/	155.62	111.4	/	172.94	168.79	/
168	**PEPPEH**	3.555	3.489	/	159.46	161.72	/	70.24	93.93/72.78	/
169	**PEXLUC**	3.094	3.525	/	156.33	141.8	/	146.58	101.59	/
170	**PICCIQ**	3.125	3.442	/	172.12	88.94	/	98.2	111.89/75.69	/
171	**PIDLAR**	2.929<br>3.477	3.487<br>3.543	/	172.04<br>177.75	83.94<br>82.36	/	166.94<br>73.91/71.45	76.76<br>166.94	/
172	**PINMUW**	3.348	3.06	/	153.8	141.57	/	129.74	146.89/94.04	/
173	**POCGEW**	3.553	3.265	/	94.76	148.05	/	84.31	139.69/100.21	/
174	**POFLOO**	3.283	3.315	/	154.85	106.99	/	86.8/89.35	95.71/82.17	/

175	**PUWPEE**	3.287	3.431	/	93.08	124.09	/	178.36	91.38	/
176	**QAHFOX**	3.504	3.436	/	75.6	96.03	/	106.83	164.63	/
177	**QAMQOL**	3.404	3.329	/	80.61	166.89	/	93.87	127.78/112.66	/
178	**QIYJOA**	3.534	3.474	/	137.51	101.17	/	88.15/75.19	77.08	/
179	**QOCRIM**	3.103	3.392	/	159.58	87.25	/	91.66	93.39	/
180	**QUFZEW**	3.491	3.552	/	116.04	92.7	/	79.89	78.7	/
181	**QUWMEC**	3.352	3.492	/	139.92	83.93	/	129.27/110.1	92.9	/
182	**RASTAG**	3.127	3.274	/	176.52	110.59	/	121.64	133.8	/
183	**RAVMUW**	3.505	3.499	/	122.34	85.04	/	123.72	79.62	/
184	**REBCEI**	2.965	3.522	/	175.14	89.98	/	100.82/140.65	76.35	/
185	**RIMTEP**	3.537	3.546	/	121.84	72.54	/	151.69	83.72	/
186	**RIMTIT**	3.554	3.558	/	121.35	73.14	/	151.66	83.45	/
187	**RIPQAK**	3.484	3.186	/	116.81	168.53	/	116.13	169.83	/
188	**RODPEH**	3.424	3.496	/	125.28	93.14	/	101.67	151.14	/
189	**ROMJOT**	3.495	3.495	/	166.18	123	/	73.92	110.44/75.27	/
190	**RUGROB**	3.415	3.197	/	90.15	155.16	/	98.88	116.86	/
191	**SABJEL**	3.399	3.455	/	91.51	94.41	/	127.73/91.78	89.7	/
192	**SAGRIF**	3.124	3.259	/	160.82	96.31	/	125.07	154.29	/
193	**SANZOY**	3.02	3.512	/	151.47	98.44	/	123.6/120.33	115.95	/
194	**SASXER**	3.564	3.314	/	86.71	178.14	/	110.29/116.86	110.14/115.42	/
195	**SAZQOZ**	3.487	3.505	/	112.39	75.19	/	119.22	85.39/83.69	/
196	**SEDQAV**	3.034	3.425	/	148.62	124.75	/	143.49	76.51	/
197	**SEGFUG**	3.315	3.479	/	90.46	78.93	/	140.71	94.19	/
198	**SEGJEV**	3.304	3.556	/	170.63	151.09	/	84.25	73.38	/
199	**SEVCAZ**	3.465	3.429	/	90.81	107.81	/	149.42/90.54	93.23/142.54	/
200	**SEVMOY**	3.557	3.496	/	74.25	93.01	/	73.88	76.56	/
201	**SIMTEO**	3.398	3.236	3.55	145.25	123.21	71.49	150.79	125.14	101.67
202	**SIQLAH**	3.219	3.427	/	147.12	120.57	/	109.12	121.18	/
203	**SUBROY**	3.376	3.479	/	78.61	133.57	/	124.09	120.67	/
204	**SUBSAL**	3.196	3.383	/	154.12	77.15	/	89.48	88.75	/
205	**SULREX**	3.292	3.475	/	112.46	77.83	/	129.85	108.28	/
206	**TAQSEL**	3.372	3.477	/	108.87	150.69	/	116.3	106.54	/
207	**TCLPHA**	3.236	3.39	/	160.47	79.47	/	116.08	139.65	/
208	**TEXCOR**	3.374	3.465	/	131.18	160.68	/	74.27	70.83	/
209	**TICMAT**	3.024	3.555	/	174.87	92.68	/	139.04	99.76	/
210	**TIGXEN**	3.485	2.927	/	69.98	172.77	/	71.42	154.93	/
211	**TIKCOF**	3.108	3.516	/	138.2	100.92	/	81.95	64.09	/

212	**TITQOF**	3.343	3.489	/	125.08	165.4	/	112.03	107.08/90.85	/
213	**TUNVIH**	3.485	3.228	/	88.91	107.27	/	81.48	96.85	/
214	**UGUQIY**	3.392	3.159	/	95.57	138.15	/	85.3	168.25	/
215	**UHUCOQ**	3.472	3.182	/	177.26	126.47	/	120.21	145.5	/
216	**UPEVER**	3.229	3.309	3.456	152.5	140.04	83.21	119.64/87.77	85.36	71.88
217	**USAMIM**	3.521	3.524	/	130.11	88.21	/	84.85	129.79/98.18	/
218	**USUQEF**	3.403	3.409	/	99.7	129.07	/	111.61/126.64	105.67	/
219	**UWAJAF**	3.08	3.487	/	166.84	137.11	/	98.58	79.45	/
220	**UZUPEL**	3.417	3.489	/	68.37	67.79	/	89.21	55.24	/
221	**VADGUCO01**	3.134	3.108	/	137.71	158.61	/	142.1/98.63	134.8	/
222	**VAFQAW**	3.401	3.17	/	155.4	127.57	/	91.82	97.23	/
223	**VECTOP**	3.544	3.463	/	123.37	102.12	/	133.47	134.06	/
224	**VECVEH**	3.498	3.55	3.431	88.48	162.74	72.68	156.23	81.4	171.62
225	**VEXDIO**	3.539	3.383	/	71.89	160.25	/	72.5/89.16	136.53	/
226	**VIFGIC**	3.388	3.47	/	166.51	94.37	/	106.65	84.42	/
227	**VIMHOQ**	3.492	3.36	/	127.1	171.12	/	111.62	81.19	/
228	**VIZNUP**	3.513	3.406	/	98.68	82.2	/	116.52	114.96	/
229	**VOJRUJ**	3.345	3.347	3.506	158.07	99.38	97.17	111.06	144.29	147.45
230	**WAHTUW**	3.53	3.29	/	134.27	150.31	/	71.02/104.69	149.92/87.53	/
231	**WEDBUE**	3.468	3.292	/	117.01	159.43	/	103.32	112.99	/
232	**WIHTEN**	3.564	3.489	/	125.47	177.8	/	102.5/80.45	103.14/70.75	/
233	**WIPGAF**	3.549	3.386	/	122.53	150.2	/	121.18	108.95/84.7	/
234	**WODLAF**	3.405	3.32	/	74.18	156.93	/	87.05	89.53	/
235	**WOGXUM**	3.503	3.48	/	87.78	98.23	/	75.53	85.61	/
236	**XAHGUL**	3.521	3.564	/	94.22	66.81	/	144.11	118.72/84.6	/
237	**XAPGUS**	3.36	3.462	/	79.46	144.08	/	145.37	90.48	/
238	**XAQGED**	3.37	3.143	/	140.16	151.14	/	76.1	125.12	/
239	**XETNES**	3.504	3.226	3.441	85.21	173.7	108.26	164.7	91.61/84.94	87.21/79.2
240	**XILDUT**	3.469	3.474	3.487	68.5	77.17	65.43	124.02/58.05	98.97/95.82	57.82/118.8
241	**XILFAB**	3.493	3.317	/	91.37	68.74	/	109.18/81.25	116.45/65.54	/
242	**XILYAT**	3.539	3.522	/	109.64	97.79	/	89.66	130.0/84.97	/
243	**XIMMUC**	2.864	3.437	/	171.58	88.17	/	125.67	93.32	/
244		3.141	3.403	/	165.2	83.04		132.29	101.01	
245	**XOMJUE**	3.465	3.477	/	154.83	124.58	/	97.66	127.03	/
246	**YASMOX**	3.264	3.293	/	165.05	133.27	/	94.87	106.57	/
247	**YEGVAI**	3.336	3.522	/	154.29	90.54	/	119.1	160.02	/

248	**YESHUA**	3.267	3.554	/	152.95	67.15	/	98.35	132.26	/
249	**YEVNIZ**	3.437	3.496	/	78.34	155.07	/	145.18	119.7	/
250	**YINVUO**	3.213	3.511	/	136.18	170.21	/	104.76	88.06	/
251	**YOPWUV**	3.391	3.531	/	73.77	77.24	/	123.71	125.44	/
252	**ZANVIV**	3.436	3.331	/	159.36	69.57	/	108.2	163.8	/
253	**ZIBWUE**	3.487	3.517	/	114.8	136.36	/	102.81/71.92	71.08	/
254	**ZIVKIY**	3.507	3.347	/	133.78	89.6	/	92.05	94.05	/
255	**QUFQUG**	3.559	3.356	/	138.86	131.01	/	82.66	91.67/83.67	/

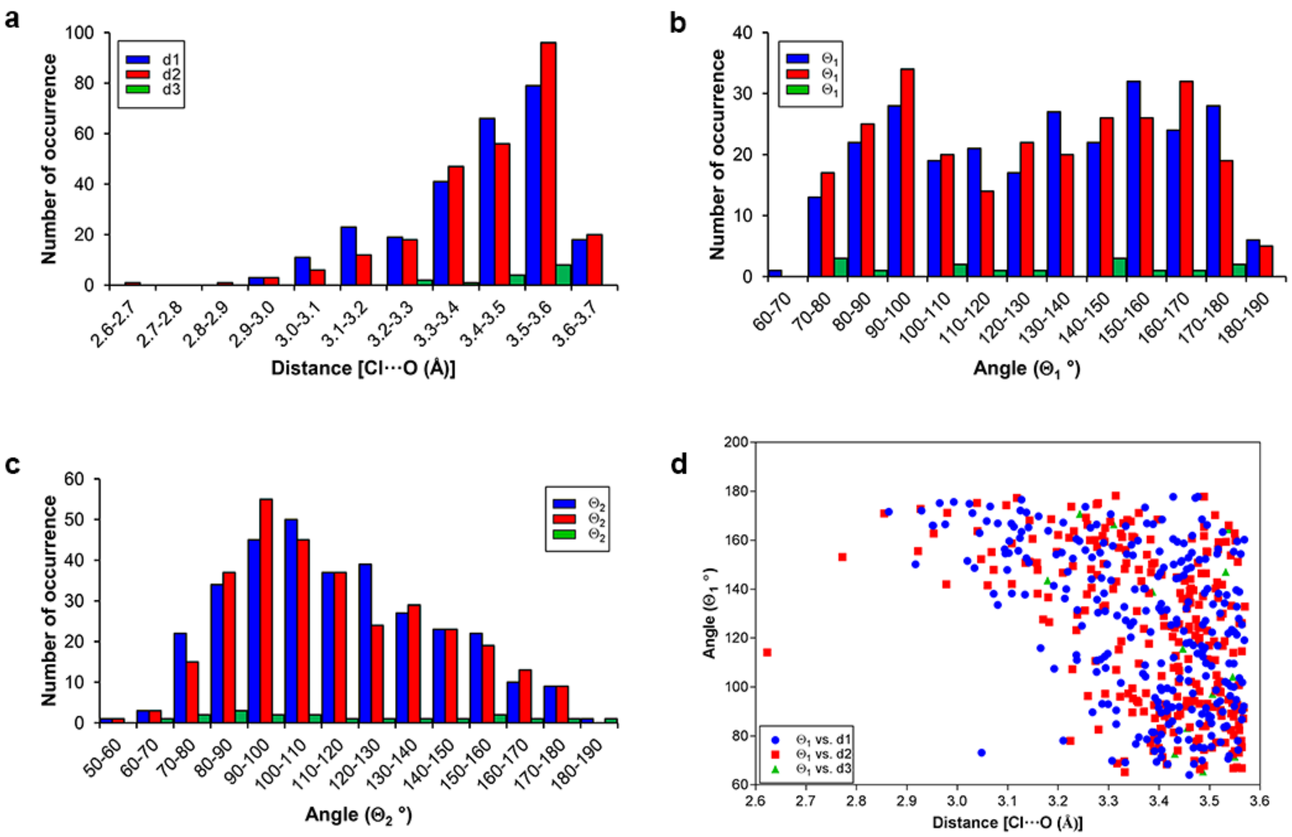

Supplementary Figure 19: Statistics on geometrical properties of bifurcated Cl···O halogen bond based on sum of van der Waals radii + 0.3 Å. a Distribution plot of bifurcated Cl···O halogen distances. **b** Distribution plot of Θ_1 [C-Cl···O] angles. **c** Distribution plot of Θ_2 [Cl···O-R] angles. **d** Scatter plots (angle (Θ_1) versus distance) obtained from the halogen bond CSD search. Distance between halogen atom and first oxygen, and associated Θ_1, Θ_2 angles are represented as blue bars, while distance between halogen atom and second oxygen, and associated Θ_1, Θ_2 angles are represented as red bars. In some cases halogen forms bonds with three oxygens and their distance and associated Θ_1, Θ_2 angles are represented as green bars.

Supplementary Table 11. Summary of hits from CSD search, forming bifurcated Br···O bonds at the distance lower than the sum of van der Waals radii.

No	Crystal structure identifier	d_1 [Å]'	d_2 [Å]''	Θ_1 ° [C-X···O]'	Θ_1 ° [C-X···O]''	Θ_2 ° [X···O-R]'	Θ_2 ° [X···O-R]''
1	**FOTFIF**	3.32	3.332	165.24	156.01	99.15	98.61
2	**HAMXAW**	3.227	3.341	164.06	142.97	122.94/101.95	126.33
3	**HOKBAN**	3.352	2.965	80.11	172.06	132.75	144.25
4	**JARHUG**	3.302	3.292	132.47	163.19	91.11/95.44	88.84
5	**JEMTIF**	3.271	3.37	138.42	134.8	127.87	112.12
6	**JIWCUO**	3.308	3.268	149.21	152.4	96.11/123.01	144.17
7	**MIJDOA**	3.145	3.369	150.35	158.04	128.72/119.93	119.85
8	**VESROC**	3.229	3.124	126.83	165.25	140.38	143.22
9	**XAKQOQ**	3.33	3.368	147.07	147.58	132.93/111.33	109.77/141.64
10	**FOTFIF**	3.32	3.332	165.24	156.01	99.15	98.61

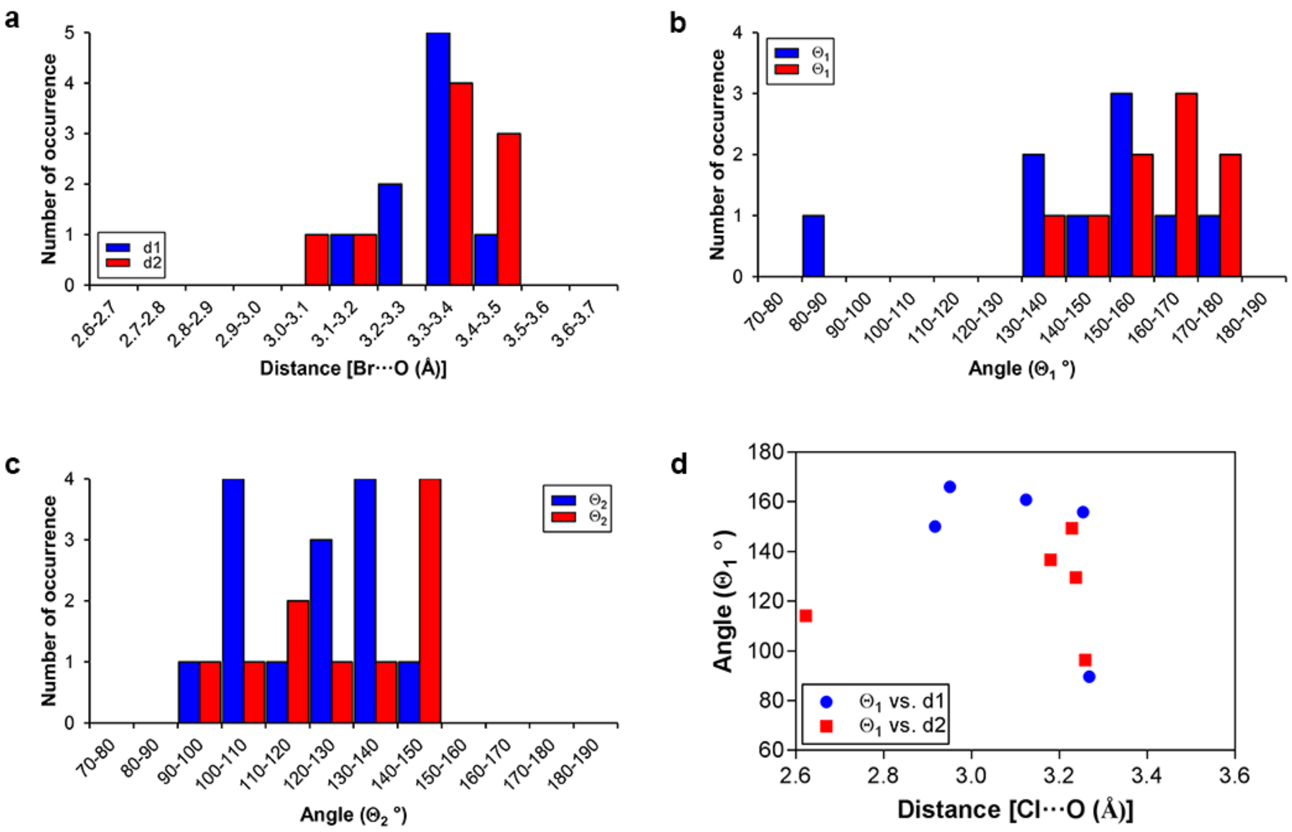

Supplementary Figure 20: Statistics on geometrical properties of bifurcated Br···O halogen bond based on sum of van der Waals radii. a Distribution plot of bifurcated Br···O halogen distances. **b** Distribution plot of Θ_1 [C-Br···O] angles. **c** Distribution plot of Θ_2 [Br···O-R] angles. **d** Scatter plots (angle (Θ_1) versus distance) obtained from the halogen bond CSD search. Distance between halogen atom and first oxygen, and associated Θ_1, Θ_2 angles are represented as blue bars, while distance between halogen atom and second oxygen, and associated Θ_1, Θ_2 angles are represented as red bars.

Supplementary Table 12. Summary of hits from CSD search, forming bifurcated I···O bonds at the distance lower than the sum of van der Waals radii.

No	Crystal structure identifier	d_1 [Å]’	d_2 [Å]’’	Θ_1 ° [C-X···O]’	Θ_1 ° [C-X···O]’’	Θ_2 ° [X···O-R]’	Θ_2 ° [X···O-R]’’
1	**NORQOD**	3.443	2.886	146.05	172.57	79.47	105.42
2	**AGAHAT**	3.027	3.311	155.39	148.89	147.73	142.85
3	**COZGII**	3.473	3.134	154.32	168.29	90.74	107.76
4	**EXIFEX**	3.214	3.214	155.14	155.14	126.11	126.11
5	**EYUPUL01**	3.456	3.437	140.36	141.57	147.09	147.09
6	**FOTDUP**	3.308	3.457	168.77	153.67	104.08	97.33
7	**IWEVUC**	3.107	3.371	159.55	160.42	135.23/103.82	91.21
8	**KIJQUR**	3.138	3.138	127.71	127.71	130.22	130.22
9	**NERXUE**	3.414	3.448	142.76	154.14	124.58	121.35
10	**QOGFUO**	3.482	3.387	142.8	162.57	141.24/94.1	93.89/104.97

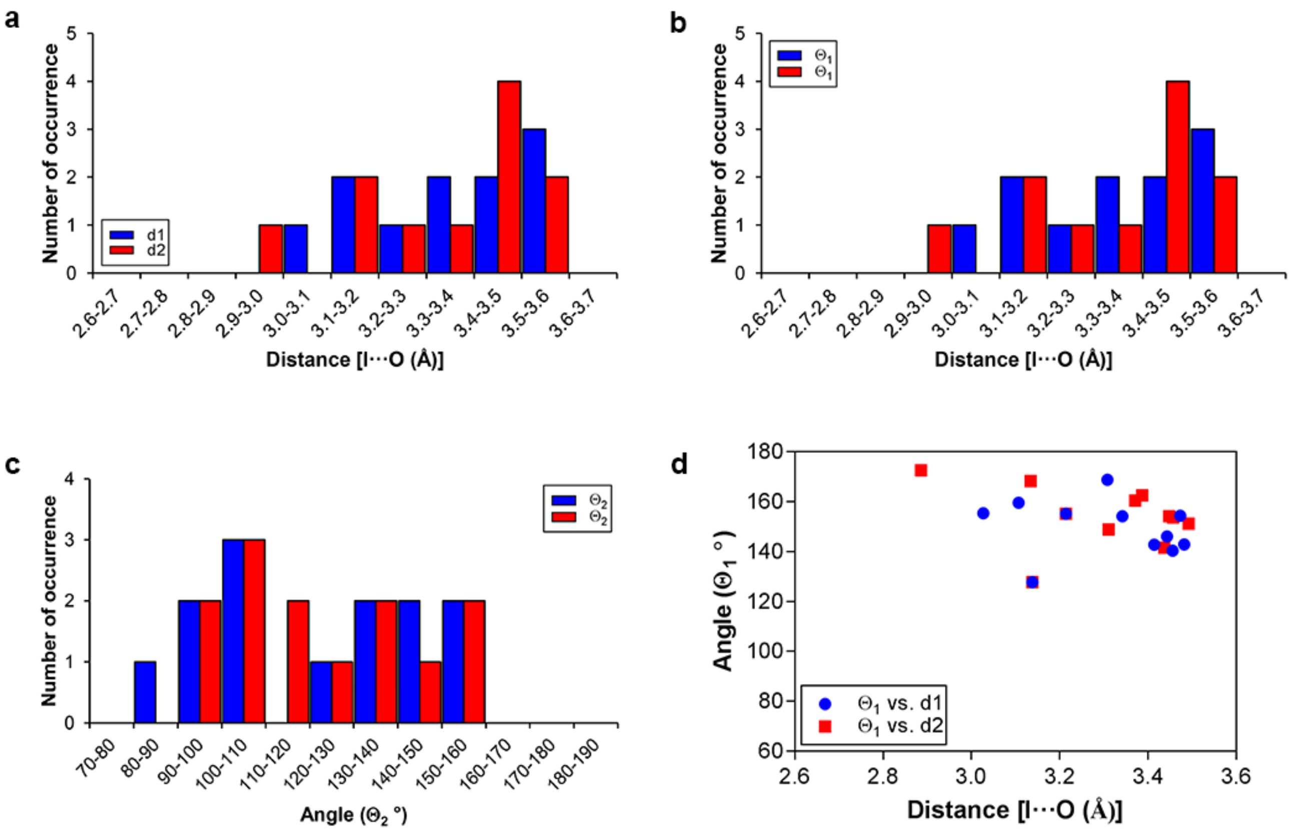

Supplementary Figure 21: Statistics on geometrical properties of bifurcated I···O halogen bond based on sum of van der Waals radii. a Distribution plot of bifurcated I···O halogen DISTANCES. **b** Distribution plot of Θ_1 [C-I···O] angles. **c** Distribution plot of Θ_2 [I···O-R] angles. **d** Scatter plots (angle (Θ_1) versus distance) obtained from the halogen bond CSD search. Distance between halogen atom and first oxygen, and associated Θ_1, Θ_2 angles are represented as blue bars, while distance between halogen atom and second oxygen, and associated Θ_1, Θ_2 angles are represented as red bars.

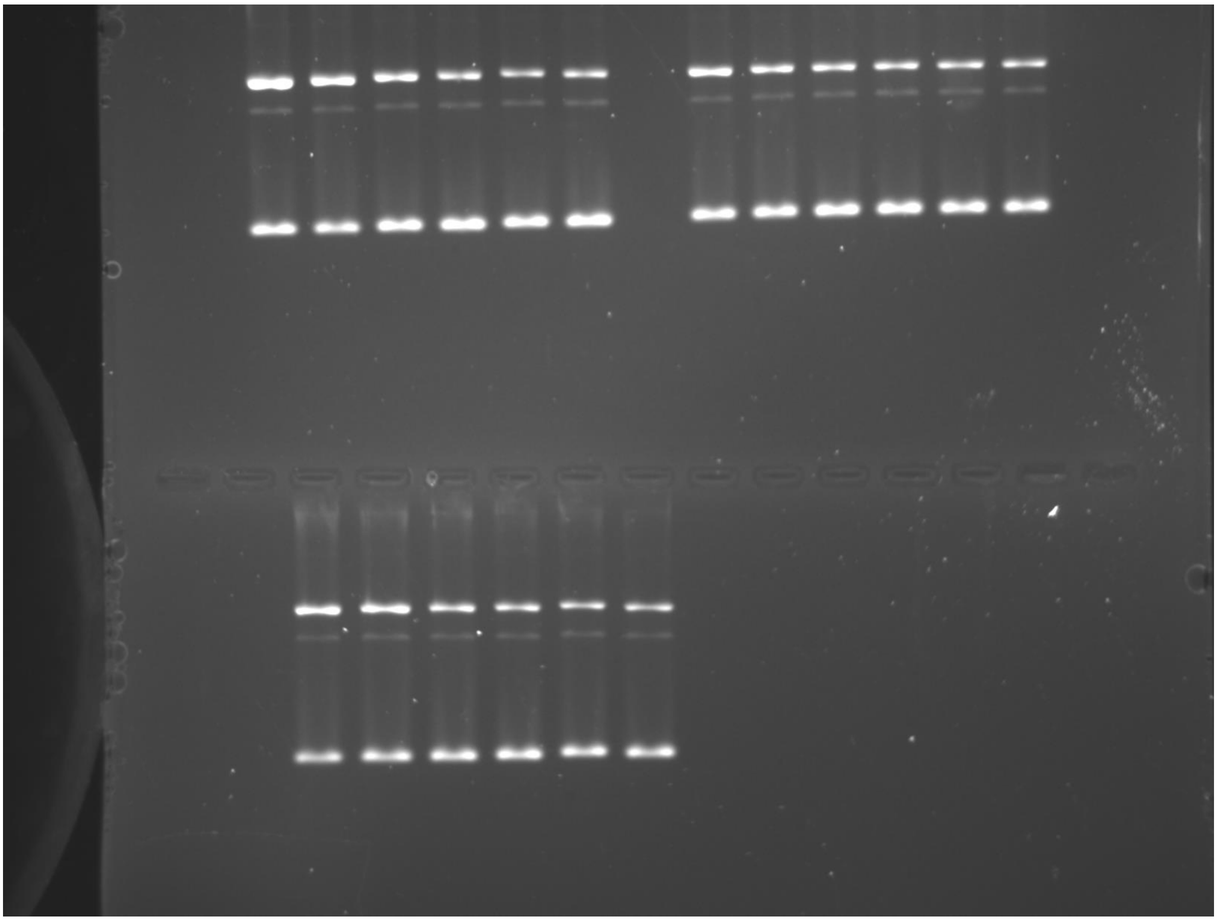

Supplementary Figure 22: Uncropped gel of the Figure 3 in the main text (supplied also as the supplementary file Dataset 2).

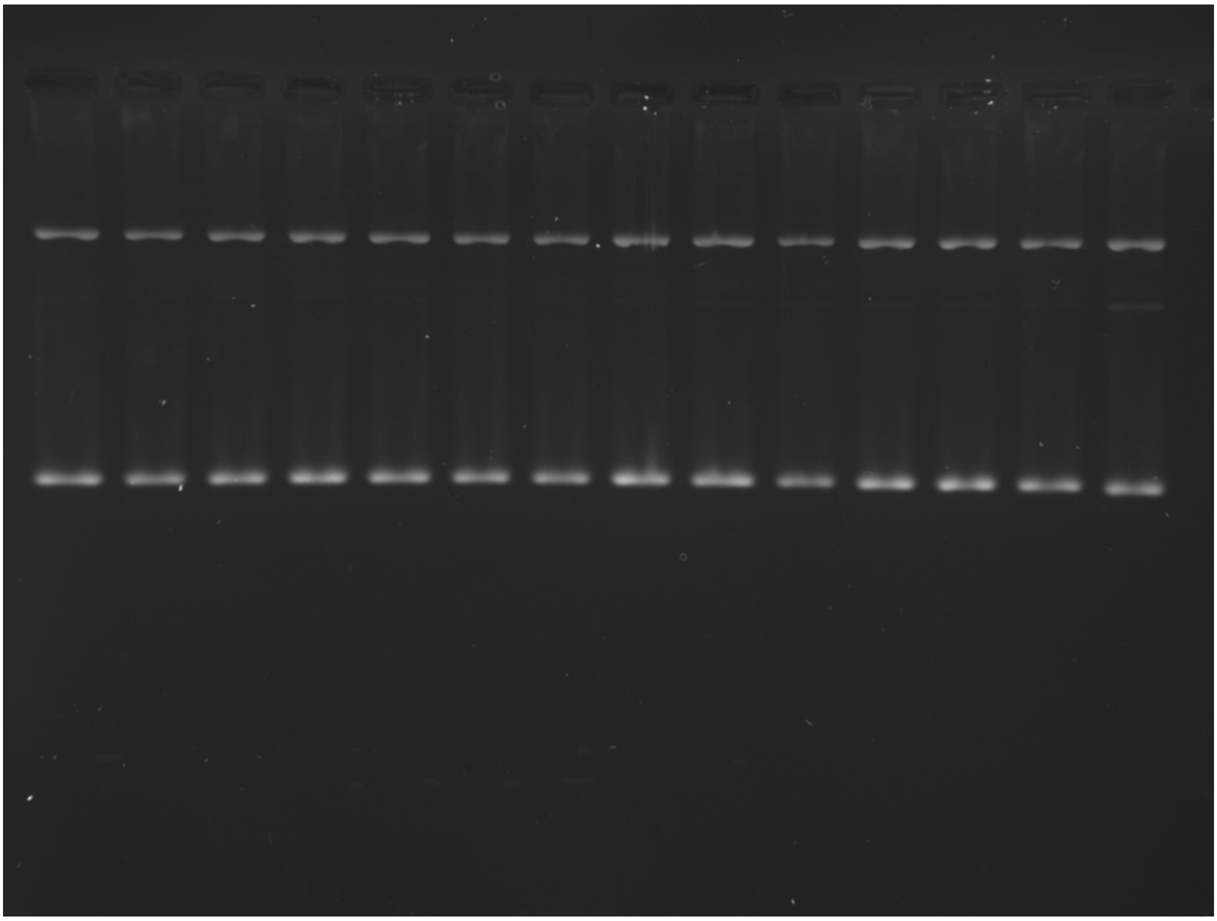

Supplementary Figure 23: Uncropped gel of the Figure 3 in the main text (no enzyme/enzyme – control, supplied also as the supplementary file Dataset 3).

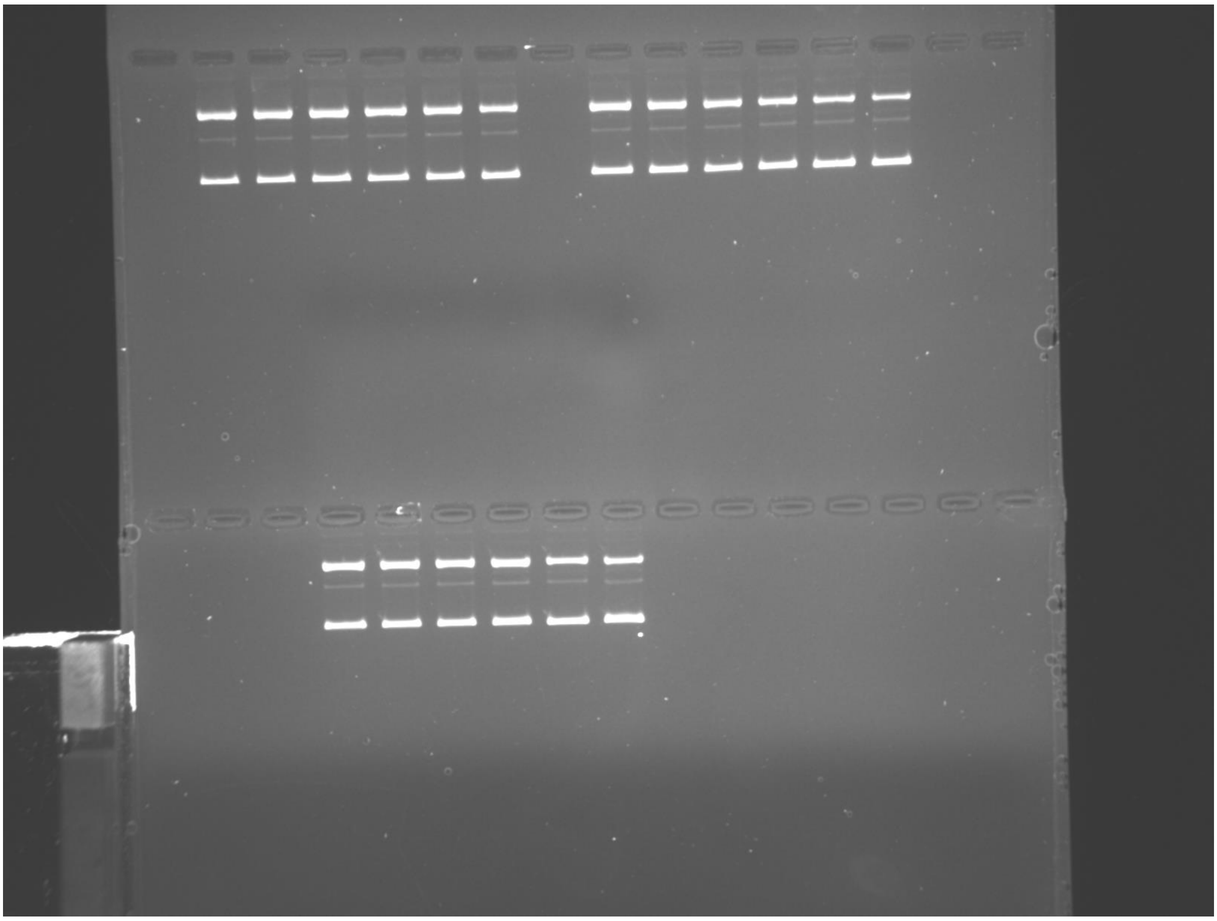

Supplementary Figure 24: Uncropped gel of the Supplementary Figure 16 (supplied also as the supplementary file Dataset 4).

References

1. Bax, B. D. et al. Type IIA topoisomerase inhibition by a new class of antibacterial agents. *Nature* **466,** 935-940 (2010).
2. Hardegger, L. A. et al. Systematic investigation of halogen bonding in protein-ligand interactions. *Angew. Chem. Int. Ed.* **50,** 314-318 (2011).
3. Politzer, P., Lane, P., Monice, C. C., Ma, Y., Murray, J. S. An overview of halogen bonding. *J. Mol. Model.* **13,** 305-311 (2007).
4. Sirimulla, S., Bailey, J. B., Vegesna, R., Narayan, M. Halogen Interactions in Protein-Ligand Complexes: Implications of Halogen Bonding for Rational Drug Design. *J. Chem. Inf. Model.* **53,** 2781-2791 (2013).
5. Montaña, Á. M. The σ and π Holes. The Halogen and Tetrel Bondings: Their Nature, Importance and Chemical, Biological and Medicinal Implications. *ChemistrySelect* **2,** 9094-9112 (2017).
6. Marvin 20.11, ChemAxon (https://www.chemaxon.com).
7. Technical bulletin. CellTiter 96® AQueous one solution cell proliferation assay 2018. Promega, USA (2001). https://worldwide.promega.com/-/media/files/resources/protocols/technical-bulletins/0/celltiter96-aqueous-one-solution-cell-proliferation-assay-system-protocol.pdf ?la=en
8. Shinada, N. K., de Brevern, A. G., Schmidtke, P. Halogens in Protein-Ligand Binding Mechanism: A Structural Perspective. *J. Med. Chem.* **62,** 9341-9356 (2019).
9. Groom, C. D., Bruno, I. J., Lightfoot M. P., Ward, S. C. The Cambridge structural database. *Acta Cryst. B* **72,** 171-179 (2016).
