# Supplementary material for: Potent DNA gyrase inhibitors bind asymmetrically to their target using symmetrical bifurcated halogen bonds
Source: Nat Commun. 2021 Jan 8;12:150. doi: 10.1038/s41467-020-20405-8 (PMC7794245; doi:10.1038/s41467-020-20405-8)
Supplement: Supplementary file 3 — Description of Additional Supplementary Files [file 41467_2020_20405_MOESM3_ESM.docx]

**Description of Additional Supplementary Files**

We have supplied the following datasets:

1. **Dataset 1** is the Full PDB X-ray Structure Validation Report, which is intended for the general audience and is described in the Description of Additional Supplementary Files.
2. **Dataset 2** is the uncropped version of the gel image of the Figure 3 in the main text and is available as Supplementary Figure 22.
3. **Dataset 3** is also the uncropped version of the gel image of the Figure 3 in the main text and is available as Supplementary Figure 22.
4. **Dataset 4** is the uncropped version of the gel image of the Supplementary Figure 16 in the Supporting Information and is available as Supplementary Figure 22.
5. **Dataset 5** is the Source Data from Molecular Dynamics simulations and is supplied as an Excel table (MD_halo_comp3-6.xlsx).
