# Supplementary material for: Potent DNA gyrase inhibitors bind asymmetrically to their target using symmetrical bifurcated halogen bonds
Source: Nat Commun. 2021 Jan 8;12:150. doi: 10.1038/s41467-020-20405-8 (PMC7794245; doi:10.1038/s41467-020-20405-8)
Supplement: Supplementary file 4 — Supplementary Dataset 1 [file 41467_2020_20405_MOESM4_ESM.pdf]

Full wwPDB X-ray Structure Validation Report (i)

May 18, 2020 – 11:25 PM BST

PDB ID	:	6Z1A
Title	:	Ternary complex of Staphylococcus aureus DNA gyrase with AMK12 and DNA
Deposited on	:	2020-05-13
Resolution	:	2.30 Å(reported)

This is a Full wwPDB X-ray Structure Validation Report.

This report is produced by the wwPDB biocuration pipeline after annotation of the structure.

We welcome your comments at *validation@mail.wwpdb.org*
A user guide is available at
https://www.wwpdb.org/validation/2017/XrayValidationReportHelp
with specific help available everywhere you see the (i) symbol.

CONFIDENTIAL VALIDATION REPORT

The following versions of software and data (see references (i)) were used in the production of this report:

MolProbity	:	4.02b-467
Mogul	:	1.8.5 (274361), CSD as541be (2020)
Xtriage (Phenix)	:	1.13
EDS	:	2.11
buster-report	:	1.1.7 (2018)
Percentile statistics	:	20171227.v01 (using entries in the PDB archive December 27th 2017)
Refmac	:	5.8.0158
CCP4	:	7.0.044 (Gargrove)
Ideal geometry (proteins)	:	Engh & Huber (2001)
Ideal geometry (DNA, RNA)	:	Parkinson et al. (1996)
Validation Pipeline (wwPDB-VP)	:	2.11

1 Overall quality at a glance (i)

The following experimental techniques were used to determine the structure:
X-RAY DIFFRACTION

The reported resolution of this entry is 2.30 Å.

Percentile scores (ranging between 0-100) for global validation metrics of the entry are shown in the following graphic. The table shows the number of entries on which the scores are based.

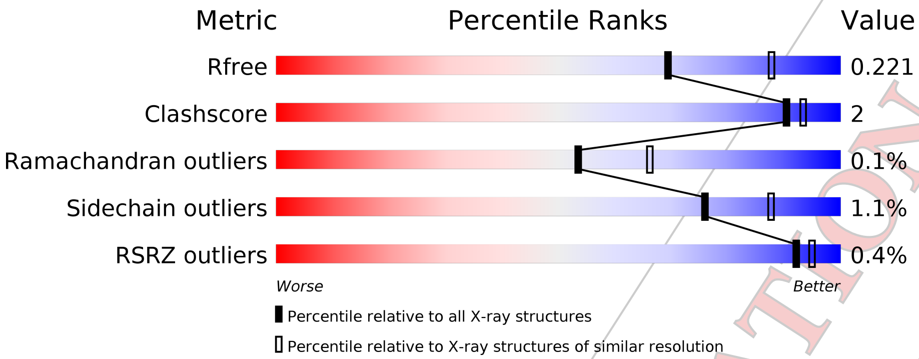

Metric	Whole archive (#Entries)	Similar resolution (#Entries, resolution range(Å))
R_{free}	111664	4477 (2.30-2.30)
Clashscore	122126	5072 (2.30-2.30)
Ramachandran outliers	120053	5022 (2.30-2.30)
Sidechain outliers	120020	5021 (2.30-2.30)
RSRZ outliers	108989	4374 (2.30-2.30)

The table below summarises the geometric issues observed across the polymeric chains and their fit to the electron density. The red, orange, yellow and green segments on the lower bar indicate the fraction of residues that contain outliers for >=3, 2, 1 and 0 types of geometric quality criteria respectively. A grey segment represents the fraction of residues that are not modelled. The numeric value for each fraction is indicated below the corresponding segment, with a dot representing fractions <=5% The upper red bar (where present) indicates the fraction of residues that have poor fit to the electron density. The numeric value is given above the bar.

Mol	Chain	Length	Quality of chain
1	B	692	% / 91% 6% •
1	D	692	91% 5% •
2	E	8	88% 13%
2	F	8	75% 25%
3	G	12	83% 17%
3	H	12	58% 42%

CONFIDENTIAL VALIDATION REPORT

2 Entry composition (i)

There are 9 unique types of molecules in this entry. The entry contains 11802 atoms, of which 0 are hydrogens and 0 are deuteriums.

In the tables below, the ZeroOcc column contains the number of atoms modelled with zero occupancy, the AltConf column contains the number of residues with at least one atom in alternate conformation and the Trace column contains the number of residues modelled with at most 2 atoms.

- Molecule 1 is a protein called DNA gyrase subunit B,DNA gyrase subunit A.

Mol	Chain	Residues	Atoms					ZeroOcc	AltConf	Trace
1	B	671	Total 5279	C 3294	N 951	O 1009	S 25	0	0	0
1	D	671	Total 5299	C 3303	N 952	O 1019	S 25	0	3	0

There are 76 discrepancies between the modelled and reference sequences:

Chain	Residue	Modelled	Actual	Comment	Reference
B	409	MET	-	initiating methionine	UNP P0A0K8
B	?	-	LEU	deletion	UNP P0A0K8
B	?	-	TYR	deletion	UNP P0A0K8
B	?	-	LYS	deletion	UNP P0A0K8
B	?	-	LEU	deletion	UNP P0A0K8
B	?	-	THR	deletion	UNP P0A0K8
B	?	-	GLN	deletion	UNP P0A0K8
B	?	-	GLY	deletion	UNP P0A0K8
B	?	-	LYS	deletion	UNP P0A0K8
B	?	-	GLN	deletion	UNP P0A0K8
B	?	-	LYS	deletion	UNP P0A0K8
B	?	-	TYR	deletion	UNP P0A0K8
B	?	-	TYR	deletion	UNP P0A0K8
B	?	-	VAL	deletion	UNP P0A0K8
B	?	-	TYR	deletion	UNP P0A0K8
B	?	-	ASN	deletion	UNP P0A0K8
B	?	-	ASP	deletion	UNP P0A0K8
B	?	-	ARG	deletion	UNP P0A0K8
B	?	-	GLU	deletion	UNP P0A0K8
B	?	-	LEU	deletion	UNP P0A0K8
B	?	-	ASP	deletion	UNP P0A0K8
B	?	-	LYS	deletion	UNP P0A0K8
B	?	-	LEU	deletion	UNP P0A0K8
B	?	-	LYS	deletion	UNP P0A0K8
B	?	-	SER	deletion	UNP P0A0K8

Continued on next page...

CONFIDENTIAL VALIDATION REPORT

Continued from previous page...

Chain	Residue	Modelled	Actual	Comment	Reference
B	?	-	GLU	deletion	UNP P0A0K8
B	?	-	LEU	deletion	UNP P0A0K8
B	?	-	ASN	deletion	UNP P0A0K8
B	?	-	PRO	deletion	UNP P0A0K8
B	?	-	THR	deletion	UNP P0A0K8
B	?	-	PRO	deletion	UNP P0A0K8
B	?	-	LYS	deletion	UNP P0A0K8
B	?	-	TRP	deletion	UNP P0A0K8
B	?	-	SER	deletion	UNP P0A0K8
B	?	-	ILE	deletion	UNP P0A0K8
B	544	THR	ALA	conflict	UNP P0A0K8
B	545	GLY	ARG	conflict	UNP P0A0K8
B	1123	PHE	TYR	engineered mutation	UNP P20831
D	409	MET	-	initiating methionine	UNP P0A0K8
D	?	-	LEU	deletion	UNP P0A0K8
D	?	-	TYR	deletion	UNP P0A0K8
D	?	-	LYS	deletion	UNP P0A0K8
D	?	-	LEU	deletion	UNP P0A0K8
D	?	-	THR	deletion	UNP P0A0K8
D	?	-	GLN	deletion	UNP P0A0K8
D	?	-	GLY	deletion	UNP P0A0K8
D	?	-	LYS	deletion	UNP P0A0K8
D	?	-	GLN	deletion	UNP P0A0K8
D	?	-	LYS	deletion	UNP P0A0K8
D	?	-	TYR	deletion	UNP P0A0K8
D	?	-	TYR	deletion	UNP P0A0K8
D	?	-	VAL	deletion	UNP P0A0K8
D	?	-	TYR	deletion	UNP P0A0K8
D	?	-	ASN	deletion	UNP P0A0K8
D	?	-	ASP	deletion	UNP P0A0K8
D	?	-	ARG	deletion	UNP P0A0K8
D	?	-	GLU	deletion	UNP P0A0K8
D	?	-	LEU	deletion	UNP P0A0K8
D	?	-	ASP	deletion	UNP P0A0K8
D	?	-	LYS	deletion	UNP P0A0K8
D	?	-	LEU	deletion	UNP P0A0K8
D	?	-	LYS	deletion	UNP P0A0K8
D	?	-	SER	deletion	UNP P0A0K8
D	?	-	GLU	deletion	UNP P0A0K8
D	?	-	LEU	deletion	UNP P0A0K8
D	?	-	ASN	deletion	UNP P0A0K8
D	?	-	PRO	deletion	UNP P0A0K8

Continued on next page...

CONFIDENTIAL VALIDATION REPORT

Continued from previous page...

Chain	Residue	Modelled	Actual	Comment	Reference
D	?	-	THR	deletion	UNP P0A0K8
D	?	-	PRO	deletion	UNP P0A0K8
D	?	-	LYS	deletion	UNP P0A0K8
D	?	-	TRP	deletion	UNP P0A0K8
D	?	-	SER	deletion	UNP P0A0K8
D	?	-	ILE	deletion	UNP P0A0K8
D	544	THR	ALA	conflict	UNP P0A0K8
D	545	GLY	ARG	conflict	UNP P0A0K8
D	1123	PHE	TYR	engineered mutation	UNP P20831

- Molecule 2 is a DNA chain called DNA (5'-D(*AP*GP*CP*CP*GP*TP*AP*G)-3').

Mol	Chain	Residues	Atoms					ZeroOcc	AltConf	Trace
2	E	8	Total 163	C 78	N 33	O 45	P 7	0	0	0
2	F	8	Total 163	C 78	N 33	O 45	P 7	0	0	0

- Molecule 3 is a DNA chain called DNA (5'-D(P*GP*TP*AP*CP*CP*TP*AP*CP*GP*GP *CP*T)-3').

Mol	Chain	Residues	Atoms					ZeroOcc	AltConf	Trace
3	G	12	Total 245	C 116	N 43	O 74	P 12	0	0	0
3	H	12	Total 230	C 107	N 41	O 70	P 12	0	0	0

- Molecule 4 is 4-(2-HYDROXYETHYL)-1-PIPERAZINE ETHANESULFONIC ACID (three-letter code: EPE) (formula: $C_8H_{18}N_2O_4S$).

CONFIDENTIAL VALIDATION REPORT

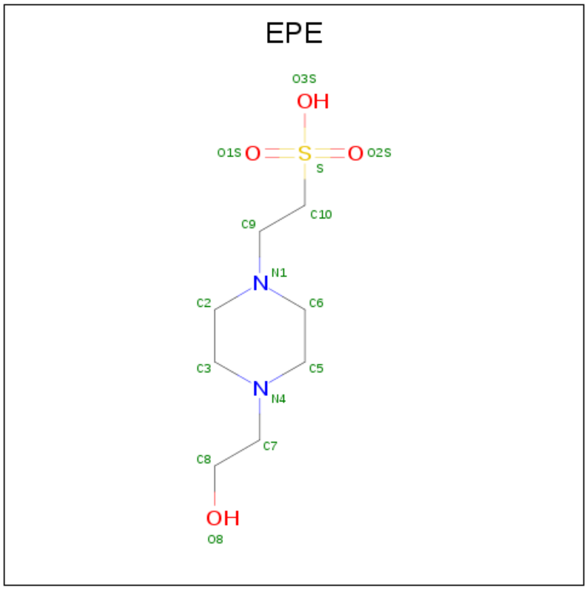

Mol	Chain	Residues	Atoms	ZeroOcc	AltConf
4	B	1	Total C N O S<br>15 8 2 4 1	0	0
4	D	1	Total C N O S<br>15 8 2 4 1	0	0

- Molecule 5 is MANGANESE (II) ION (three-letter code: MN) (formula: Mn).

Mol	Chain	Residues	Atoms	ZeroOcc	AltConf
5	G	1	Total Mn<br>1 1	0	0
5	B	1	Total Mn<br>1 1	0	0
5	D	2	Total Mn<br>2 2	0	0

- Molecule 6 is DIMETHYL SULFOXIDE (three-letter code: DMS) (formula: C_2H_6OS).

CONFIDENTIAL VALIDATION REPORT

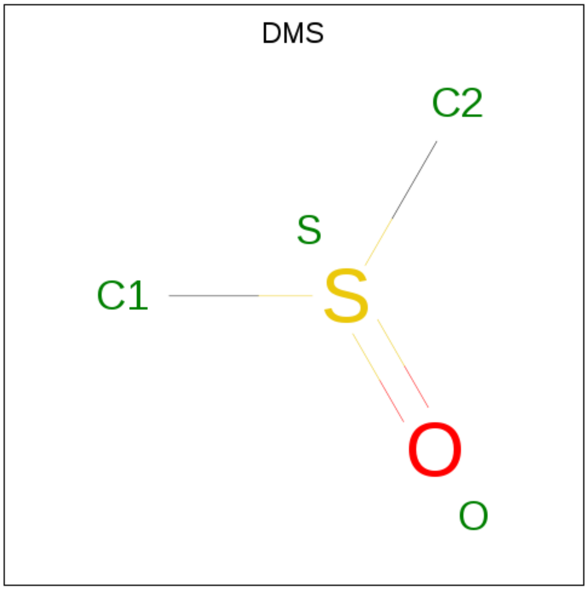

Mol	Chain	Residues	Atoms	ZeroOcc	AltConf
6	D	1	Total C O S<br>4 2 1 1	0	0
6	D	1	Total C O S<br>4 2 1 1	0	0
6	E	1	Total C O S<br>4 2 1 1	0	0

- Molecule 7 is CHLORIDE ION (three-letter code: CL) (formula: Cl).

Mol	Chain	Residues	Atoms	ZeroOcc	AltConf
7	D	1	Total Cl<br>1 1	0	0

- Molecule 8 is {N}-[(4-chlorophenyl)methyl]-1-[2-(6-methoxy-1,5-naphthyridin-4-yl)ethyl] piperidin-4-amine (three-letter code: Q52) (formula: $C_{23}H_{27}ClN_4O$) (labeled as "Ligand of Interest" by author).

CONFIDENTIAL VALIDATION REPORT

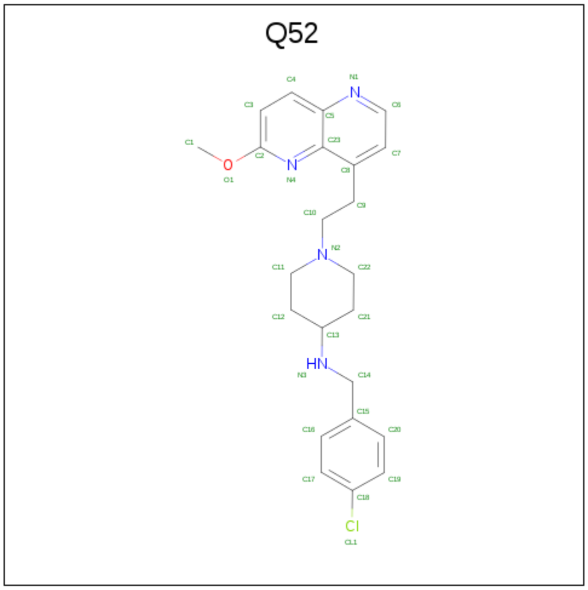

Mol	Chain	Residues	Atoms	ZeroOcc	AltConf
8	G	1	Total C Cl N O<br>29 23 1 4 1	0	0

- Molecule 9 is water.

Mol	Chain	Residues	Atoms	ZeroOcc	AltConf
9	B	153	Total O<br>155 155	0	2
9	D	158	Total O<br>160 160	0	2
9	E	9	Total O<br>9 9	0	0
9	F	8	Total O<br>8 8	0	0
9	G	8	Total O<br>8 8	0	0
9	H	7	Total O<br>7 7	0	0

CONFIDENTIAL VALIDATION REPORT

3 Residue-property plots (i)

These plots are drawn for all protein, RNA and DNA chains in the entry. The first graphic for a chain summarises the proportions of the various outlier classes displayed in the second graphic. The second graphic shows the sequence view annotated by issues in geometry and electron density. Residues are color-coded according to the number of geometric quality criteria for which they contain at least one outlier: green = 0, yellow = 1, orange = 2 and red = 3 or more. A red dot above a residue indicates a poor fit to the electron density (RSRZ > 2). Stretches of 2 or more consecutive residues without any outlier are shown as a green connector. Residues present in the sample, but not in the model, are shown in grey.

- Molecule 1: DNA gyrase subunit B,DNA gyrase subunit A

Chain B:

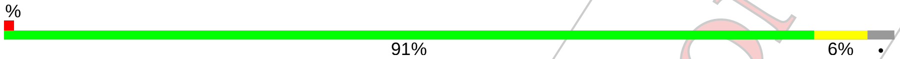

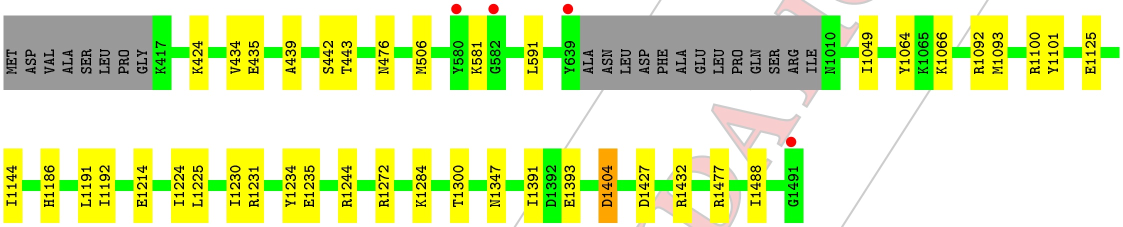

- Molecule 1: DNA gyrase subunit B,DNA gyrase subunit A

Chain D:

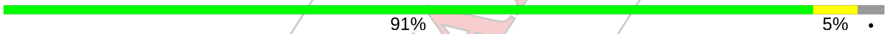

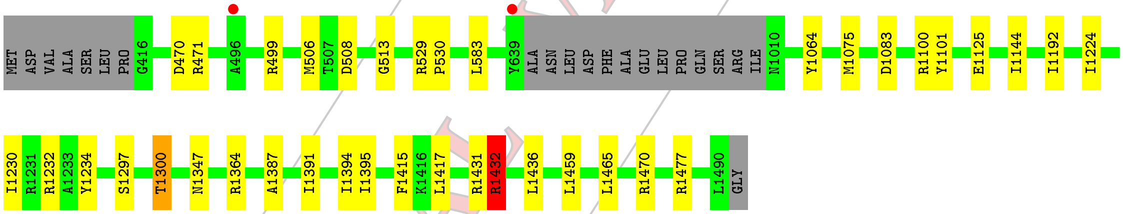

- Molecule 2: DNA (5'-D(*AP*GP*CP*CP*GP*TP*AP*G)-3')

Chain E:

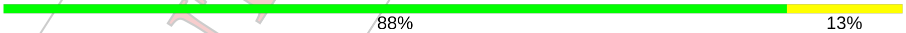

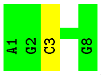

- Molecule 2: DNA (5'-D(*AP*GP*CP*CP*GP*TP*AP*G)-3')

Chain F:

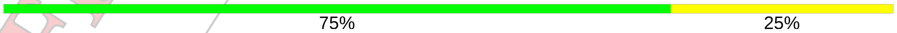

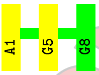

- Molecule 3: DNA (5'-D(P*GP*TP*AP*CP*CP*TP*AP*CP*GP*GP*CP*T)-3')

CONFIDENTIAL VALIDATION REPORT

Chain G:

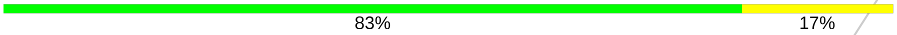

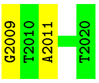

- Molecule 3: DNA (5'-D(P*GP*TP*AP*CP*CP*TP*AP*CP*GP*GP*CP*T)-3')

Chain H:

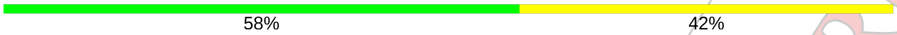

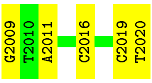

CONFIDENTIAL VALIDATION REPORT

4 Data and refinement statistics (i)

Property	Value	Source
Space group	P 61	Depositor
Cell constants<br>a, b, c, α, β, γ	92.59Å 92.59Å 405.46Å<br>90.00° 90.00° 120.00°	Depositor
Resolution (Å)	80.32 – 2.30<br>80.19 – 2.30	Depositor<br>EDS
% Data completeness<br>(in resolution range)	99.9 (80.32-2.30)<br>100.0 (80.19-2.30)	Depositor<br>EDS
R_{merge}	0.19	Depositor
R_{sym}	(Not available)	Depositor
$< I/\sigma(I) >$ [1]	1.59 (at 2.29Å)	Xtriage
Refinement program	REFMAC 5.8.0258	Depositor
R, R_{free}	0.179 , 0.217<br>0.185 , 0.221	Depositor<br>DCC
R_{free} test set	4483 reflections (5.17%)	wwPDB-VP
Wilson B-factor (Å^2)	43.3	Xtriage
Anisotropy	0.207	Xtriage
Bulk solvent k_{sol}(e/Å^3), B_{sol}(Å^2)	0.34 , 43.0	EDS
L-test for twinning[2]	$< \|L\| >$ = 0.47, $< L^2 >$ = 0.30	Xtriage
Estimated twinning fraction	0.104 for h,-h-k,-l	Xtriage
F_o,F_c correlation	0.95	EDS
Total number of atoms	11802	wwPDB-VP
Average B, all atoms (Å^2)	49.0	wwPDB-VP

Xtriage's analysis on translational NCS is as follows: *The largest off-origin peak in the Patterson function is 3.12% of the height of the origin peak. No significant pseudotranslation is detected.*

[1] Intensities estimated from amplitudes.
[2] Theoretical values of $< |L| >$, $< L^2 >$ for acentric reflections are 0.5, 0.333 respectively for untwinned datasets, and 0.375, 0.2 for perfectly twinned datasets.

CONFIDENTIAL VALIDATION REPORT

5 Model quality (i)

5.1 Standard geometry (i)

Bond lengths and bond angles in the following residue types are not validated in this section: EPE, Q52, DMS, MN, CL

The Z score for a bond length (or angle) is the number of standard deviations the observed value is removed from the expected value. A bond length (or angle) with $|Z| > 5$ is considered an outlier worth inspection. RMSZ is the root-mean-square of all Z scores of the bond lengths (or angles).

Mol	Chain	Bond lengths		Bond angles	
		RMSZ	#$\|Z\|$ >5	RMSZ	#$\|Z\|$ >5
1	B	0.75	0/5351	0.89	2/7213 (0.0%)
1	D	0.74	0/5371	0.90	6/7244 (0.1%)
2	E	0.83	0/183	0.98	0/281
2	F	0.85	1/183 (0.5%)	1.25	1/281 (0.4%)
3	G	0.93	1/273 (0.4%)	1.24	2/417 (0.5%)
3	H	0.95	1/256 (0.4%)	1.26	4/391 (1.0%)
All	All	0.76	3/11617 (0.0%)	0.93	15/15827 (0.1%)

All (3) bond length outliers are listed below:

Mol	Chain	Res	Type	Atoms	Z	Observed(Å)	Ideal(Å)
3	H	2009	DG	OP3-P	-9.51	1.49	1.61
3	G	2009	DG	OP3-P	-6.44	1.53	1.61
2	F	5	DG	O3'-P	-5.17	1.54	1.61

All (15) bond angle outliers are listed below:

Mol	Chain	Res	Type	Atoms	Z	Observed(°)	Ideal(°)
3	G	2011	DA	O5'-P-OP1	-12.24	94.69	105.70
3	H	2020	DT	O5'-P-OP1	-11.01	95.79	105.70
2	F	5	DG	O5'-P-OP2	-8.90	97.69	105.70
3	H	2020	DT	O5'-P-OP2	7.64	119.87	110.70
1	B	1092	ARG	NE-CZ-NH1	-6.95	116.83	120.30
1	D	1432[A]	ARG	CG-CD-NE	6.11	124.62	111.80
1	D	1432[B]	ARG	CG-CD-NE	6.11	124.62	111.80
3	H	2019	DC	C1'-O4'-C4'	-6.08	104.02	110.10
1	D	1470	ARG	NE-CZ-NH2	-5.86	117.37	120.30
3	G	2009	DG	O5'-P-OP1	-5.52	100.73	105.70
1	B	1432	ARG	NE-CZ-NH1	5.46	123.03	120.30
1	D	1432[A]	ARG	NE-CZ-NH1	5.46	123.03	120.30

Continued on next page...

CONFIDENTIAL VALIDATION REPORT

Continued from previous page...

Mol	Chain	Res	Type	Atoms	Z	Observed(°)	Ideal(°)
1	D	1432[B]	ARG	NE-CZ-NH1	5.46	123.03	120.30
1	D	499	ARG	NE-CZ-NH2	5.30	122.95	120.30
3	H	2016	DC	C1'-O4'-C4'	-5.26	104.84	110.10

There are no chirality outliers.

There are no planarity outliers.

5.2 Too-close contacts (i)

In the following table, the Non-H and H(model) columns list the number of non-hydrogen atoms and hydrogen atoms in the chain respectively. The H(added) column lists the number of hydrogen atoms added and optimized by MolProbity. The Clashes column lists the number of clashes within the asymmetric unit, whereas Symm-Clashes lists symmetry related clashes.

Mol	Chain	Non-H	H(model)	H(added)	Clashes	Symm-Clashes
1	B	5279	0	5303	23	0
1	D	5299	0	5293	20	0
2	E	163	0	91	1	0
2	F	163	0	91	1	0
3	G	245	0	136	0	0
3	H	230	0	123	1	0
4	B	15	0	18	0	0
4	D	15	0	17	0	0
5	B	1	0	0	0	0
5	D	2	0	0	0	0
5	G	1	0	0	0	0
6	D	8	0	12	0	0
6	E	4	0	6	0	0
7	D	1	0	0	0	0
8	G	29	0	0	0	0
9	B	155	0	0	1	0
9	D	160	0	0	0	1
9	E	9	0	0	0	1
9	F	8	0	0	0	0
9	G	8	0	0	0	0
9	H	7	0	0	0	0
All	All	11802	0	11090	43	1

The all-atom clashscore is defined as the number of clashes found per 1000 atoms (including hydrogen atoms). The all-atom clashscore for this structure is 2.

All (43) close contacts within the same asymmetric unit are listed below, sorted by their clash

CONFIDENTIAL VALIDATION REPORT

magnitude.

Atom-1	Atom-2	Interatomic distance (Å)	Clash overlap (Å)
1:B:1404:ASP:OD2	1:D:1431:ARG:NH2	2.10	0.84
1:B:435:GLU:OE1	9:B:1801:HOH:O	2.06	0.74
1:D:1432[A]:ARG:HH11	1:D:1432[A]:ARG:HG3	1.60	0.64
1:D:1297:SER:OG	1:D:1300:THR:HG23	1.99	0.63
1:D:1465:LEU:HD23	1:D:1465:LEU:C	2.18	0.63
1:B:442:SER:HB3	1:B:591:LEU:HD12	1.81	0.61
2:F:1:DA:H2'	2:F:1:DA:N3	2.22	0.54
1:B:1064:TYR:HB3	1:B:1125:GLU:HB3	1.91	0.52
1:D:508:ASP:O	1:D:513:GLY:HA3	2.10	0.52
1:D:1234:TYR:O	1:D:1347:ASN:HB2	2.10	0.51
1:B:506:MET:HE1	1:B:591:LEU:HD22	1.93	0.51
1:D:1075:MET:HE3	1:D:1083:ASP:N	2.27	0.50
1:D:1100:ARG:HG3	1:D:1101:TYR:CE2	2.48	0.49
1:B:1192:ILE:HG21	1:B:1477:ARG:HB2	1.95	0.48
1:D:1192:ILE:HG21	1:D:1477:ARG:HB2	1.95	0.48
1:B:1234:TYR:O	1:B:1347:ASN:HB2	2.14	0.47
1:B:1100:ARG:HG3	1:B:1101:TYR:CE2	2.51	0.46
1:B:439:ALA:O	1:B:443:THR:HG23	2.15	0.46
1:B:1144:ILE:HD12	1:B:1144:ILE:C	2.37	0.44
1:D:1064:TYR:HB3	1:D:1125:GLU:HB3	1.99	0.44
1:B:1224:ILE:HG21	1:B:1230:ILE:HD11	1.97	0.44
1:D:1144:ILE:HD12	1:D:1144:ILE:C	2.39	0.44
1:B:1272:ARG:CZ	2:E:3:DC:H5"	2.48	0.44
1:B:1225:LEU:HD21	1:B:1244:ARG:HD2	2.00	0.43
1:B:434:VAL:HA	1:B:506:MET:O	2.17	0.43
1:D:1224:ILE:HG21	1:D:1230:ILE:HD11	1.99	0.43
1:D:529:ARG:N	1:D:530:PRO:CD	2.82	0.43
1:D:1391:ILE:O	1:D:1395:ILE:HG12	2.17	0.43
1:D:506:MET:HG2	1:D:583:LEU:HD11	1.99	0.43
1:B:1214:GLU:HB3	1:B:1488:ILE:HD12	2.00	0.42
1:B:1144:ILE:HD12	1:B:1144:ILE:O	2.19	0.42
1:D:1364:ARG:HB2	1:D:1465:LEU:HD11	2.02	0.42
3:H:2011:DA:O5'	3:H:2011:DA:H2'	2.20	0.41
1:B:1049:ILE:HG21	1:B:1093:MET:HE1	2.02	0.41
1:D:1415:PHE:HB2	1:D:1417:LEU:HG	2.02	0.41
1:B:1049:ILE:CG2	1:B:1093:MET:HE1	2.51	0.41
1:B:476:ASN:H	1:B:476:ASN:ND2	2.18	0.41
1:B:1391:ILE:HA	1:B:1391:ILE:HD12	1.93	0.41
1:B:1231:ARG:O	1:B:1235:GLU:HB2	2.21	0.41
1:B:1427:ASP:OD1	1:D:1431:ARG:NH1	2.50	0.40
1:D:1387:ALA:HA	1:D:1394:ILE:HG13	2.02	0.40

Continued on next page...

CONFIDENTIAL VALIDATION REPORT

Continued from previous page...

Atom-1	Atom-2	Interatomic distance (Å)	Clash overlap (Å)
1:D:1459:LEU:HD23	1:D:1459:LEU:HA	1.95	0.40
1:B:1186:HIS:HB2	1:B:1191:LEU:HD11	2.03	0.40

All (1) symmetry-related close contacts are listed below. The label for Atom-2 includes the symmetry operator and encoded unit-cell translations to be applied.

Atom-1	Atom-2	Interatomic distance (Å)	Clash overlap (Å)
9:D:1853:HOH:O	9:E:1702:HOH:O[1_445]	2.12	0.08

5.3 Torsion angles (i)

5.3.1 Protein backbone (i)

In the following table, the Percentiles column shows the percent Ramachandran outliers of the chain as a percentile score with respect to all X-ray entries followed by that with respect to entries of similar resolution.

The Analysed column shows the number of residues for which the backbone conformation was analysed, and the total number of residues.

Mol	Chain	Analysed	Favoured	Allowed	Outliers	Percentiles	
1	B	667/692 (96%)	648 (97%)	18 (3%)	1 (0%)	53	66
1	D	670/692 (97%)	650 (97%)	20 (3%)	0	100	100
All	All	1337/1384 (97%)	1298 (97%)	38 (3%)	1 (0%)	53	66

All (1) Ramachandran outliers are listed below:

Mol	Chain	Res	Type
1	B	581	LYS

5.3.2 Protein sidechains (i)

In the following table, the Percentiles column shows the percent sidechain outliers of the chain as a percentile score with respect to all X-ray entries followed by that with respect to entries of similar resolution.

The Analysed column shows the number of residues for which the sidechain conformation was analysed, and the total number of residues.

CONFIDENTIAL VALIDATION REPORT

Mol	Chain	Analysed	Rotameric	Outliers	Percentiles	
1	B	564/590 (96%)	558 (99%)	6 (1%)	76	87
1	D	565/590 (96%)	558 (99%)	7 (1%)	74	86
All	All	1129/1180 (96%)	1116 (99%)	13 (1%)	76	86

All (13) residues with a non-rotameric sidechain are listed below:

Mol	Chain	Res	Type
1	B	424	LYS
1	B	1066	LYS
1	B	1284	LYS
1	B	1300	THR
1	B	1393	GLU
1	B	1404	ASP
1	D	470	ASP
1	D	471	ARG
1	D	1232	ARG
1	D	1300	THR
1	D	1432[A]	ARG
1	D	1432[B]	ARG
1	D	1436	LEU

Some sidechains can be flipped to improve hydrogen bonding and reduce clashes. All (2) such sidechains are listed below:

Mol	Chain	Res	Type
1	B	476	ASN
1	B	1107	GLN

5.3.3 RNA (i)

There are no RNA molecules in this entry.

5.4 Non-standard residues in protein, DNA, RNA chains (i)

There are no non-standard protein/DNA/RNA residues in this entry.

5.5 Carbohydrates (i)

There are no carbohydrates in this entry.

CONFIDENTIAL VALIDATION REPORT

5.6 Ligand geometry ⓘ

Of 11 ligands modelled in this entry, 5 are monoatomic - leaving 6 for Mogul analysis.

In the following table, the Counts columns list the number of bonds (or angles) for which Mogul statistics could be retrieved, the number of bonds (or angles) that are observed in the model and the number of bonds (or angles) that are defined in the Chemical Component Dictionary. The Link column lists molecule types, if any, to which the group is linked. The Z score for a bond length (or angle) is the number of standard deviations the observed value is removed from the expected value. A bond length (or angle) with $|Z| > 2$ is considered an outlier worth inspection. RMSZ is the root-mean-square of all Z scores of the bond lengths (or angles).

Mol	Type	Chain	Res	Link	Bond lengths			Bond angles		
					Counts	RMSZ	$\#\|Z\| > 2$	Counts	RMSZ	$\#\|Z\| > 2$
6	DMS	E	1601	-	3,3,3	0.29	0	3,3,3	0.09	0
6	DMS	D	1602	-	3,3,3	0.33	0	3,3,3	0.17	0
4	EPE	D	1603	-	15,15,15	1.01	1 (6%)	18,20,20	1.22	2 (11%)
6	DMS	D	1601	-	3,3,3	0.27	0	3,3,3	0.09	0
4	EPE	B	1701	-	15,15,15	0.71	1 (6%)	18,20,20	0.81	1 (5%)
8	Q52	G	2101	-	32,32,32	0.44	0	42,43,43	0.65	0

In the following table, the Chirals column lists the number of chiral outliers, the number of chiral centers analysed, the number of these observed in the model and the number defined in the Chemical Component Dictionary. Similar counts are reported in the Torsion and Rings columns. '-' means no outliers of that kind were identified.

Mol	Type	Chain	Res	Link	Chirals	Torsions	Rings
8	Q52	G	2101	-	-	3/12/22/22	0/4/4/4
4	EPE	D	1603	-	-	2/9/19/19	0/1/1/1
4	EPE	B	1701	-	-	2/9/19/19	0/1/1/1

All (2) bond length outliers are listed below:

Mol	Chain	Res	Type	Atoms	Z	Observed(Å)	Ideal(Å)
4	D	1603	EPE	O1S-S	3.64	1.55	1.45
4	B	1701	EPE	O3S-S	2.59	1.56	1.47

All (3) bond angle outliers are listed below:

Mol	Chain	Res	Type	Atoms	Z	Observed(^{o})	Ideal(^{o})
4	D	1603	EPE	O1S-S-C10	-3.71	102.44	106.92
4	D	1603	EPE	O3S-S-O2S	2.67	117.79	111.27
4	B	1701	EPE	O3S-S-C10	-2.27	102.09	105.77

CONFIDENTIAL VALIDATION REPORT

There are no chirality outliers.

All (7) torsion outliers are listed below:

Mol	Chain	Res	Type	Atoms
8	G	2101	Q52	C9-C10-N2-C11
8	G	2101	Q52	C9-C10-N2-C22
4	B	1701	EPE	N4-C7-C8-O8
4	D	1603	EPE	C9-C10-S-O3S
4	D	1603	EPE	C9-C10-S-O1S
4	B	1701	EPE	C9-C10-S-O1S
8	G	2101	Q52	C15-C14-N3-C13

There are no ring outliers.

No monomer is involved in short contacts.

The following is a two-dimensional graphical depiction of Mogul quality analysis of bond lengths, bond angles, torsion angles, and ring geometry for all instances of the Ligand of Interest. In addition, ligands with molecular weight > 250 and outliers as shown on the validation Tables will also be included. For torsion angles, if less then 5% of the Mogul distribution of torsion angles is within 10 degrees of the torsion angle in question, then that torsion angle is considered an outlier. Any bond that is central to one or more torsion angles identified as an outlier by Mogul will be highlighted in the graph. For rings, the root-mean-square deviation (RMSD) between the ring in question and similar rings identified by Mogul is calculated over all ring torsion angles. If the average RMSD is greater than 60 degrees and the minimal RMSD between the ring in question and any Mogul-identified rings is also greater than 60 degrees, then that ring is considered an outlier. The outliers are highlighted in purple. The color gray indicates Mogul did not find sufficient equivalents in the CSD to analyse the geometry.

Ligand Q52 G 2101

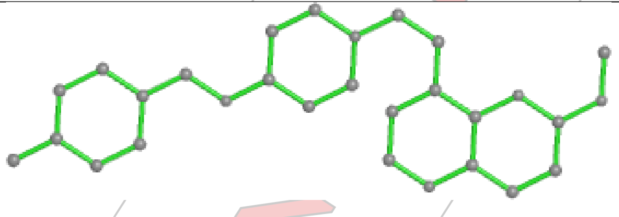

Bond lengths

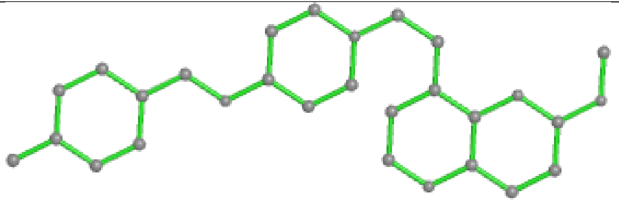

Bond angles

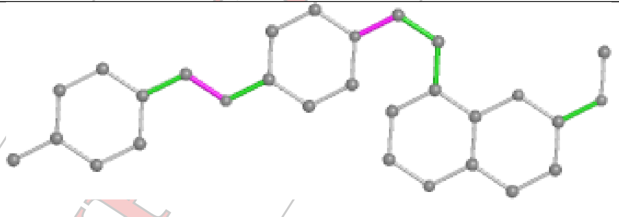

Torsions

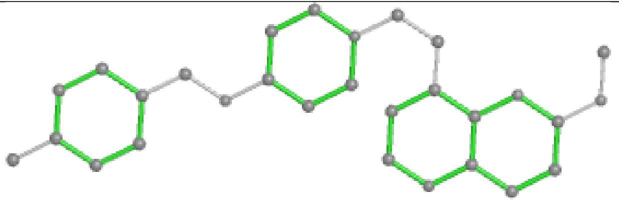

Rings

CONFIDENTIAL VALIDATION REPORT

5.7 Other polymers (i)

There are no such residues in this entry.

5.8 Polymer linkage issues (i)

There are no chain breaks in this entry.

CONFIDENTIAL VALIDATION REPORT

6 Fit of model and data (i)

6.1 Protein, DNA and RNA chains (i)

In the following table, the column labelled '#RSRZ> 2' contains the number (and percentage) of RSRZ outliers, followed by percent RSRZ outliers for the chain as percentile scores relative to all X-ray entries and entries of similar resolution. The OWAB column contains the minimum, median, 95th percentile and maximum values of the occupancy-weighted average B-factor per residue. The column labelled 'Q< 0.9' lists the number of (and percentage) of residues with an average occupancy less than 0.9.

Mol	Chain	Analysed	<RSRZ>	#RSRZ>2			OWAB(Å^2)	Q<0.9
1	B	671/692 (96%)	-0.14	4 (0%)	89	92	32, 46, 69, 110	0
1	D	671/692 (96%)	-0.16	2 (0%)	93	96	33, 46, 72, 92	0
2	E	8/8 (100%)	-0.49	0	100	100	37, 43, 53, 60	0
2	F	8/8 (100%)	-0.38	0	100	100	38, 46, 75, 92	0
3	G	12/12 (100%)	-0.27	0	100	100	38, 55, 67, 74	0
3	H	12/12 (100%)	-0.24	0	100	100	35, 55, 75, 93	0
All	All	1382/1424 (97%)	-0.16	6 (0%)	92	95	32, 46, 72, 110	0

All (6) RSRZ outliers are listed below:

Mol	Chain	Res	Type	RSRZ
1	B	639	TYR	6.0
1	B	580	TYR	4.0
1	B	1491	GLY	2.5
1	D	639	TYR	2.3
1	D	496	ALA	2.1
1	B	582	GLY	2.0

6.2 Non-standard residues in protein, DNA, RNA chains (i)

There are no non-standard protein/DNA/RNA residues in this entry.

6.3 Carbohydrates (i)

There are no carbohydrates in this entry.

CONFIDENTIAL VALIDATION REPORT

6.4 Ligands ⓘ

In the following table, the Atoms column lists the number of modelled atoms in the group and the number defined in the chemical component dictionary. The B-factors column lists the minimum, median, 95th percentile and maximum values of B factors of atoms in the group. The column labelled 'Q< 0.9' lists the number of atoms with occupancy less than 0.9.

Mol	Type	Chain	Res	Atoms	RSCC	RSR	B-factors($Å^2$)	Q<0.9
7	CL	D	1604	1/1	0.84	0.11	74,74,74,74	0
6	DMS	D	1602	4/4	0.88	0.26	80,83,86,93	0
6	DMS	D	1601	4/4	0.89	0.19	92,95,105,105	0
8	Q52	G	2101	29/29	0.90	0.24	51,59,78,80	0
4	EPE	B	1701	15/15	0.92	0.26	62,83,90,91	0
4	EPE	D	1603	15/15	0.94	0.20	65,78,81,90	0
6	DMS	E	1601	4/4	0.95	0.13	76,77,83,85	0
5	MN	G	2102	1/1	0.97	0.08	65,65,65,65	0
5	MN	D	1606	1/1	0.97	0.04	66,66,66,66	0
5	MN	B	1702	1/1	0.99	0.05	51,51,51,51	0
5	MN	D	1605	1/1	0.99	0.07	48,48,48,48	0

The following is a graphical depiction of the model fit to experimental electron density of all instances of the Ligand of Interest. In addition, ligands with molecular weight > 250 and outliers as shown on the geometry validation Tables will also be included. Each fit is shown from different orientation to approximate a three-dimensional view.

Electron density around Q52 G 2101:

2mF$_o$-DF$_c$ (at 0.7 rmsd) in gray
mF$_o$-DF$_c$ (at 3 rmsd) in purple (negative)
and green (positive)

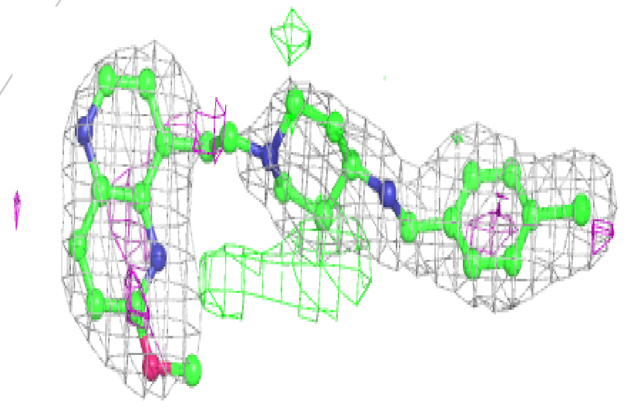

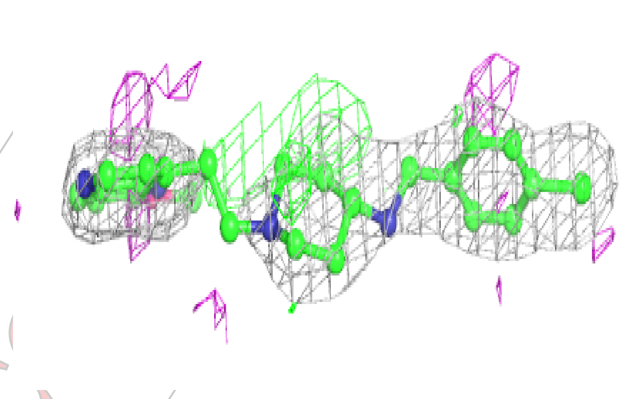

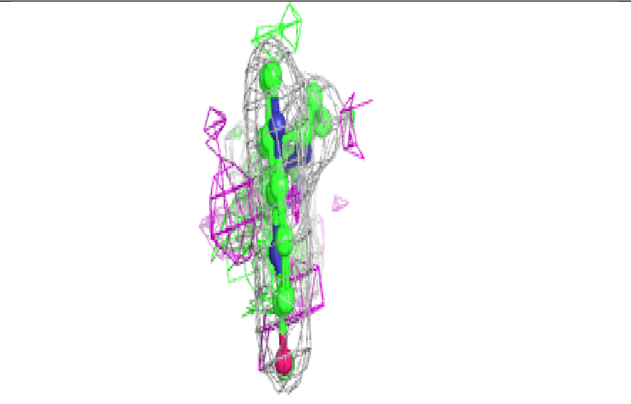

6.5 Other polymers (i)

There are no such residues in this entry.

CONFIDENTIAL VALIDATION REPORT
