# Supplementary figures and images for: Potent DNA gyrase inhibitors bind asymmetrically to their target using symmetrical bifurcated halogen bonds
Source: Nat Commun. 2021 Jan 8;12:150. doi: 10.1038/s41467-020-20405-8 (PMC7794245; doi:10.1038/s41467-020-20405-8)

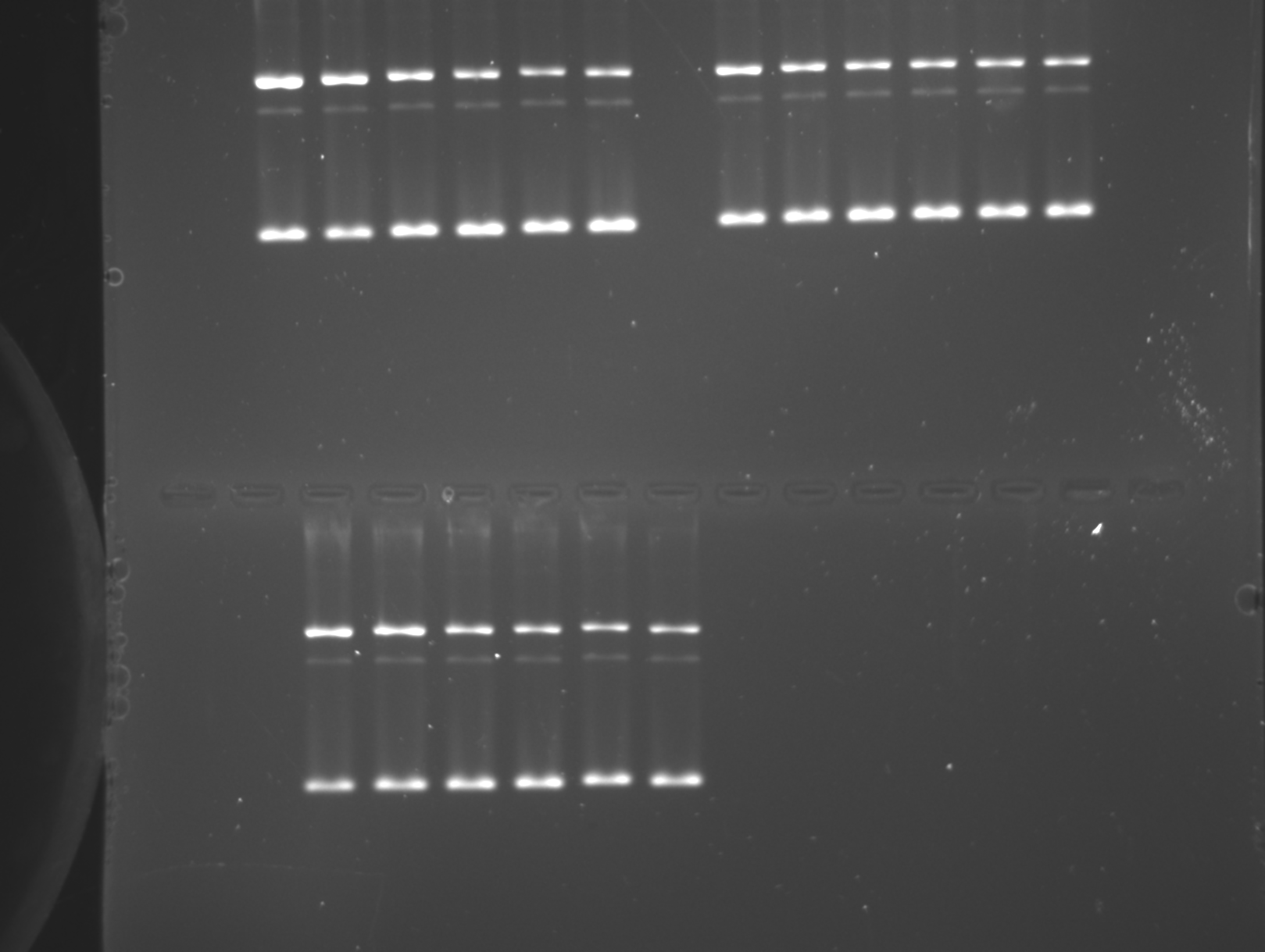

Supplement: Supplementary file 5 — Supplementary Dataset 2 [file 41467_2020_20405_MOESM5_ESM.tif]

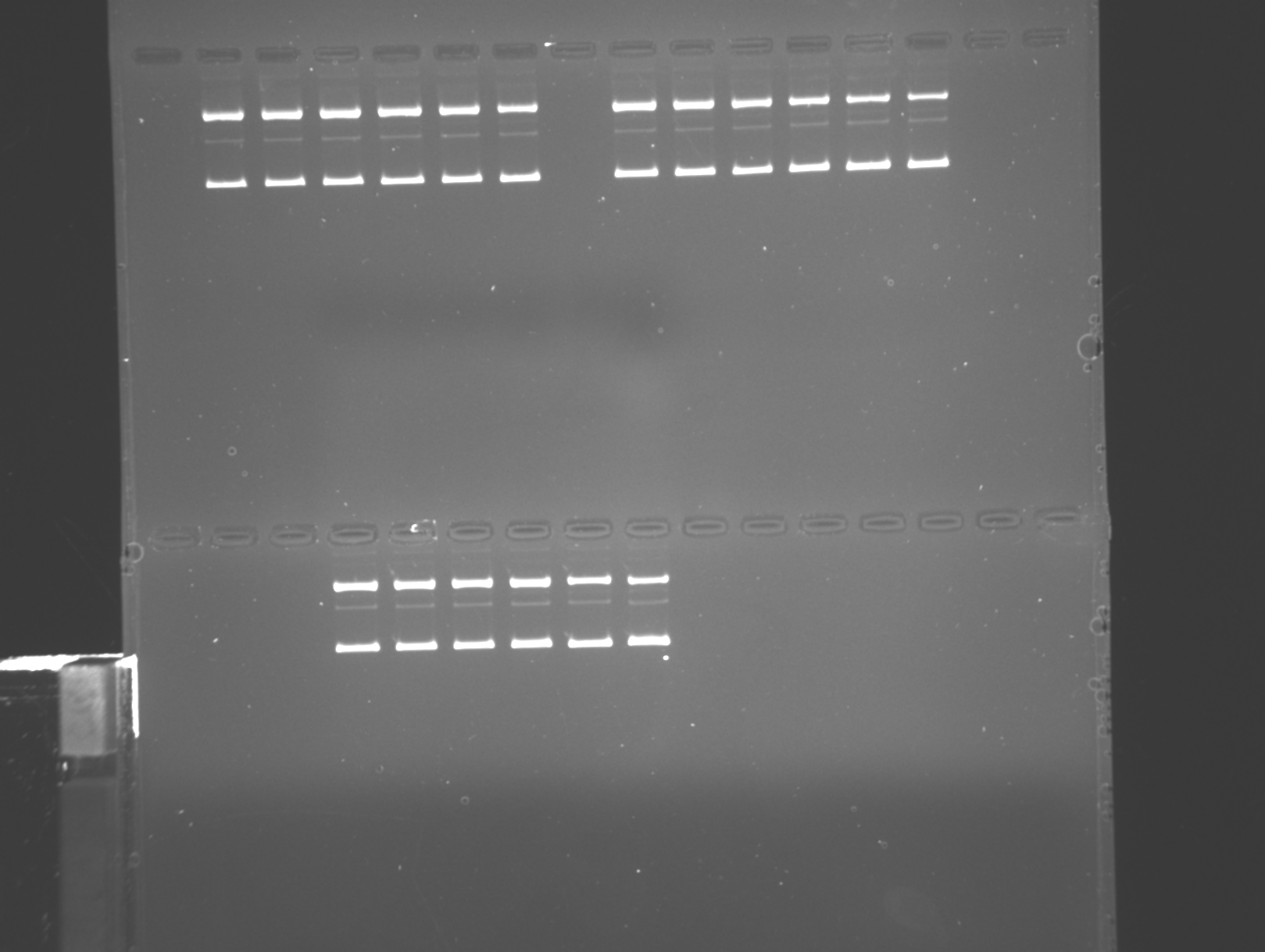

Supplement: Supplementary file 6 — Supplementary Dataset 3 [file 41467_2020_20405_MOESM6_ESM.tif]

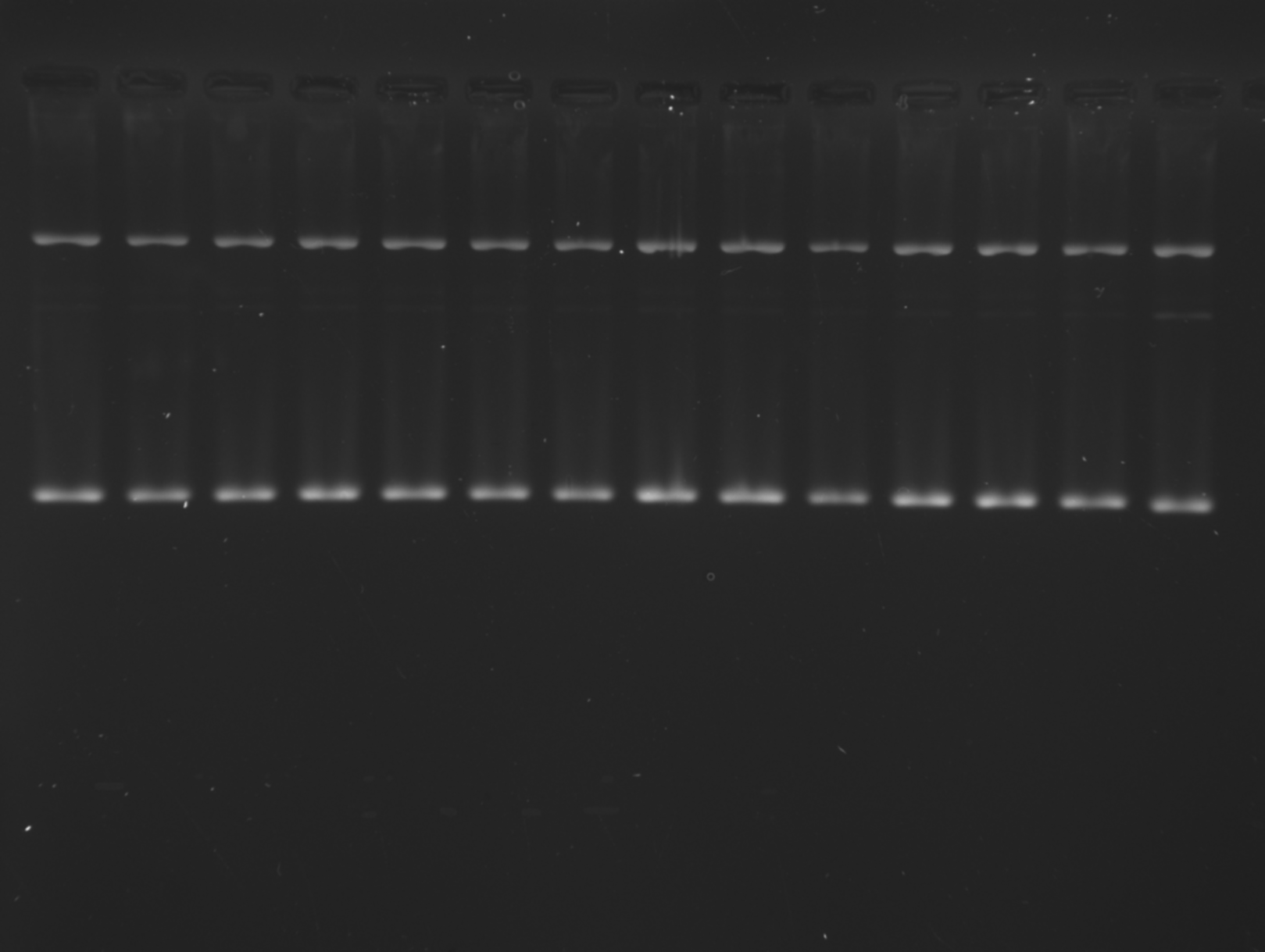

Supplement: Supplementary file 7 — Supplementary Dataset 4 [file 41467_2020_20405_MOESM7_ESM.tif]
