# Supplementary material for: Potent DNA gyrase inhibitors bind asymmetrically to their target using symmetrical bifurcated halogen bonds
Source: Nat Commun. 2021 Jan 8;12:150. doi: 10.1038/s41467-020-20405-8 (PMC7794245; doi:10.1038/s41467-020-20405-8)
Supplement: Supplementary file 9 — Reporting Summary [file 41467_2020_20405_MOESM9_ESM.pdf]

nature research

Corresponding author(s): Marko Anderluh, Nikola Minovski

Last updated by author(s): Nov 20, 2020

Reporting Summary

Nature Research wishes to improve the reproducibility of the work that we publish. This form provides structure for consistency and transparency in reporting. For further information on Nature Research policies, see our Editorial Policies and the Editorial Policy Checklist.

Statistics

For all statistical analyses, confirm that the following items are present in the figure legend, table legend, main text, or Methods section.

n/a	Confirmed	
☐	☒	The exact sample size (n) for each experimental group/condition, given as a discrete number and unit of measurement
☐	☒	A statement on whether measurements were taken from distinct samples or whether the same sample was measured repeatedly
☒	☐	The statistical test(s) used AND whether they are one- or two-sided<br>*Only common tests should be described solely by name; describe more complex techniques in the Methods section.*
☒	☐	A description of all covariates tested
☒	☐	A description of any assumptions or corrections, such as tests of normality and adjustment for multiple comparisons
☐	☒	A full description of the statistical parameters including central tendency (e.g. means) or other basic estimates (e.g. regression coefficient) AND variation (e.g. standard deviation) or associated estimates of uncertainty (e.g. confidence intervals)
☒	☐	For null hypothesis testing, the test statistic (e.g. F, t, r) with confidence intervals, effect sizes, degrees of freedom and P value noted<br>*Give P values as exact values whenever suitable.*
☒	☐	For Bayesian analysis, information on the choice of priors and Markov chain Monte Carlo settings
☒	☐	For hierarchical and complex designs, identification of the appropriate level for tests and full reporting of outcomes
☒	☐	Estimates of effect sizes (e.g. Cohen's d, Pearson's r), indicating how they were calculated

Our web collection on statistics for biologists contains articles on many of the points above.

Software and code

Policy information about availability of computer code

Data collection	Cambridge Structure Database (CSD) 2020.1
Data analysis	ChemDraw Professional 15.0.0.106, UCSF ChimeraX 0.91, GraphPad Prism 6.0 and 7.0, Pymol 2.0.4, MarvinSketch 20.17., Discovery Studio 4.1, Pipeline Pilot 9.2, LigandScout 3.12, ConQuest 2020.1.1, Mercury version 2020.1. CPPTRAJ: Trajectory Analysis. V18.01 (AmberTools V18), VMD v1.9.3, GNUPLOT v4.6, SigmaPlot 13

For manuscripts utilizing custom algorithms or software that are central to the research but not yet described in published literature, software must be made available to editors and reviewers. We strongly encourage code deposition in a community repository (e.g. GitHub). See the Nature Research guidelines for submitting code & software for further information.

Data

Policy information about availability of data

All manuscripts must include a data availability statement. This statement should provide the following information, where applicable:

- Accession codes, unique identifiers, or web links for publicly available datasets
- A list of figures that have associated raw data
- A description of any restrictions on data availability

Model coordinates and density maps are available in the Protein Data Bank (PDB ID 6Z1A, https://www.rcsb.org/structure/6Z1A). Crystal structures used within small molecule database search are publicly available in the Chambridge Structural Database repository (https://www.ccdc.cam.ac.uk/structures/?), using crystal structure identifiers listed in Supplementary information. Crystal structures used within pdb database search are publicly available in Protein Data Bank repository (PDB IDs: 2XCS (https://www.rcsb.org/structure/2XCS), 5CDP (https://www.rcsb.org/structure/5CDP), 1GJD (https://www.rcsb.org/structure/1GJD), 1UHI (https://www.rcsb.org/structure/1UHI), 4CMJ (https://www.rcsb.org/structure/4CMJ), 4JYI (https://www.rcsb.org/structure/4JYI), 5A86 (https://www.rcsb.org/structure/5A86), 5YC6 (https://www.rcsb.org/structure/5YC6), 5YC7 (https://www.rcsb.org/structure/5YC7)). The source data of Fig. 3a are provided as a Source

Data file. Other data that support the findings of this study are available from the corresponding author upon reasonable request.

Field-specific reporting

Please select the one below that is the best fit for your research. If you are not sure, read the appropriate sections before making your selection.

☒ Life sciences ☐ Behavioural & social sciences ☐ Ecological, evolutionary & environmental sciences

For a reference copy of the document with all sections, see nature.com/documents/nr-reporting-summary-flat.pdf

Life sciences study design

All studies must disclose on these points even when the disclosure is negative.

Sample size	No sample-size calculation was performed. The sample size of 3 was considered sufficient due to the low variability of these measurements when done with the same enzyme preparation. The number of biological repeats (2-4) was chosen taking into account the fundamental principles to show experiments repeatability (https://www.embopress.org/doi/full/10.1038/embor.2012.36).
Data exclusions	No data were excluded.
Replication	All assays were performed two to four times under the same conditions to verify reproducibility, which was succesfully confirmed.
Randomization	This was not relevant for our study, because we did not perform experiments that would include experimental groups.
Blinding	This was not relevant for our study, because we did not have group allocation.

Reporting for specific materials, systems and methods

We require information from authors about some types of materials, experimental systems and methods used in many studies. Here, indicate whether each material, system or method listed is relevant to your study. If you are not sure if a list item applies to your research, read the appropriate section before selecting a response.

Materials & experimental systems

n/a	Involved in the study	
☒	☐	Antibodies
☐	☒	Eukaryotic cell lines
☒	☐	Palaeontology and archaeology
☒	☐	Animals and other organisms
☒	☐	Human research participants
☒	☐	Clinical data
☒	☐	Dual use research of concern

Methods

n/a	Involved in the study	
☒	☐	ChIP-seq
☒	☐	Flow cytometry
☒	☐	MRI-based neuroimaging

Eukaryotic cell lines

Policy information about cell lines

Cell line source(s)	Hep G2 (ATCC® HB-8065™); HUVEC (ATCC® CRL-1730™)
Authentication	None of the cell lines used were authenticated.
Mycoplasma contamination	The cell lines were not tested for mycoplasma contamination.
Commonly misidentified lines (See ICLAC register)	/
